# Oxygen-Containing Quaternary Phosphonium Salts (oxy-QPSs): Synthesis, Properties, and Cellulose Dissolution

**DOI:** 10.3390/polym15204097

**Published:** 2023-10-16

**Authors:** Daria M. Arkhipova, Vadim V. Ermolaev, Gulnaz R. Baembitova, Aida I. Samigullina, Anna P. Lyubina, Alexandra D. Voloshina

**Affiliations:** 1 N.D. Zelinsky Institute of Organic Chemistry, Russian Academy of Sciences, Moscow 119991, Russia; samigullina@ioc.ac.ru; 2 Arbuzov Institute of Organic and Physical Chemistry, FRC Kazan Scientific Center of Russian Academy of Sciences, Kazan 420088, Russia; ermolaev@iopc.ru (V.V.E.); gulnaz.baembitova@mail.ru (G.R.B.); anna.lyubina@iopc.ru (A.P.L.); sobaka-1968@mail.ru (A.D.V.)

**Keywords:** oxygen-containing quaternary phosphonium salts, melting point, crystal structure, cellulose dissolution, antimicrobial properties, toxicity

## Abstract

In the present study, the synthesis of oxygen-containing quaternary phosphonium salts (oxy-QPSs) was described. Within this work, structure–property relationships of oxy-QPSs were estimated by systematic analysis of physical–chemical properties. The influence of the oxygen-containing substituent was examined by comparing the properties of oxy-QPSs in homology series as well as with phosphonium analog-included alkyl side chains. The crystal structure analysis showed that the oxygen introduction influences the conformation of the side chain of the oxy-QPS. It was found that oxy-QPSs, using an aprotic co-solvent, dimethylsulfoxide (DMSO), can dissolve microcrystalline cellulose. The cellulose dissolution in oxy-QPSs appeared to be dependent on the functional group in the cation and anion nature. For the selected conditions, dissolution of up to 5 wt% of cellulose was observed. The antimicrobial activity of oxy-QPSs under study was expected to be low. The biocompatibility of oxy-QPSs with fermentative microbes was tested on non-pathogenic *Saccharomyces cerevisiae*, *Lactobacillus plantarum*, and *Bacillus subtilis*. This reliably allows one to safely address the combined biomass destruction and enzyme hydrolysis processes in one pot.

## 1. Introduction

Ionic liquids (ILs) are a well-known part of organic salts consisting of a cation (imidazolium, ammonium, phosphonium, etc.) and an inorganic anion with a melting point commonly below 100 °C [1]. A unique set of properties, such as nonvolatility, nonflammability, thermal stability, and reusability, are more or less characterized by ionic liquids [2]. The combination of the useful properties and a broad diversity of structures make ILs widely used as solvents for reactions, including those catalyzed by nanocatalysts [3], as active pharmaceutical drugs [4], as antibacterial agents [5,6], and in the design of accumulators [7] and smart materials [8].

Considering the tremendous potential for a wide structural diversity, a reasonable stage of development was the emergence of task-specific ionic liquids (TSILs) [9]. This is a type of highly customized ILs that do not only serve as solvents; for example, through functionalization with oxygen-containing groups, their properties (viscosity, lyophilicity, hydrophilicity, hydrogen bonding ability, and electrical conductivity) can be easily tuned in a wide range, making them suitable for applications in organocatalysis, synthesis, gas absorption, supercapacitor development, and analytical chemistry [10,11,12,13]. Interest in the functionalization of ILs is still maintained, and the effect of the ether group on the physical constants and crystal packing is investigated [14].

One of the most abundant oxygen-containing phosphonium salts, terakis(hydroxymethyl)phosphonium chloride, is produced in multi-ton quantities and is widely used as a biocide and flame retardant, in the oil industry, in the leather industry as a tanning agent, and in the production and stabilization of nanoparticles [15]. Oxygen-containing phosphonium salts were quite effective solvents for the Grignard reaction [16]. Phosphonium salts with multiple ester groups in the side chain have demonstrated applicable properties (viscosity and ionicity) suitable for use as electrolytes in lithium-ion batteries [17] and have been established as an excellent reaction medium for lipase-catalyzed reactions [18]. Phosphonium salts with ester, ether, and hydroxyl groups as the fourth substituent are more suitable for biodegradation than tetraalkylphosphonium salts [19]. In this regard, professor Ragogna’s group demonstrated the antibacterial properties of the material containing functionalized phosphonium compounds [20,21].

The use of phosphonium salts as a cellulose dissolution agent has recently gained prominence. The ability of tetrabutylphosphonium 2-ethylhexanoate to dissolve cellulose from corn stover has been demonstrated. It is the best performing of the compounds used in this study, even over imidazolium compounds. At the same time, the authors refused to try to recycle the IL because of the challenges associated with the energy consumption of water evaporation [22].

The next generation of amino acid phosphonium ILs was introduced to dissolve cellulose for subsequent processing by enzymes [23]. Using the tetrabutylphosphonium N,N-dimethylglycine system and DMSO co-solvent, 15 wt% cellulose dissolution was achieved at 30 °C, and enzymatic conversion of dissolved cellulose to sugars was completed.

The quantitative structure–activity relationship (QSAR) model was developed for predicting cellulose solubility using the example of oxygen-containing phosphonium salts [24]. The designed model was successfully performed and showed an excellent R^2^ factor, but it did not include a parameter characterizing the high viscosity of resulting IL/cellulose mixtures. Nevertheless, a triphenylphosphonium salt containing a methoxymethyl moiety and acetate anion exhibited high cellulose solubility as predicted by the modeling. It has also been previously reported that the size and structure of the phosphonium cation play a dramatic role in various processes [25]. The search for the relationship between the ability of ILs to successfully participate in biomass preparation from various factors has long been underway. In general, comparisons are made from the structure and composition of cellulose or the composition of ionic liquids, their physical characteristics, and other parameters [26].

Earlier, we reported the synthesis and application in catalysis of sterically hindered ester containing phosphonium salt [27]. In the current work, we systematically studied the influence of the oxygen-containing substituents in cation on the physical–chemical properties of oxy-QPSs. The obtained trends were compared with the previously reported alkyl-substituted analog [28,29]. Some features of the crystal packing of oxy-QPSs were discussed. We utilized a series of compounds under the study for microcrystalline cellulose dissolution. The safety of oxy-QPSs for humans and the environment was also estimated.

## 2. Materials and Methods

### 2.1. Instrumental

NMR spectroscopy: NMR spectra were recorded using Fourier 300 HD instrument (Bruker, Zurich, Switzerland) at 21 °C (^1^H 300.1 MHz, ^31^C 75.5 MHz) and Avance NEO 300 instrument (Bruker, Zurich, Switzerland) at 21 °C (^31^P 121.5 MHz) with the residual solvent peak as an internal standard for ^1^H (CDCl_3_ 77.16).

Electro-spray ionization mass spectrometry (ESI-MS): ESI-MS measurements were performed using Maxis and MicroTOF II time-of-flight high-resolution mass spectrometers (Bruker Daltonic GmbH, Bremen, Germany) in positive mode in the mass range of *m*/*z* 50–3000. Direct syringe injection was used for all analyzed samples in acetonitrile solution at a flow rate of 5 μL/min. The capillary voltage was −4500 V, spray shield offset was −500 V, nitrogen nebulizer gas was −1 bar, nitrogen drying gas was 4 L·min^−1^, and desolvation temperature was 200 °C. The instruments were calibrated with a low-concentration tuning mixture (Agilent Technologies G2431A, Santa Clara, CA, USA). Data processing was performed by DataAnalysis 4.0 SP4 software (Bruker Daltonik GmbH, Bremen, Germany).

Thermogravimetry: A coupled system of a STA449-F3 TG/DSC synchronous thermal analysis instrument (Netzsch, Selb, Germany) with a Tensor 27 IR-Fourier spectrometer (Bruker, Ettlingen Germany) was used to study the decomposition temperature and thermal stability of the tested compounds. The samples (3.4–12.1 mg) were placed in aluminum crucibles with a perforated lid and heated in the range of 30–400 °C together with an empty crucible as a reference sample. TG/DSC measurements were carried out at a heating rate of 10 K/min in an argon flow of 50 mL/min. The resolution of Tensor 27 is 4 cm^−1^.

IR-spectroscopy: IR spectra were recorded on a Tensor-27 IR spectrometer (Bruker, Ettlingen, Germany). The survey was carried out at a resolution of 4 cm^−1^ (interval 4000–400 cm^−1^) in tablets with KBr.

Single crystal X-Ray analysis: The X-ray diffraction data for compounds were recollected at 100 K on a four-circle Synergy S diffractometer (Rigaku, Wrocław, Poland) equipped with a HyPix600HE area-detector (kappa geometry, shutterless ω-scan technique), using graphite monochromatized Cu K_α_-radiation; the intensity data were integrated and corrected for absorption and decay by the CrysAlisPro program [30]. The structures were solved by direct methods using SHELXT [31] and refined on *F*^2^ using SHELXL-2018 [32] or OLEX2 [33].

Optical microscopy: Optical microscope Micromed-1 (Saint-Petersburg, Russia) was used for the examination of cellulose dissolution.

### 2.2. Materials

All the work related to the preparation of the starting substrates, as well as the synthesis and the workup of products, was carried out in an inert N_2_ atmosphere using the standard Schlenk apparatus. All solvents and purchased reagents were absoluted by the appropriate methods, mainly by distillation in an inert atmosphere.

Tri-*tert*-butylphosphine was purchased from Dalchem (Nizhniy Novgorod, Russia). Cellulose microcrystalline (average particle size 50 µm) was purchased from Acros Organics (Morris Plains, NJ, USA). Halogenated acids were purchased from Sigma-Aldrich (St. Louis, MO, USA), and halogenated ethers and esters were purchased from Alfa Aesar (Heysham, Lancashire, UK).

### 2.3. General Procedure of the Synthesis of oxy-QPSs

Tri-*tert*-butylphosphine and alkylating agent were stirred at 50–100 °C for 4–8 h. The reaction mixture was cooled; white solid was washed with diethyl ether (see Supplementary Materials for details). For couple of syntheses, the solvent acetonitrile was used. After cooling, the reaction mixture solvent was evaporated and then washed with diethyl ether.

### 2.4. Screening Dissolution of Microcrystalline Cellulose in oxy-QPSs

1 mg of microcrystalline cellulose (50 μm, Avicell), 50 mg of oxy-QPS, and 47 µL of DMSO were added to the 5-mL glass tube equipped with magnetic stirring bar. The reaction vessel was sealed with a screw cap. The process was carried out at 80 °C. For a certain time interval, a drop of solution was collected and placed on glass slide. Monitoring the dissolution of cellulose was administrated by direct observation using optical microscope at 40–100× magnification.

### 2.5. Antimicrobial and Antifungal Activity of oxy-QPSs

The antimicrobial activity of the oxy-QPSs was determined by serial dilution technique in Mueller–Hinton broth 2 for pathogenic bacteria, MRS medium for the cultivation of lactobacilli, and Sabouraud broth for yeast. The cultures used for testing included the Gram-positive bacteria *Staphylococcus aureus* ATCC 6538P FDA 209P, *Bacillus cereus* ATCC 10702 NCTC 8035, *Enterococcus faecalis* ATCC 29212; Gram-negative pathogenic bacteria *Escherichia coli* ATCC 25922; Gram-positive non-pathogenic endospore-forming bacteria *Bacillus subtilis* ATCC 6633; and pathogenic yeast *Candida albicans* ATCC 10231 from the State Collection of Pathogenic Microorganisms and Cell Cultures “SCPM-Obolensk”. Gram-positive non-pathogenic non-spore-forming bacteria Lactobacillus plantarum 8P-A3 and non-pathogenic yeast *Saccharomyces cerevisiae* 1986 were from the collection of Kazan Federal University. The bacterial load was 3.0 × 10^5^ CFU/mL. The fungal load was 2.0 × 10^3^ CFU/mL. Results were recorded every 24 h for 5–7 days. Bacterial cultures were incubated at 37 °C, and yeast cultures were incubated at 25 °C. The experiment was repeated three times. Compound dilutions were prepared immediately on nutrient media. The dilutions of the oxy-QPSs were prepared immediately in nutrient media; 5% aqueous solution of DMSO was used to improve the solubility of the oxy-QPSs, and the test strains were not inhibited at this concentration. The minimum inhibitory concentration (MIC) was defined as the minimum concentration of a compound that inhibits the growth of the respective test microorganism [34,35]. The growth of bacteria, as well as the absence of growth due to the bacteriostatic action of a compound, was recorded. To determine the minimum bactericidal concentration (MBC), an aliquot of the culture of pathogenic bacteria was transferred onto Mueller–Hinton agar in a 10 cm Petri dish and incubated for 24 h at 37 °C. The MBC was the minimum concentration at which bacterial colonies were not detected, indicating that the bacteria were killed with >99.9% efficiency.

### 2.6. Toxicity of oxy-QPSs

#### 2.6.1. Hemolytic Activity

The hemolytic activity of oxy-QPSs was estimated by comparing the optical density of a solution containing the test compound with that of blood at 100% hemolysis. The experiments were carried out as described earlier [36].

#### 2.6.2. Cell Toxicity Assay (MTT Assay)

The cytotoxic effect of the test compounds on normal human cells was determined using the colorimetric method of cell proliferation MTT (Thiazolyl Blue Tetrazolium Bromide, Sigma, Kawasaki, Japan). For this purpose, 10 μL of MTT reagent in Hank’s balanced salt solution (HBSS) (final concentration 0.5 mg/mL) was added to each well. The plates were incubated at 37 °C for 2–3 h in an atmosphere humidified with 5% CO_2_. Absorbance was recorded at 540 nm using a microplate reader (Invitrologic, Novosibirsk, Russia). Experiments for all compounds were repeated three times. The Chang liver cell line (human liver cells) from N. F. Gamaleya Research Center of Epidemiology and Microbiology (Moscow, Russia) was used in the experiments. The cells were cultured on a standard nutrient medium, “Igla”, produced by the Moscow Institute of Poliomyelitis and Viral Encephalitis. M.P. Chumakov (Moscow, Russia) with the addition of 10% fetal calf serum and 1% nonessential amino acids (NEAA) (PanEco, Moscow, Russia).

The cells were plated on a 96-well panel (Eppendorf SE, Hamburg, Germany) at a concentration of 5 × 10^3^ cells per well in a volume of 100 μL of medium and cultured in a CO_2_ incubator at 37 °C. In 48 h after planting the cells, the culture medium was taken into the wells, and 100 μL of solutions of the studied oxy-QPSs in the specified dilutions were added to the wells. Dilutions of the compounds were prepared directly in growth medium supplemented with 5% DMSO to improve solubility. The cytotoxic effect of the test compounds was determined at concentrations of 0.1–100 μM. The calculation of the IC_50_, the concentration of the drug causing inhibition of cell growth by 50%, was performed using the program MLA “Quest Graph™” IC_50_ Calculator [37].

#### 2.6.3. Ecotoxicity to *D. magna*

The test culture *Daphnia magna* was used to determine the LC_50_ of the studied oxy-QPSs. The crustaceans were kept under standard conditions according to the recommendations published in ISO 6341 [38] and NBR 12713 [39]. The sensitivity to K_2_Cr_2_O_7_ was determined one week before the test; LC_50_ for potassium dichromate was 1.443 mg/L.

A total of 10 crustaceans were planted in each falcon with the studied solutions; the glasses were kept in a luminostat for 48 h at a temperature of 22 °C, with a 12 h illumination regime at an intensity of 600 lux. The pH of the solutions was 7.2–7.4, and the O_2_ content was 6.8–7.2 mg/L. The crustaceans were not fed the day before the study and during the test.

After 48 h, a visual count of surviving daphnia was performed [40]. The results were recorded in the protocol and calculated using the program “R” version 2.13.0 (13 April 2011). All compounds under the study at 1% stock concentration were readily soluble in distilled water. Further dilutions were made with biogenerated water. At the same time, a control group of daphnia was placed in biogenerated water without any of the studied substances. All the daphnia in the control group survived.

The test substances were classified as Acute 1, Acute 2, or Acute 3 to aquatic organisms based on LC_50_ definitions [41].

## 3. Results and Discussion

### 3.1. Synthesis of oxy-QPSs

**Scheme 1:**
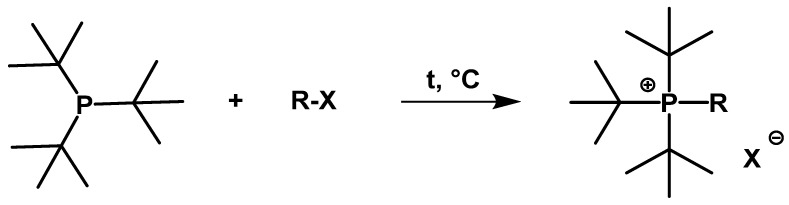
The general reaction of oxy-QPS synthesis.

The synthesis of oxygen-containing quaternary phosphonium salts (oxy-QPSs) involves the quaternization reaction of tertiary phosphine and halogenated reagent, as in the case of alkyl-substituted QPSs. Ethers, carboxylic acids, and esters were applied as the functional groups in the cation (Scheme 1, Table 1). The coupling reaction requires the usage of an inert atmosphere due to the high sensitivity of initial tri-*tert*-butylphosphine to atmospheric oxygen and humidity. In turn, the reactivity of the alkylating agent reduces as chain length increases. In this respect, the synthesis conditions were harsher when a longer halogenated reagent came into the reaction (see Supplementary Materials for experimental details). According to the atom economic principle, the quaternization reactions were carried out without a solvent in most cases except for the synthesis of oxy-QPSs **7** and **8** containing carboxylic groups in the side chain. Acetonitrile was involved in these runs to exclude the formation of side products caused by the interaction of acidic proton with phosphorus.

The oxy-QPSs **1** and **4** were introduced into the metathesis reaction to give **2** and **5**–**6** included tetrafluoroborate- or hexafluorophosphate-anions. The procedure took place in an aqueous solution to ensure complete conversion due to the insolubility of products in water.

The oxy-QPSs with halogen anions were found to be soluble with most polar organic solvents (chloroform, DMSO, acetone, ethanol, etc.) and water. The compounds **1** and **3**–**4** are also soluble in diethyl ether and other non-polar solvents as well. The phosphonium salts **2** and **5**–**6** are poorly dissolved in diethyl ether.

### 3.2. NMR Study

#### 3.2.1. ^31^P NMR Spectral Data

The ^31^P{^1^H} NMR spectra of oxy-QPSs exhibited a singlet in a range of 44.0–52.0 ppm (Table 2), except for **6**, where the signal was accompanied by a septet of a hexafluorophosphate group. The chemical shift of the compounds depends on the proximity of the oxygen atom to the phosphorus one. The chemical shift of **1**–**2** and **4**–**6** appears in higher magnetic fields in narrow regions (44.0–44.7 ppm) due to the compensation of electronic density withdrawn by the oxygen of the ether group. The ^31^P{^1^H} NMR spectra of **7** and **8** are around 50 ppm and close to those for alkyl-substituted sterically hindered phosphonium salts. The anion weakly influenced the chemical shift in ^31^P{^1^H} NMR spectra; the difference is 0.4 and 0.5 ppm for **1**–**2** and **4**–**6**, consequently.

#### 3.2.2. ^1^H NMR Spectral Data

**Figure 1:**
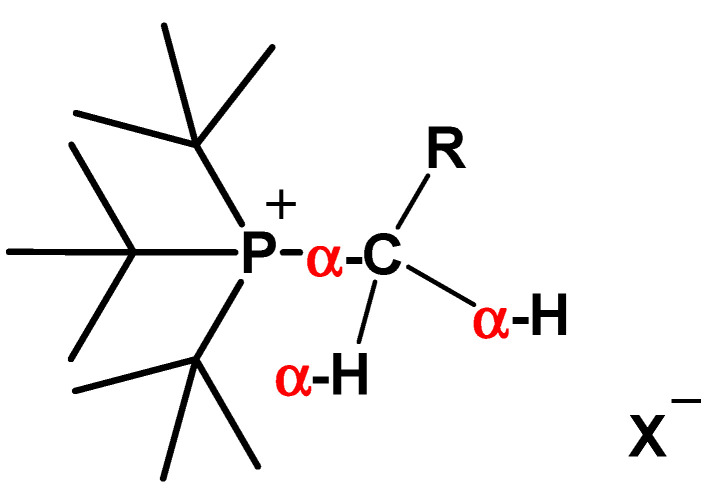
α-protons and α-carbon in oxy-QPSs.

Both sterically hindered QPSs with the linear alkyl substituent and oxy-QPS α-protons are the most sensitive to the structure modification (Figure 1). The signal of α-protons in ^1^H NMR spectra exhibited a multiplet and varies depending on the functional group neighboring to (Table 2). The chemical shift to the high field is characteristic of the substituents where α-protons are separated by three single bonds or more from the oxygen atom (for **3**, **7**–**8**).

Anion exchange influences the chemical shift of α-protons as well. The signal in phosphonium tetrafluoroborate or hexafluorophosphate appears in a higher magnetic field (4.55–4.62 ppm) than in halides (5.11–5.17 ppm). The chemical shift of α-protons in oxy-QPSs with carboxylic group **9**–**12** is observed in a range of 4.07–4.27 ppm.

#### 3.2.3. ^13^C NMR Spectral Data

The most influenced carbon atom in oxy-QPSs is the α-carbon nearest to the phosphorus one in the functional substituted group (Figure 1, Table 2). The α-carbon between phosphorus and oxygen atoms in **1**–**2** and **4**–**6** is the most deshielded and presented in ^13^C{^1^H} NMR spectra as a doublet in a range of 59.8–61.9 ppm. The influence of the anion on the chemical shift of α-carbon is about 0.6 ppm.

### 3.3. IR

**Figure 2:**
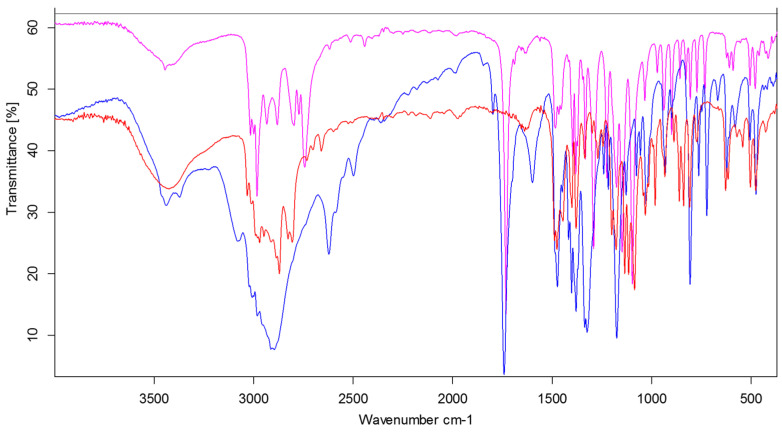
IR spectra of oxy-QPSs **4** (tri-*tert*-butyl((2-methoxyethoxy)methyl)phosphonium chloride) (red), **8** (tri-*tert*-butyl(5-carboxypentyl)phosphonium bromide) (blue), and **11** (tri-*tert*-butyl(2-*iso*-propoxy-2-oxoethyl)phosphonium bromide) (purple).

The IR spectral data of oxy-QPSs showed bands in the region 810–800 cm^−1^, which was associated with the stretching vibrations of the P-C groups. Other bands were observed at around 2910–2980 cm^−1^, which were assigned with the stretching vibrations of the dangling CH_2_/CH_3_ groups (Figure 2, Figures S37–S48 in Supplementary Materials).

For compounds **1**–**6** containing ether functional groups, bands of significance were around 1200–1050 cm^−1^, which were due to stretching antisymmetric vibrations of the C-O-C groups. The spectra of **1** and **3** have absorbance at 1182 and 1097 cm^−1^ and 1175 and 1094 cm^−1^, respectively. Four bands were assigned for **4**–**5** in the same region: 1178, 1135, 1116, and 1086 cm^−1^.

Strong absorbance at 1734 and 1741 cm^−1^ was associated with the stretching vibrations of the C=O groups in **7** and **8**. The stretching vibrations of the C=O ester groups in **9**–**12** have absorbance at 1740, 1735, 1732, and 1727 cm^−1^, accordingly.

Weak absorbance at around 3440 cm^−1^ was associated with the stretching vibrations of the COO-H groups of **7**–**8**. Broadening of these bands indicates the hydrogen bond formation, which is correlated well with the analysis of crystal packing of oxy-QPSs.

The FTIR spectrum of the **9**–**12** has strong absorbance at 1320 and 1160 cm^−1^, which was assigned to the stretching symmetric and antisymmetric vibrations of C-O groups.

### 3.4. DSC Measurements and TG Analysis

The melting points (T_m_) and decomposition temperatures (T_d_) were measured by differential scanning calorimetry and thermogravimetric analysis (Table 3, Figures S49–S60 in Supplementary Materials). One can define studied compounds into three main homologous series. The limited range of available series does not allow us to demonstrate all the trends in melting points and decomposition temperatures; however, some tendencies could be noticed. Commonly, the melting temperature depends on various factors. Both the higher symmetry of the structure and the presence of donor–acceptor groups in the molecule may result in an increase in the lattice energy and, as a consequence, in the rise of the melting and decomposition temperature [42].

Typically, an elongation of the substituent in cation leads to a reduction in the melting point. Our study agrees with this claim (for example, **1** and **3**). On the contrary, an additional oxygen atom in the side chain increases the melting temperature in spite of an overall chain length growth (**1** and **4**). This effect is related to the rising interactions between the chains through weak hydrogen bonds driven by an additional oxygen atom [43,44]. As expected, replacing the halide anion typically strongly bonding to the cation with a weakly coordinating tetrafluoroborate anion led to a decrease in the melting point. Here, we observe that the melting point of **5** and **6** drops by half compared to **4**. Notably, the decomposition temperature increases slightly from 187 to 210 and 221 °C, respectively.

The oxy-QPSs with the ester functional group (**9**–**11**) melt with decomposition at around 170 °C with a loss of about half their mass. Probably, the same mechanism of thermal decomposition takes place involving the detachment of the acetoether fragment. Compound **12,** with a total substituent length of seven atoms, has a phase transition at 125 °C. This is in agreement with the previously observed lengthening effect of the alkyl substituent for sterically hindered phosphonium salts and with the literature data for other organic salts [45], including the calculated data [46].

**Figure 3:**
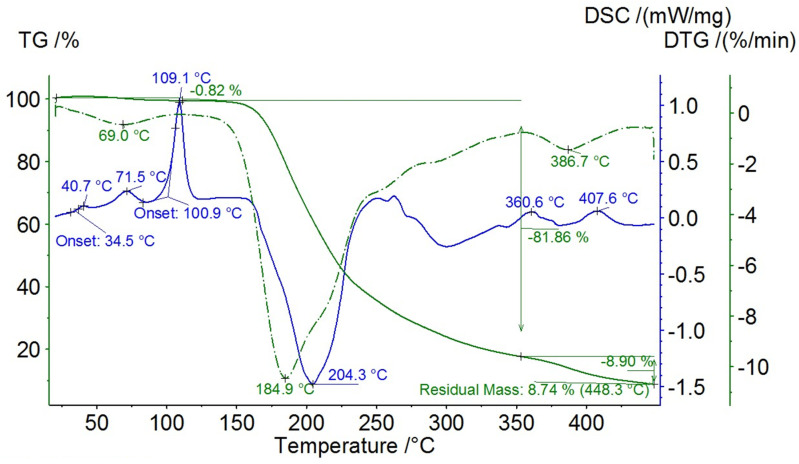
TG-DSC curves for **3** (tri-*tert*-butyl(2-ethoxyethyl)phosphonium bromide): TG curve (green line), DSC curve (blue line), and DTG curve (green dashed line).

It is worth mentioning that the DSC curve of **3** has two more phase transitions at temperatures 41 °C and 71 °C, accordingly, prior to complete melting at 109 °C (Figure 3). These phase transformations occur without the loss of mass.

### 3.5. Single Crystal X-ray Analysis

Crystals suitable for analysis by single crystal X-ray diffraction were obtained only for some compounds from the presented series. The main crystallographic information for oxy-QPSs is provided in Supplementary Materials. The analysis and comparison of the features of molecular and crystal structures were carried out within the three main homologous series, and some general patterns can be noted.

**Figure 4:**
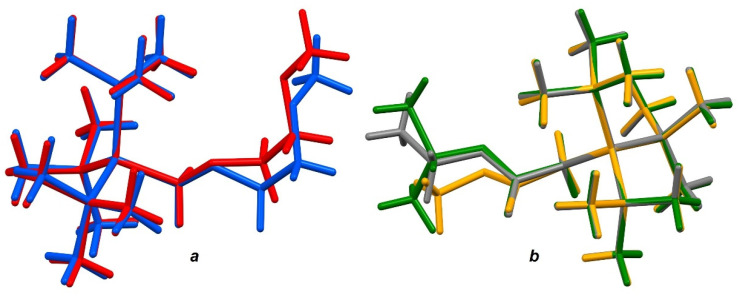
The conditional overlay of oxy-QPS cations: (**a**) **4** (red) and **6** (blue); (**b**) **9** (yellow), **10** (grey), and **11** (green). The superimposition is performed on the phosphorus atom and the four nearest atoms.

All compounds form monoclinic or orthorhombic crystals without solvent molecules in the crystal lattice and with one cation–anion pair in the independent part of the unit cell. Compound **6** is the only oxy-QPS in the investigated series that exhibits a partial disorder in the linear substituent of the cation, as well as possesses the entire anion. Such behavior may be attributed to the nature of the substituent in cation. The introduction of oxygen atoms leads to the flexibility of the side chain of oxy-QPSs and causes a slight change in the conformation in the crystal, even in the case of the same type of cation, for example, in crystals **4** and **6** (Figure 4a). Meanwhile, in the crystals of compounds **7** and **8**, the alkyl part of the substituent is in a characteristic zigzag conformation (see Figures S64 and S65 in Supplementary Materials). In the homologous series of compounds **9**–**11**, the conformations of the side chain are fairly very close despite the differences in their length (Figure 4b).

In the crystals of compounds **7** and **8**, an uncharacteristic conformation of the carboxyl group can be noted. The hydrogen atom of the –COOH fragment is in the *trans* position with respect to the C=O group (see Figures S64 and S65 in Supplementary Materials). This orientation prevents the formation of a classical centrosymmetric dimer, which is characteristic of molecules with a carboxyl fragment. As a result, the classical hydrogen bonds of the O–H⋯Br type are realized in both cases.

In the example of COOH-functionalized imidazolium ionic liquids, it was shown that in the crystals of hydrophilic ionic liquids, the formation of classic O–H⋯X (X = Halide) bonds between the cation and anion of the system is preferred. Classical dimers between two cations can be realized only for hydrophobic ionic liquids. Nevertheless, in both groups of ionic liquids, the classical *cis*-conformation of the carboxyl group is realized [47]. In our case, another type of conformation of the carboxyl system leads to some shortening of the donor–acceptor distance. The O⋯Br^−^ distances of the O–H⋯Br^−^ hydrogen bonds are 3.1124(12) Å and 3.177(2) Å in crystals **7** and **8**, respectively.

**Figure 5:**
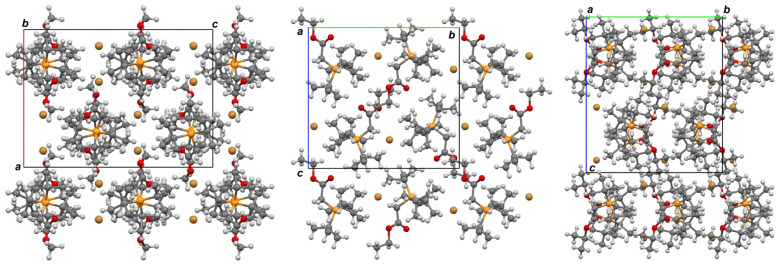
The fragments of crystal packing of oxy-QPSs from left to right: **9** (view along the *0b* axis), **10** (view along the *0a* axis), and **11** (view along the *0a* axis). The Mercury program suite [48] was used for molecular graphics.

The crystal structure of oxy-QPSs essentially depends on the type of side chain substituent and on the type of anion. Specifically, the compounds form non-isostructural crystals, so no one can observe any trend in the features of crystal packing. For example, compounds **9**–**11** with homologically close cations and identical anions are characterized by a different three-dimensional lattice and localization of anion (Figure 5). 

### 3.6. Microcrystalline Cellulose Dissolution

Recently, lignocellulosic biomass treatment has gained a reputation as a key process of near-future chemical technology [49,50]. Biomass recycling provides access to renewable feedstock for energy, chemicals, and advanced materials [51,52,53]. Polysaccharides are the major structural component of plant cell walls. The challenging step of the recycling process is biomass pretreatment due to the poor solubility of natural polysaccharides in water and organic solvents [54,55]. The most common carbohydrate polymers are cellulose, chitin, chitosan, and starch. The ILs (organic salts in a broad sense) have found an application in the field of cellulose and other polysaccharides’ dissolution, and a number of advances have been made [56,57].

Among effective ILs in biomass pretreatment are nitrogen-containing heterocycles and ammonium salts with oxygen-containing functional groups [58,59,60]. However, the high efficiency in cellulose dissolution has been demonstrated by the phosphonium salts as well [24,61]. Recent research has highlighted a number of structure–capacity patterns for effective cellulose dissolution in organic salts, which have been emphasized [62,63]. Regardless, the strictly defined structural motives for IL design in this area do not exist.

**Figure 6:**
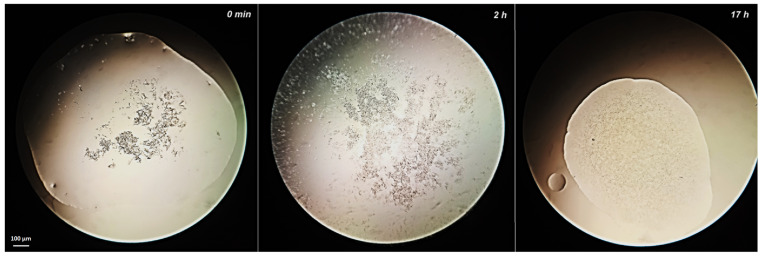
Microcrystalline cellulose (1 wt%) in DMSO at 80 °C.

In the first series of experiments, we used phosphonium salts under the study to dissolve microcrystalline cellulose. As oxy-QPSs are solids at room temperature and melt above 100 °C, a co-solvent was used to reduce the viscosity. Mixtures of ILs and organic solvents are extensively used for biomass pretreatment [64]. The co-solvent also acts to decrease the cost of the dissolution process. The most popular of them is dimethyl sulfoxide (DMSO). It is an aprotic, inexpensive, and non-volatile organic solvent that is obtained as a by-product of the pulping industry in lignin production [65]. DMSO is readily miscible (1:1 by mass) with most of the oxy-QPSs under study. DMSO has no significant influence on the specific interactions between organic salts and dissolved cellulose [66]. Stirring of 1 wt% microcrystalline cellulose (50 µm) in pure DMSO at 80 °C for 17 h did not lead to the dissolution of the biopolymer (Figure 6).

Microcrystalline cellulose of 50 µm (1 wt%), the mixture of corresponding oxy-QPSs and DMSO (1:1 by mass), was placed in a 5 mL Duran test tube with a magnetic stirrer and tightly screwed cap. The mixture was stirred at 800 rpm at 80 °C. At certain time intervals, a drop of the mixture was picked and placed on a glass slide, and the solubility process was analyzed under an optical microscope at 40× magnification (Table 4).

**Figure 7:**
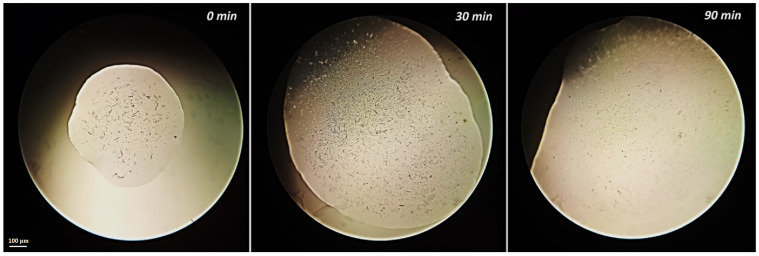
Microcrystalline cellulose (1 wt%) in the mixture of **3** and DMSO (1:1) at 80 °C.

During the experiment, the cellulose fibers split into smaller ones, then visible particles disappeared, and a homogeneous solution was observed (Figure 7).

**Figure 8:**
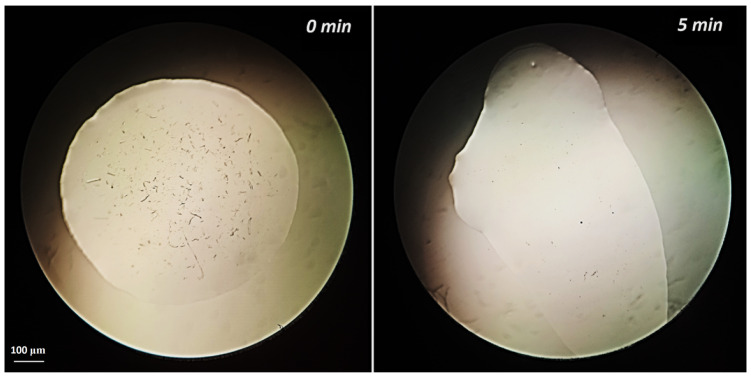
Microcrystalline cellulose (1 wt%) in the mixture of **1** and DMSO (1:1) at 80 °C.

The mixtures of **1** and **4** with DMSO partly dissolve cellulose fibers during heating till 80 °C. After stirring for 5 min at 80 °C, the solution became clear with no visible particles of cellulose (Figure 8).

The mixture of **3** and DMSO dissolves cellulose slower than two other oxy-QPSs with the ether functional group and halide anion. Polymer fibers disappear after 90 min of heating (Figure 7). However, at this time, oxy-QPS **3** is partly destructed, confirmed by the ^1^H NMR spectrum. The destruction occurs with the loss of one of four substituents, and tertiary phosphonium salt is formed. We suppose that compounds **1** and **4,** with their similar structure, possess chloride anions that probably make the molecule more stable under process conditions due to the small size of the anion and stronger ionic binding. At 80 °C, the phosphonium compounds **9** and **11** are not fully miscible with DMSO, preventing cellulose dissolution. The heating was terminated after 4 h. The destruction of **10** and **12** is detected by the NMR method after several hours of heating, although some splitting of the cellulose fibers is observed. The mixture of **5** and DMSO dissolves cellulose slowly; probably, the appearance of tetrafluoroborate-anion in oxy-QPS structure prevents the formation of hydrogen bonds with cellulose molecules.

In the next step of the study, oxy-QPSs with the ether group and halide anion were introduced into the dissolution process at a reduced temperature (Table 5). The temperature decrease to 60 °C became the breaking point when the cellulose fibers showed no visible dissolution for up to 8 h. In the example of **3**, one can see gradual moderation in the time of cellulose splitting and disappearing. Oxy-QPS **4** appeared not to be fully miscible with DMSO at 60 °C, which influenced its solubilizing properties on cellulose.

The distinctive feature made **4** the solvent of choice for carrying out a number of experiments with the aim of optimizing the process of cellulose dissolution. The first enhancement was made to reduce the ratio of the oxy-QPSs to DMSO. When the proportion of oxy-QPSs to DMSO was 1:4, the time of cellulose dissolution increased from 5 min to 4 h at 70 °C.

The same effect of elongation of dissolution time was observed when DMSO was substituted with water. An amount of 1 wt% of cellulose and the mixture of **4** and water (1:1) was heated to 70 °C, and the complete dissolution of polymer fibers was observed after 4 h of stirring.

Due to the high solubility of **4** in water, a three-component system was tested for cellulose dissolution. The mixture of **4**, DMSO, and water (2:1:1) with 1 wt% of cellulose were stirred at 60 °C. In contrast to the two-component mixture of **4** and DMSO, the oxy-QPS was fully miscible with co-solvents when half of DMSO was replaced by water. However, after 8 h of stirring, cellulose fibers had not been dissolved in the mixture.

Taking into account the ready dissolution of cellulose in the mixtures of **1** and **4** with DMSO at 80 °C, the enlarged loading of cellulose was tested. Each of the two mixtures had soluted up to 5 wt% of cellulose for around 30 min. It is noteworthy that the resulting solution of cellulose in **4** + DMSO is less viscous than in **1** + DMSO.

The best solvent for cellulose among studied oxy-QPSs is the mixtures **4** + DMSO and **1** + DMSO. Both **1** and **4** possess ether functional groups in cation and Cl-anion. The chloride anion, due to its small size, acts as a hydrogen acceptor, facilitating the dissolution of cellulose. The oxygen atom in **1** and **4** is structurally tethered in the vicinity of the charged phosphorus closer than in other oxy-QPSs.

Hydrogen bonds play a very important role in processes where ionic liquids are used as solvents. To date, numerous studies have indicated that hydrogen bonding formation between anions and the –OH group of cellulose is the main driving force for cellulose dissolution in ILs [58,59,60,64]. The geometrical neighborhood of the charged head group could have an electronic impact on H-bonding [67]. Indirect evidence of the strong influence of functional groups on the possibility of changing the strength of hydrogen interactions can be changes in chemical shifts in ^1^H NMR spectra. This is exactly what we see in the case of compounds **1** and **4**, where the protons of the CH_2_ fragments “tear” between the positive charged phosphonium cation and the electronegative oxygen atom, which leads to a shift of signals of corresponding protons to the region of 5 ppm. Of course, one cannot discount the small chloride anion, which makes a significant contribution to the interaction with the bare nuclei of protons [68]. Thus, in phosphonium salts **1** and **4**, we obtained the necessary combination of critical structural components for the formation of strong hydrogen bonds, which allows us to successfully dissolve cellulose.

### 3.7. Antimicrobial Activity of oxy-QPSs

Despite spreading opinion, not all ILs are toxic in the same way [43,57], although it is generally known that phosphonium salts have quite high antibacterial activity [69], which is reflected in their use to create an antibacterial coating for plastic surfaces [70] or even create polymers with antibacterial properties [71]. At the first approximation, the biological activity of oxy-QPSs was estimated on several pathogenic bacteria and fungi as the model systems. The phosphonium salts under study were tested for antimicrobial activity against a number of Gram-positive *Staphylococcus aureus* (*Sa*), *Bacillus cereus* (*Bc*), and *Enterococcus faecalis* (*Ef*) and Gram-negative *Escherichia coli* (*Ec*) bacteria. Antifungal activity was studied on a culture of a yeast-like fungus, *Candida albicans* (*Ca*). The results on antimicrobial activity of oxy-QPSs are presented in Table 6.

Based on the data obtained, one can conclude that the tested compounds have a selective effect on the Gram-positive bacteria *S. aureus*. The most effective compounds were **7** and **10**, which showed antimicrobial activity at the level of the reference drug chloramphenicol. Chloramphenicol inhibits bacterial protein synthesis by interfering with the ‘transfer’ of the elongated peptide chain to the newly attached aminoacyl t-RNA at the ribosome-RNA complex. It binds specifically to the 50 S ribosome and may prevent the aminoacyl t-RNA from accessing the acceptor site for amino acid incorporation. Chloramphenicol probably acts as a peptide analog, preventing the formation of peptide bonds. At high doses, it can also inhibit mammalian mitochondrial protein synthesis. Marrow cells are particularly susceptible [72,73,74,75].

It was also shown that **7** and **10** have weak bactericidal activity (the difference between MIC and MBC did not exceed 4) against *S. aureus 209p*, in contrast to chloramphenicol, which is a bacteriostatic drug. The test compounds were inactive against *Bacillus cereus*, *Enterococcus faecalis*, *Escherichia coli*, and *Candida albicans*.

The fermentation process could be included in a one-pot biomass treatment or follow the dissolution procedure in a stage-by-stage process. Both of them require the usage of non-toxic solvents due to the microorganisms that are utilized during the fermentation. The biocompatibility of ILs is a serious problem for the choice of an effective biomass solvent [76]. The tolerance study of potential solvents against fermented microbes should be an essential procedure [77].

The biocompatibility of the oxy-QPSs with enzymatic microorganisms was tested against non-pathogenic microbes that could be used in the fermentation process (Table S1 in Supplementary Materials): *Saccharomyces cerevisiae 1986* (*Sc*), *Lactobacillus plantarum 8P-A3* (*Lp*), and *Bacillus subtilis ATCC 6633* (*Bs*). The obtained results demonstrated low toxicity of oxy-QPSs against all non-pathogenic microorganisms. All tested compounds were absolutely inactive (MIC > 500 µg/mL) against *Saccharomyces cerevisiae* and *Lactobacillus plantarum*. Compounds **1**, **7,** and **9** showed negligible activity against *Bacillus subtilis* bacteria compared to the antibiotic amoxicillin. Their MIC manifested in the range of 250–500 µg/mL.

The oxy-QPSs were shown to be a suitable environment for bacteria and fungi and could be safely used in a biomass pretreatment process followed by fermentation. It would be safe to assume that IL utilization prevents the formation of inhibitors for the growth of microorganisms that are capable of enzymatic biomass degradation.

### 3.8. Cytotoxicity of oxy-QPSs

The impact of ILs on living cells is an important challenge to explore in the development of the strategy for their industrial application [78]. As a part of the study of the safety of compounds for humans, the hemolytic and cytotoxic activities of oxy-QPSs were evaluated. The ability of the tested compounds to destroy human erythrocytes illustrates the toxic effect on the internal environment of the body. The hemolysis assay is a simple screening test that can represent the cytotoxicity in more complex models [79]. Such experimental models can be cell lines derived from different human organs and tissues and provide an adequate assessment of the effect of the compounds on cell metabolism.

The cytotoxicity of oxy-QPSs was studied using human erythrocytes, normal liver cell line Chang liver, and human embryo lung WI38. Within the study, the values of HC_50_, the concentration causing hemolysis of 50% of erythrocytes, and IC_50_, the concentration causing the death of 50% of human liver cells, were determined (Table 7).

According to the test results, all tested oxy-QPSs showed no hemolytic activity and low toxicity to normal human cell lines, which characterizes their safety toward mammalian cells.

### 3.9. Ecotoxicity of oxy-QPSs

The industrial use of a new solvent requires the checking of its safety. Modern solvents for natural polysaccharides should be safe, non-toxic, and nonhazardous to humans and the environment [76].

The planktonic crustacean *Daphnia magna* is a widespread water animal that possesses known sensitivity to the slightest environmental change and, therefore, is a convenient organism in ecotoxicological research. One of the factors that profoundly affects the survival rate of *D. magna* is environmental pollution.

The acute aquatic toxicity of oxy-QPSs was determined using a *D. magna* population. A 48 h test was performed with selected water solutions of oxy-QPSs at different concentrations (Table S2 in Supplementary Materials). Each test involved 30 mature animals. The compounds with shorter substituents (**1** and **7**) could be classified as nonhazardous according to the Globally Harmonized System of Classification and Labelling of Chemicals. The oxy-QPSs **4**–**5** and **11**–**12** are referred to in the category Acute 3.

## 4. Conclusions

We synthesized new quaternary phosphonium salts with oxygen-functionalized groups in sterically hindered cations. The NMR, FTIR, and TG/DSK study and crystal structure showed that trends in properties of oxy-QPSs are, to some extent, similar to those for alkyl-QPSs. However, the introduction of the oxygen atom in the side chain is responsible for the hydrogen bond formation, which causes some novel tendencies in properties, such as melting point or chemical shift in NMR spectra.

The structural features were found to determine the ability of oxy-QPSs to dissolve microcrystalline cellulose. The ether group in the cation and chloride anion was found to be necessary for effective cellulose dissolution. Notably, the oxygen atom in the side chain is preferred to be no more than one carbon atom across from phosphorus. This is attributed to the acidity of the α-protons and the strong hydrogen bond formation. The conditions of the dissolution process, such as temperature, time, co-solvent, cellulose loading, and solvent ratio, were selected. Pretreatment with ether-containing phosphonium salts and DMSO co-solvent (1:1) results in the dissolution of up to 5 wt% of microcrystalline cellulose at 80 °C for 30 min. The use of **4** allows DMSO to be replaced by water for cellulose dissolution but with a significant increase in process time.

The antimicrobial activity of the oxy-QPSs under investigation was expected to be low due to the short length of substituents in the cation. The studied compounds are sufficiently safe for the non-pathogenic microbes that can stand as fermenting ones in a one-pot biomass treatment process. The current study has also shown the low toxicity of oxy-QPSs to the human cell lines and to the crustacean *D. magna*. The present work can be used to guide the design of new task-specific phosphonium salts.

## Figures and Tables

**Table 1:** The structure of oxy-QPSs.

oxy-QPSs	R	X
**1**	CH_2_-O-CH_3_	Cl
**2**	CH_2_-O-CH_3_	BF_4_
**3**	CH_2_-CH_2_-O-CH_2_-CH_3_	Br
**4**	CH_2_-O-CH_2_-CH_2_-O-CH_3_	Cl
**5**	CH_2_-O-CH_2_-CH_2_-O-CH_3_	BF_4_
**6**	CH_2_-O-CH_2_-CH_2_-O-CH_3_	PF_6_
**7**	CH_2_-CH_2_-COOH	Br
**8**	CH_2_-CH_2_-CH_2_-CH_2_-CH_2_-COOH	Br
**9**	CH_2_-C(O)-O-CH_3_	Br
**10**	CH_2_-C(O)-O-CH_2_-CH_3_	Br
**11**	CH_2_-C(O)-O-CH(CH_3_)_2_	Br
**12**	CH_2_-C(O)-O-CH_2_-CH_2_-CH_2_-CH_3_	Br

**Table 2:** The selected chemical shifts of oxy-QPSs in ^31^P, ^1^H, and ^13^C NMR spectra (CDCl_3_). See Figures S1–S36 in the Supplementary Materials.

oxy-QPSs	δ in ^31^P NMR Spectra (ppm)	δ of α-H in ^1^H NMR Spectra (ppm)	δ of α-C in ^13^C NMR Spectra (ppm)
**1**	44.4 (45.8) *	5.11	61.9
**2**	44.0	4.57	61.3
**3**	51.6 (52.4)	2.95	20.2
**4**	44.7 (46.0)	5.17	60.5
**5**	44.3 (45.5)	4.62	59.8
**6**	44.2	4.55	59.8
**7**	50.0 (51.3)	2.75	13.6
**8**	49.7	2.47	18.7
**9**	51.9 (53.4)	4.27	25.5
**10**	51.9 (53.5)	4.13	25.5
**11**	52.0 (53.6)	4.10	25.8
**12**	52.0 (53.4)	4.07	25.4

* The chemical shift in non-deuterated acetone is given in brackets.

**Table 3:** Summary of melting points T_m_ (by DSC) and decomposition temperatures T_d_ (by dynamic TGA) for oxy-QPSs.

oxy-QPSs	T_m_, °C	T_d_, °C
**1**	144	171
**2**	142	236
**3**	109	185
**4**	178	187
**5**	92	210
**6**	87	221
**7**	173	210
**8**	186	186
**9**	170	170
**10**	168	172
**11**	170	170
**12**	125	167

**Table 4:** The time of cellulose dissolution in oxy-QPSs.

The Mixture	Time of Cellulose Dissolution *	Note
**1** + DMSO	5 min	-
**3** + DMSO	90 min	oxy-QPS destruction **
**4** + DMSO	5 min	-
**5** + DMSO	>12 h	-
**7** + DMSO	>8 h	oxy-QPS crystallization
**8** + DMSO	>8 h	-
**9** + DMSO	>4 h	-
**10** + DMSO	>8 h	oxy-QPS destruction
**11** + DMSO	>4 h	-
**12** + DMSO	>8 h	oxy-QPS destruction
DMSO	>17 h	-

* 80 °C, 800 rpm; ** according NMR analysis.

**Table 5:** The time of cellulose dissolution at varied temperatures.

The Mixture (1:1)	The Temperature of the Dissolution Process, °C		
	60	70	80
**1** + DMSO	>8 h	5 min	5 min
**3** + DMSO	>8 h	5 h	90 min
**4** + DMSO	oxy-QPS crystallization	5 min	5 min

**Table 6:** Antimicrobial activity * of oxy-QPSs.

oxy-QPSs	MIC—Minimal Inhibition Concentration, µg/mL				
	*Sa*	*Bc*	*Ef*	*Ec*	*Ca*
**1**	250 ± 20	>250	>250	>250	>250
**7**	62.5 ± 5.3	>250	>250	>250	>250
**9**	250 ± 19	>250	>250	>250	>250
**10**	62.5 ± 5.2	>250	>250	>250	>250
**11**	250 ± 21	>250	>250	>250	>250
**12**	250 ± 18	>250	>250	>250	>250
Chloramphenicol	62.5 ± 5.5	62.5 ± 5.3	125 ± 11	-	-
**MBC** and **MFC**—bactericide and fungicide activity, µg/mL					
**1**	250 ± 21	>250	>250	>250	>250
**7**	250 ± 19	>250	>250	>250	>250
**9**	250 ± 19	>250	>250	>250	>250
**10**	62.5 ± 6.4	>250	>250	>250	>250
**11**	250 ± 22	>250	>250	>250	>250
**12**	250 ± 20	>250	>250	>250	>250

* The experiment was repeated three times.

**Table 7:** Cytotoxic activity * of oxy-QPSs.

oxy-QPSs	IC_50_, µg/mL		Hemolysis HC_50_, µg/mL
	Wi38	Chang Liver	
**1**	206.1	132.7	>500
**4**	238.4	113.4	>500
**5**	230.9	208.9	>500
**7**	276.8	260.8	>500
**8**	295.9	237.8	>500
**9**	144.3	407.8	>500
**10**	470.8	351.9	>500
**11**	208.4	223.8	>500
**12**	207.5	203.1	>500

* The experiment was repeated three times.

## Data Availability

All data used during the study appear in the submitted article.

## Supplementary Materials

The following supporting information can be downloaded at https://www.mdpi.com/article/10.3390/polym15204097/s1. Figure S1: The ^1^H NMR (300 MHz, CDCl_3_) of tri-*tert*-butyl(methoxymethyl)phosphonium chloride (**1**); Figure S2: The ^13^C{^1^H} NMR (75 MHz, CDCl_3_) of tri-*tert*-butyl(methoxymethyl)phosphonium chloride (**1**); Figure S3: The ^31^P{^1^H} NMR (121.5 MHz, CDCl_3_) of tri-*tert*-butyl(methoxymethyl)phosphonium chloride (**1**); Figure S4: The ^1^H NMR (300 MHz, CDCl_3_) of tri-*tert*-butyl(methoxymethyl)phosphonium tetrafluoroborate (**2**); Figure S5: The ^13^C{^1^H} NMR (75 MHz, CDCl_3_) of tri-*tert*-butyl(methoxymethyl)phosphonium tetrafluoroborate (**2**); Figure S6: The ^31^P{^1^H} NMR (121.5 MHz, CDCl_3_) of tri-*tert*-butyl(methoxymethyl)phosphonium tetrafluoroborate (**2**); Figure S7: The ^1^H NMR (300 MHz, CDCl_3_) of tri-*tert*-butyl(2-ethoxyethyl)phosphonium bromide (**3**); Figure S8: The ^13^C{^1^H} NMR (75 MHz, CDCl_3_) of tri-*tert*-butyl(2-ethoxyethyl)phosphonium bromide (**3**); Figure S9: The ^31^P{^1^H} NMR (121.5 MHz, CDCl_3_) of tri-*tert*-butyl(2-ethoxyethyl)phosphonium bromide (**3**); Figure S10: The ^1^H NMR (300 MHz, CDCl_3_) of tri-*tert*-butyl((2-methoxyethoxy)methyl)phosphonium chloride (**4**); Figure S11: The ^13^C{^1^H} NMR (75 MHz, CDCl_3_) of tri-*tert*-butyl((2-methoxyethoxy)methyl)phosphonium chloride (**4**); Figure S12: The ^31^P{^1^H} NMR (121.5 MHz, CDCl_3_) of tri-*tert*-butyl((2-methoxyethoxy)methyl)phosphonium chloride (**4**); Figure S13: The ^1^H NMR (300 MHz, CDCl_3_) of tri-*tert*-butyl((2-methoxyethoxy)methyl)phosphonium tetrafluoroborate (**5**); Figure S14: The ^13^C{^1^H} NMR (75 MHz, CDCl_3_) of tri-*tert*-butyl((2-methoxyethoxy)methyl)phosphonium tetrafluoroborate (**5**); Figure S15: The ^31^P{^1^H} NMR (121.5 MHz, CDCl_3_) of tri-*tert*-butyl((2-methoxyethoxy)methyl)phosphonium tetrafluoroborate (**5**); Figure S16: The ^1^H NMR (300 MHz, CDCl_3_) tri-*tert*-butyl((2-methoxyethoxy)methyl)phosphonium hexafluorophosphate (**6**); Figure S17: The ^13^C{^1^H} NMR (75 MHz, CDCl_3_) of tri-*tert*-butyl((2-methoxyethoxy)methyl)phosphonium hexafluorophosphate (**6**); Figure S18: The ^31^P{^1^H} NMR (121.5 MHz, CDCl_3_) of tri-*tert*-butyl((2-methoxyethoxy)methyl)phosphonium hexafluorophosphate (**6**); Figure S19: The ^1^H NMR (300 MHz, CDCl_3_) tri-*tert*-butyl(2-carboxyethyl)phosphonium bromide (**7**); Figure S20: The ^13^C{^1^H} NMR (75 MHz, CDCl_3_) of tri-*tert*-butyl(2-carboxyethyl)phosphonium bromide (**7**); Figure S21: The ^31^P{^1^H} NMR (121.5 MHz, CDCl_3_) of tri-*tert*-butyl(2-carboxyethyl)phosphonium bromide (**7**); Figure S22: The ^1^H NMR (300 MHz, CDCl_3_) tri-*tert*-butyl(5-carboxypentyl)phosphonium bromide (**8**); Figure S23: The ^13^C{^1^H} NMR (75 MHz, CDCl_3_) of tri-*tert*-butyl(5-carboxypentyl)phosphonium bromide (**8**); Figure S24: The ^31^P{^1^H} NMR (121.5 MHz, CDCl_3_) of tri-*tert*-butyl(5-carboxypentyl)phosphonium bromide (**8**); Figure S25: The ^1^H NMR (300 MHz, CDCl_3_) tri-*tert*-butyl(2-methoxy-2-oxoethyl)phosphonium bromide (**9**); Figure S26: The ^13^C{^1^H} NMR (75 MHz, CDCl_3_) of tri-*tert*-butyl(2-methoxy-2-oxoethyl)phosphonium bromide (**9**); Figure S27: The ^31^P{^1^H} NMR (121.5 MHz, CDCl_3_) of tri-*tert*-butyl(2-methoxy-2-oxoethyl)phosphonium bromide (**9**); Figure S28: The ^1^H NMR (300 MHz, CDCl_3_) tri-*tert*-butyl(2-ethoxy-2-oxoethyl)phosphonium bromide (**10**); Figure S29: The ^13^C{^1^H} NMR (75 MHz, CDCl_3_) of tri-*tert*-butyl(2-ethoxy-2-oxoethyl)phosphonium bromide (**10**); Figure S30: The ^31^P{^1^H} NMR (121.5 MHz, CDCl_3_) of tri-*tert*-butyl(2-ethoxy-2-oxoethyl)phosphonium bromide (**10**); Figure S3: The ^1^H NMR (300 MHz, CDCl_3_) tri-*tert*-butyl(2-*iso*-propoxy-2-oxoethyl)phosphonium bromide (**11**); Figure S32: The ^13^C{^1^H} NMR (75 MHz, CDCl_3_) of tri-*tert*-butyl(2-*iso*-propoxy-2-oxoethyl)phosphonium bromide (**11**); Figure S33: The ^31^P{^1^H} NMR (121.5 MHz, CDCl_3_) of tri-*tert*-butyl(2-*iso*-propoxy-2-oxoethyl)phosphonium bromide (**11**); Figure S34: The ^1^H NMR (300 MHz, CDCl_3_) tri-*tert*-butyl(2-butoxy-2-oxoethyl)phosphonium bromide (**12**); Figure S35: The ^13^C{^1^H} NMR (75 MHz, CDCl_3_) of tri-*tert*-butyl(2-butoxy-2-oxoethyl)phosphonium bromide (**12**); Figure S36: The ^31^P{^1^H} NMR (121.5 MHz, CDCl_3_) of tri-*tert*-butyl(2-butoxy-2-oxoethyl)phosphonium bromide (**12**); Figure S37: The IR spectra of tri-*tert*-butyl(methoxymethyl)phosphonium chloride (**1**); Figure S38: The IR spectra of tri-*tert*-butyl(methoxymethyl)phosphonium tetrafluoroborate (**2**); Figure S39: The IR spectra of tri-*tert*-butyl(2-ethoxyethyl)phosphonium bromide (**3**); Figure S40: The IR spectra of tri-*tert*-butyl((2-methoxyethoxy)methyl)phosphonium chloride (**4**); Figure S41: The IR spectra of tri-*tert*-butyl((2-methoxyethoxy)methyl)phosphonium tetrafluoroborate (**5**); Figure S42: The IR spectra of tri-*tert*-butyl((2-methoxyethoxy)methyl)phosphonium hexafluorophosphate (**6**); Figure S43: The IR spectra of tri-*tert*-butyl(2-carboxyethyl)phosphonium bromide (**7**); Figure S44: The IR spectra of tri-*tert*-butyl(5-carboxypentyl)phosphonium bromide (**8**); Figure S45: The IR spectra of tri-*tert*-butyl(2-methoxy-2-oxoethyl)phosphonium bromide (**9**); Figure S46: The IR spectra of tri-*tert*-butyl(2-ethoxy-2-oxoethyl)phosphonium bromide (**10**); Figure S47: The IR spectra of tri-*tert*-butyl(2-*iso*-propoxy-2-oxoethyl)phosphonium bromide (**11**); Figure S48: The IR spectra of tri-tert-butyl(2-butoxy-2-oxoethyl)phosphonium bromide (**12**); Figure S49: TG-DSC curves for **1** (tri-*tert*-butyl(methoxymethyl)phosphonium chloride); Figure S50: TG-DSC curves for **2** (tri-*tert*-butyl(methoxymethyl)phosphonium tetrafluoroborate); Figure S51: TG-DSC curves for **3** (tri-*tert*-butyl(2-ethoxyethyl)phosphonium bromide); Figure S52: TG-DSC curves for **4** (tri-*tert*-butyl(MEM)phosphonium chloride); Figure S53: TG-DSC curves for **5** (tri-*tert*-butyl(MEM)phosphonium tetrafluoroborate); Figure S54: TG-DSC curves for **6** (tri-*tert*-butyl(MEM)phosphonium hexafluorophosphate); Figure S55: TG-DSC curves for **7** (tri-*tert*-butyl(2-carboxyethyl)phosphonium bromide); Figure S56: TG-DSC curves for **8** (tri-*tert*-butyl(5-carboxypentyl)phosphonium bromide); Figure S57: TG-DSC curves for **9** (tri-*tert*-butyl(2-methoxy-2-oxoethyl)phosphonium bromide); Figure S58: TG-DSC curves for **10** (tri-*tert*-butyl(2-ethoxy-2-oxoethyl)phosphonium bromide); Figure S59: TG-DSC curves for **11** (tri-*tert*-butyl(2-*iso*-propoxy-2-oxoethyl)phosphonium bromide); Figure S60: TG-DSC curves for **12** (tri-*tert*-butyl(2-butoxy-2-oxoethyl)phosphonium bromide); Figure S61: Molecular structure of **2** in crystal and fragment of the crystal packing; Figure S62: Molecular structure of **4** in crystal and fragment of the crystal packing; Figure S63: Molecular structure of **6** in crystal and fragment of the crystal packing; Figure S64: Molecular structure of **7** in crystal and fragment of the crystal packing; Figure S65: Molecular structure of **8** in crystal and fragment of the crystal packing; Figure S66: Molecular structure of **9** in crystal and fragment of the crystal packing; Figure S67: Molecular structure of **10** in crystal and fragment of the crystal packing; Figure S68: Molecular structure of **11** in crystal and fragment of the crystal packing; Table S1: Selected bond length (Å) and angle (°) in the crystals for compounds **2**; Table S2: Selected bond length (Å) and angle (°) in the crystals for compounds **4**; Table S3. Selected bond length (Å) and angle (°) in the crystals for compounds **6**; Table S4: Selected bond length (Å) and angle (°) in the crystals for compounds **7**; Table S5: Selected bond length (Å) and angle (°) in the crystals for compounds **8**; Table S6: Selected bond length (Å) and angle (°) in the crystals for compounds **9**; Table S7: Selected bond length (Å) and angle (°) in the crystals for compounds **10**; Table S8: Selected bond length (Å) and angle (°) in the crystals for compounds **11**; Table S9: Activity of oxy-QPS against non-pathogenic microbes; Table S10: Ecotoxicity of oxy-QPS to *D. magna*.

## References

[1] Welton T. (2018). Ionic liquids: A brief history. Biophys. Rev..

[2] Esperança J., Canongia Lopes J.N., Tariq M., Santos L.M., Magee J.W., Rebelo L.P.N. (2010). Volatility of Aprotic Ionic Liquids A Review. J. Chem. Eng. Data.

[3] Seitkalieva M.M., Samoylenko D.E., Lotsman K.A., Rodygin K.S., Ananikov V.P. (2021). Metal nanoparticles in ionic liquids: Synthesis and catalytic applications. Coord. Chem. Rev..

[4] Egorova K.S., Gordeev E.G., Ananikov V.P. (2017). Biological activity of ionic liquids and their application in pharmaceutics and medicine. Chem. Rev..

[5] Saverina E.A., Frolov N.A., Kamanina O.A., Arlyapov V.A., Vereshchagin A.N., Ananikov V.P. (2023). From Antibacterial to Antibiofilm Targeting: An Emerging Paradigm Shift in the Development of Quaternary Ammonium Compounds (QACs). ACS Infect. Dis..

[6] Banerjee A., Aremu B.R., Dehghandokht S., Salama R., Zhou H., Lackie S., Seifi M., Kennepohl P., Trant J. (2023). Lethal weapon IL: A nano-copper/tetraalkylphosphonium ionic liquid composite material with potent antibacterial activity. RSC Sustain..

[7] Francis C.F., Kyratzis I.L., Best A.S. (2020). Lithium-ion battery separators for ionic-liquid electrolytes: A review. Adv. Mater..

[8] Zhang S., Zhang Q., Zhang Y., Chen Z., Watanabe M., Deng Y. (2016). Beyond solvents and electrolytes: Ionic liquids based advanced functional materials. Prog. Mater. Sci..

[9] Giernoth R. (2010). Task-specific ionic liquids. Angew. Chem. Int. Ed..

[10] Tang S., Baker G.A., Zhao H. (2012). Ether-and alcohol-functionalized task-specific ionic liquids: Attractive properties and applications. Chem. Soc. Rev..

[11] Yang J., Chen P., Pan Y., Liang Y. (2022). Fixation of Carbon Dioxide with Functionalized Ionic Liquids. Asian J. Org. Chem..

[12] Wu D., Cai P., Zhao X., Kong Y., Pan Y. (2018). Recent progress of task-specific ionic liquids in chiral resolution and extraction of biological samples and metal ions. J. Sep. Sci..

[13] Rennie A.J., Sanchez-Ramirez N.D., Torresi R.M., Hall P.J. (2013). Ether-bond-containing ionic liquids as supercapacitor electrolytes. J. Phys. Chem. Lett..

[14] Rauber D., Philippi F., Becker J., Zapp J., Morgenstern B., Kuttich B., Kraus T., Hempelmann R., Hunt P., Welton T. (2023). Anion and ether group influence in protic guanidinium ionic liquids. Phys. Chem. Chem. Phys..

[15] Moiseev D.V., James B.R. (2020). Tetrakis(hydroxymethyl)phosphonium salts: Their properties, hazards and toxicities. Phosphorus Sulfur Silicon Relat. Elem..

[16] Itoh T., Kude K., Hayase S., Kawatsura M. (2007). Design of ionic liquids as a medium for the Grignard reaction. Tetrahedron Lett..

[17] Philippi F., Rauber D., Zapp J., Präsang C., Scheschkewitz D., Hempelmann R. (2019). Multiple Ether-Functionalized Phosphonium Ionic Liquids as Highly Fluid Electrolytes. ChemPhysChem.

[18] Abe Y., Yoshiyama K., Yagi Y., Hayase S., Kawatsura M., Itoh T. (2010). A rational design of phosphonium salt type ionic liquids for ionic liquid coated-lipase catalyzed reaction. Green Chem..

[19] Atefi F., Garcia M.T., Singer R.D., Scammells P.J. (2009). Phosphonium ionic liquids: Design, synthesis and evaluation of biodegradability. Green Chem..

[20] Cuthbert T.J., Guterman R., Ragogna P.J., Gillies E.R. (2015). Contact active antibacterial phosphonium coatings cured with UV light. J. Mater. Chem. B.

[21] Cuthbert T.J., Hisey B., Harrison T.D., Trant J.F., Gillies E.R., Ragogna P.J. (2018). Surprising antibacterial activity and selectivity of hydrophilic polyphosphoniums featuring sugar and hydroxy substituents. Angew. Chem. Int. Ed..

[22] Glińska K., Gitalt J., Torrens E., Plechkova N., Bengoa C. (2021). Extraction of cellulose from corn stover using designed ionic liquids with improved reusing capabilities. Process Saf. Environ. Prot..

[23] Tao J., Kishimoto T., Hamada M., Nakajima N. (2016). Novel cellulose pretreatment solvent: Phosphonium-based amino acid ionic liquid/cosolvent for enhanced enzymatic hydrolysis. Holzforschung.

[24] Mai N.L., Koo Y.-M. (2016). Computer-Aided Design of Ionic Liquids for High Cellulose Dissolution. ACS Sustain. Chem. Eng..

[25] Nguyen H.V.T., Faheem A.B., Kim J., Lee J., Jin Q., Koo B., Kim W., Lee K.-K. (2023). Size Effect of Electrolyte Ions on the Electric Double-Layer Structure and Supercapacitive Behavior. ACS Appl. Energy Mater..

[26] Phromphithak S., Onsree T., Tippayawong N. (2021). Machine learning prediction of cellulose-rich materials from biomass pretreatment with ionic liquid solvents. Bioresour. Technol..

[27] Arkhipova D., Ermolaev V., Miluykov V., Nigmatullina L., Sinyashin O. (2015). Functionalized Phosphonium Ionic Liquids: Synthesis and Application. Phosphorus Sulfur Silicon Relat. Elem..

[28] Arkhipova D.M., Ermolaev V.V., Miluykov V.A., Gubaidullin A.T., Islamov D.R., Kataeva O.N., Ananikov V.P. (2020). Sterically Hindered Phosphonium Salts: Structure, Properties and Palladium Nanoparticle Stabilization. Nanomaterials.

[29] Arkhipova D.M., Ermolaev V.V., Miluykov V.A., Valeeva F.G., Gaynanova G.A., Zakharova L.Y., Minyaev M.E., Ananikov V.P. (2021). Tri-*tert*-butyl(*n*-alkyl)phosphonium Ionic Liquids: Structure, Properties and Application as Hybrid Catalyst Nanomaterials. Sustainability.

[30] CrysAlisPro (2021). Rigaku Oxford Diffraction.

[31] Sheldrick G.M. (2015). SHELXT—Integrated space-group and crystal-structure determination. Acta Cryst..

[32] Sheldrick G.M. (2015). Crystal structure refinement with SHELXL. Acta Cryst..

[33] Dolomanov O.V., Bourhis L.J., Gildea R.J., Howard J.A.K., Puschmann H. (2009). OLEX2: A complete structure solution, refinement and analysis program. J. Appl. Cryst..

[34] National Committee for Clinical Laboratory Standards (2000). Methods for Dilution Antimicrobial Susceptibility. Tests for Bacteria that Grow Aerobically, Approved Standard, M7-A5.

[35] National Committee for Clinical Laboratory Standards (1998). Reference Method for Broth Dilution Antifungal Susceptibility Testing of Conidium-Forming Filamentous Fungi: Proposed Standard, M38-P.

[36] Voloshina A.D., Sapunova A.S., Kulik N.V., Belenok M.G., Strobykina I.Y., Lyubina A., Gumerova S.K., Kataev V.E. (2021). Antimicrobia and cytotoxic effects of ammonium derivatives of diterpenoids steviol and isosteviol. Bioorg. Med. Chem..

[37] AAT Bioquest, Inc. https://www.aatbio.com/tools/ic50-calculator.

[38] (2012). Water Quality. Determination of the Inhibition of the Mobility of *Daphnia magna* Straus (Cladocera, Crustacea). Acute Toxicity Test, IDT.

[39] (2009). Aquatic Ecotoxicology—Acute Toxicity—Test with Daphnia spp. (Cladocera, Crustacea).

[40] OECD (2004). Test No. 202: Daphnia sp. Acute Immobilization Test, OECD Guidelines for the Testing of Chemicals, Section 2.

[41] Regulation (EC) No 1272/2008 of the European Parliament and of the Council of 16 December 2008 on Classification, Labelling and Packaging of Substances and Mixtures, Amending and Repealing Directives 67/548/EEC and 1999/45/EC, and Amending Regulation (EC) No 1907/2006. https://eur-lex.europa.eu/legal-content/EN/TXT/?uri=celex%3A32008R1272.

[42] Jin Y., Fang S., Chai M., Yang L., Hirano S.I. (2012). Ether-functionalized trialkylimidazolium ionic liquids: Synthesis, characterization, and properties. Ind. Eng. Chem. Res..

[43] Morrissey S., Pegot B., Coleman D., Garcia M.T., Ferguson D., Quilty B., Gathergood N. (2009). Biodegradable, non-bactericidal oxygen-functionalised imidazolium esters: A step towards ‘greener’ ionic liquids. Green Chem..

[44] Mital D.K., Nancarrow P., Ibrahim T.H., Jabbar N.A., Khamis M.I. (2022). Ionic Liquid Melting Points: Structure–Property Analysis and New Hybrid Group Contribution Model. Ind. Eng. Chem. Res..

[45] Adamová G., Gardas R.L., Nieuwenhuyzen M., Puga A.V., Rebelo L.P.N., Robertson A.J., Seddon K.R. (2012). Alkyltributylphosphonium chloride ionic liquids: Synthesis, physicochemical properties and crystal structure. Dalton Trans..

[46] Fei Z., Ang W.H., Zhao D., Scopelliti R., Zvereva E.E., Katsyuba S.A., Dyson P.J. (2007). Revisiting Ether-Derivatized Imidazolium-Based Ionic Liquids. J. Phys. Chem. B.

[47] Xuan X.-P., Chang L.-L., Zhang H., Wang N., Zhao Y. (2014). Hydrogen bonds in the crystal structure of hydrophobic and hydrophilic COOH-functionalized imidazolium ionic liquids. CrystEngComm.

[48] Macrae C.F., Sovago I., Cottrell S.J., Galek P.T.A., McCabe P., Pidcock E., Platings M., Shields G.P., Stevens J.S., Towler M. (2020). Mercury 4.0: From visualization to analysis, design and prediction. J. Appl. Cryst..

[49] Tursi A. (2019). A review on biomass: Importance, chemistry, classification, and conversion. Biofuel Res. J..

[50] Li T., Chen C., Brozena A.H., Zhu J.Y., Xu L., Driemeier C., Dai J., Rojas O.J., Isogai A., Wågberg L. (2021). Developing fibrillated cellulose as a sustainable technological material. Nature.

[51] Kucherov F.A., Gordeev E.G., Kashin A.S., Ananikov V.P. (2017). Three-dimensional printing with biomass-derived PEF for carbon-neutral manufacturing. Angew. Chem. Int. Ed. Engl..

[52] Seitkalieva M.M., Vavina A.V., Posvyatenko A.V., Egorova K.S., Kashin A.S., Gordeev E.G., Strukova E.N., Romashov L.V., Ananikov V.P. (2021). Biomass-derived ionic liquids based on a 5-HMF platform chemical: Synthesis, characterization, biological activity, and tunable interactions at the molecular level. ACS Sustain. Chem. Eng..

[53] Acharya S., Liyanage S., Parajuli P., Rumi S.S., Shamshina J.L., Abidi N. (2021). Utilization of Cellulose to Its Full Potential: A Review on Cellulose Dissolution, Regeneration, and Applications. Polymers.

[54] Hernández-Beltrán J.U., Hernández-De Lira I.O., Cruz-Santos M.M., Saucedo-Luevanos A., Hernández-Terán F., Balagurusamy N. (2019). Insight into Pretreatment Methods of Lignocellulosic Biomass to Increase Biogas Yield: Current State, Challenges, and Opportunities. Appl. Sci..

[55] Khoo K.S., Tan X., Ooi C.W., Chew K.W., Leong W.H., Chai Y.H., Ho S.-H., Show P.L. (2021). How does ionic liquid play a role in sustainability of biomass processing?. J. Clean. Prod..

[56] Tadesse H., Luque R. (2011). Advances on biomass pretreatment using ionic liquids: An overview. Energy Environ. Sci..

[57] Fan Y., Picchioni F. (2020). Modification of starch: A review on the application of “green” solvents and controlled functionalization. Carbohydr. Polym..

[58] Zhang H., Xu Y., Li Y., Lu Z., Cao S., Fan M., Huang L., Chen L. (2017). Facile Cellulose Dissolution and Characterization in the Newly Synthesized 1,3-Diallyl-2-ethylimidazolium Acetate Ionic Liquid. Polymers.

[59] Abushammala H., Mao J. (2020). A Review on the Partial and Complete Dissolution and Fractionation of Wood and Lignocelluloses Using Imidazolium Ionic Liquids. Polymers.

[60] Acharya S., Hu Y., Abidi N. (2021). Cellulose Dissolution in Ionic Liquid under Mild Conditions: Effect of Hydrolysis and Temperature. Fibers.

[61] Burns F.P., Themens P.A., Ghandi K. (2014). Assessment of phosphonium ionic liquid-dimethylformamide mixtures for dissolution of cellulose. Compos. Interfaces.

[62] Halder P., Kundu S., Patel S., Setiawan A., Atkin R., Parthasarthy R., Paz-Ferreiro J., Surapaneni A., Shah K. (2019). Progress on the pre-treatment of lignocellulosic biomass employing ionic liquids. Renew. Sustain. Energy Rev..

[63] El Seoud O.A., Kostag M., Jedvert K., Malek N.I. (2019). Cellulose in Ionic Liquids and Alkaline Solutions: Advances in the Mechanisms of Biopolymer Dissolution and Regeneration. Polymers.

[64] Andanson J.-M., Bordes E., Devémy J., Leroux F., Pádua A.A.H., Costa Gomes M.F. (2014). Understanding the role of co-solvents in the dissolution of cellulose in ionic liquids. Green Chem..

[65] Wang S., Zhao W., Lee T.S., Singer S.W., Simmons B.A., Singh S., Yuan Q., Cheng G. (2018). Dimethyl sulfoxide assisted ionic liquid pretreatment of switchgrass for isoprenol production. ACS Sustain. Chem. Eng..

[66] Andanson J.-M., Padua A.A.H., Costa Gomes M.F. (2015). Thermodynamics of cellulose dissolution in an imidazolium acetate ionic liquid. Chem. Commun..

[67] Hunt P.A., Ashworth C.R., Matthews R.P. (2015). Hydrogen bonding in ionic liquids. Chem. Soc. Rev..

[68] Ammer J., Nolte C., Karaghiosoff K., Thallmair S., Mayer P., de Vivie-Riedle R., Mayr H. (2013). Ion-Pairing of Phosphonium Salts in Solution: C-H⋅⋅⋅Halogen and C-H⋅⋅⋅π Hydrogen Bond. Chem. Eur. J..

[69] Ermolaev V.V., Arkhipova D.M., Miluykov V.A., Lyubina A.P., Amerhanova S.K., Kulik N.V., Ananikov V.P. (2021). Sterically hindered quaternary phosphonium salts (QPSs): Antimicrobial activity and hemolytic and cytotoxic properties. Int. J. Mol. Sci..

[70] Bedard J., Caschera A., Foucher D.A. (2021). Access to thermally robust and abrasion resistant antimicrobial plastics: Synthesis of UV-curable phosphonium small molecule coatings and extrudable additives. RSC Adv..

[71] Cuthbert T.J., Harrison T.D., Ragogna P.J., Gillies E.R. (2016). Synthesis, properties, and antibacterial activity of polyphosphonium semi-interpenetrating networks. J. Mater. Chem. B.

[72] Schifano J.M., Edifor R., Sharp J.D., Ouyang M., Konkimalla A., Husson R.N., Woychik N.A. (2013). Mycobacterial toxin MazF-mt6 inhibits translation through cleavage of 23S rRNA at the ribosomal A site. Proc. Natl. Acad. Sci. USA.

[73] Jardetzky O. (1963). Studies on the mechanism of action of chloramphenicol. I. The conformation of chlioramphenicol in solution. J. Biol. Chem..

[74] Wolfe A.D., Hahn F.E. (1965). Mode of action of chloramphenicol IX. Effects of chloramphenicol upon a ribosomal amino acid polymerization system and its binding to bacterial ribosome. Biochim. Biophys. Acta Nucleic Acids Protein Synth..

[75] Hahn F.E., Wisseman C.L., Hopps H.E. (1955). Mode of action of chloramphenicol. III. Action of chloramphenicol on bacterial energy metabolism. J. Bacteriol..

[76] Yu C., Simmons B.A., Singer S.W., Thelen M.P., VanderGheynst J.S. (2016). Ionic liquid-tolerant microorganisms and microbial communities for lignocellulose conversion to bioproducts. Appl. Microbiol. Biotechnol..

[77] Zhang J., Zou D., Singh S., Cheng G. (2021). Recent developments in ionic liquid pretreatment of lignocellulosic biomass for enhanced bioconversion. Sustain. Energy Fuels.

[78] Kumari P., Pillai V.V., Benedetto A. (2020). Mechanisms of action of ionic liquids on living cells: The state of the art. Biophys. Rev..

[79] Tramer F., Da Ros T., Passamonti S., Reineke J. (2012). Screening of Fullerene Toxicity by Hemolysis Assay. Nanotoxicity. Methods in Molecular Biology.

