# Supplementary material for: Oxygen-Containing Quaternary Phosphonium Salts (oxy-QPSs): Synthesis, Properties, and Cellulose Dissolution
Source: Polymers (Basel). 2023 Oct 16;15(20):4097. doi: 10.3390/polym15204097 (PMC10611013; doi:10.3390/polym15204097)
Supplement: Supplementary file 1 [file polymers-15-04097-s001.zip › polymers-2607940-supplementary.pdf]

Supplementary Materials

Oxygen-containing quaternary phosphonium salts (oxy-QPS): synthesis, properties, and cellulose dissolution

Daria M. Arkhipova[1]*, Vadim V. Ermolaev[2], Gulnaz R. Baembitova[2], Aida I. Samigullina[1], Anna P. Lyubina[2], and Alexandra D. Voloshina[2]

[1] N.D. Zelinsky Institute of Organic Chemistry, Russian Academy of Sciences, Moscow 119991, Russia; arkhipova_daria@ioc.ac.ru

[2] Arbuzov Institute of Organic and Physical Chemistry, FRC Kazan Scientific Center of Russian Academy of Sciences, Kazan 420088, Russia

** Correspondence: arkhipova_daria@ioc.ac.ru*

The synthesis of oxy-QPSs

Tri-*tert*-butyl(methoxymethyl)phosphonium chloride (**1**)

Tri-*tert*-butylphosphine (0.83 g, 4.1 mmol) and chloromethyl methyl ether (0.31 mL, 4.1 mmol) were stirred at 50 °C for 6 hours. The reaction mixture was cooled; white solid was washed with diethyl ether. White crystalline powder, 0.36 g (31%), melting point 144 °C.

^{1}H NMR (300 MHz, $CDCl_3$) δ 5.11 (d, $^2J_{PH}$ = 4.1 Hz, 2H, P-CH_2), 3.74 (s, 3H, O-CH_3), 1.69 (d, $^3J_{PH}$ = 14.2 Hz, 27H, (C(CH_3)$_3$)$_3$). ^{13}C NMR (75 MHz, $CDCl_3$) δ 61.9 (d, $^1J_{CP}$ = 53.0 Hz, P-CH_2), 61.9 (d, $^3J_{CP}$ = 12.9 Hz, O-CH_3), 38.8 (d, $^1J_{CP}$ = 27.9 Hz, (*C*(CH_3)$_3$)$_3$), 29.7 (s, C(*C*H_3)$_3$)$_3$). ^{31}P NMR (121.5 MHz, $CDCl_3$) δ 44.4 (s). HRMS (ESI) Calcd. for $[C_{14}H_{32}PO]^+$ $[M]^+$: 247.2185, Found: 247.2184.

Tri-*tert*-butyl(methoxymethyl)phosphonium tetrafluoroborate (**2**)

Tri-*tert*-butyl(methoxymethyl)phosphonium chloride (1.63 g, 5.8 mmol) was dissolved in distilled water (3 ml) and then the solution of sodium tetrafluoroborate (0.95 g, 8.7 mmol) in 2 ml water was added. Immediately a white precipitate of tri-*tert*-butyl(methoxymethyl)phosphonium tetrafluoroborate precipitates. White solid was isolated by filtration, dissolved in 5 ml methylene chloride and dried over anhydrous magnesium sulfate overnight. The solvent was removed under reduced pressure. White crystalline powder, 1.78 g (92%), melting point 142 °C.

^{1}H NMR (300 MHz, $CDCl_3$) δ 4.57 (d, $^2J_{PH}$ = 4.2 Hz, 2H, P-CH_2), 3.62 (s, 3H, O-CH_3), 1.66 (d, $^3J_{PH}$ = 14.1 Hz, 27H, (C(CH_3)$_3$)$_3$). ^{13}C NMR (75 MHz, $CDCl_3$) δ 61.6 (d, $^3J_{CP}$ = 13.0 Hz, O-CH_3), 61.3 (d, $^1J_{CP}$ = 54.4 Hz, P-CH_2), 38.9 (d, $^1J_{CP}$ = 27.6 Hz, (*C*(CH_3)$_3$)$_3$), 29.5 (s, C(*C*H_3)$_3$)$_3$). ^{31}P NMR (121.5 MHz, $CDCl_3$) δ 44.0 (s). HRMS (ESI) Calcd. for $[C_{14}H_{32}PO]^+$ $[M]^+$: 247.2185, Found: 247.2192.

Tri-*tert*-butyl(2-ethoxyethyl)phosphonium bromide (**3**)

Tri-*tert*-butylphosphine (1.82 g, 9.0 mmol) and (2-bromoethyl) ethyl ether (0.99 mL, 9.0 mmol) were stirred at 90 °C for 6 hours. The reaction mixture was cooled; white solid was washed with diethyl ether. White amorphous powder, 2.91 g (83%), melting point 109 °C.

^{1}H NMR (300 MHz, $CDCl_3$) δ 3.96 (dt, $^3J_{PH}$ = 14.1 Hz, $^3J_{HH}$ = 6.0 Hz, 2H, P-CH_2-CH_2), 3,43 (q, $^3J_{HH}$ = 7.0 Hz, 2H, O-CH_2-CH_3), 2.95 (dt, $^2J_{PH}$ = 11.9 Hz, $^3J_{HH}$ = 6.1 Hz, 2H, P-CH_2), 1.54 (d, $^3J_{PH}$ = 14.1 Hz, 27H, P(C(CH_3)$_3$)$_3$), 1.04 (t, $^3J_{HH}$ = 7.0 Hz, 3H, CH_2-CH_3). ^{13}C NMR (75 MHz, $CDCl_3$) δ 66.7 (s, O-*C*H_2-CH_3), 64.2 (d, $^2J_{PC}$ = 5.6 Гц, P-CH_2-*C*H_2), 39.4 (d, $^1J_{PC}$ = 29.1 Гц, P(*C*(CH_3)$_3$)$_3$), 30.1 (s, P(C(*C*H_3)$_3$)$_3$), 20.2 (d, $^1J_{PC}$ = 35.0 Гц, P-CH_2), 15.0 (s, CH_2-*C*H_3). ^{31}P NMR (121.5 MHz, $CDCl_3$) δ 51.6 (s). HRMS (ESI) Calcd. for $[C_{16}H_{36}PO]^+$ $[M]^+$: 275.2498, Found: 275.2507.

Tri-*tert*-butyl((2-methoxyethoxy)methyl)phosphonium chloride (**4**)

Tri-*tert*-butylphosphine (1.13 g, 5.5 mmol) and 2-methoxyethoxymethyl chloride (0.66 mL, 5.5 mmol) were stirred at 100 °C for 6 hours. The reaction mixture was cooled; white solid was washed with diethyl ether. White crystalline powder, 1.63 g (91%), melting point 178 °C.

^{1}H NMR (300 MHz, $CDCl_3$) δ 5.17 (d, $^2J_{PH}$ = 4.2 Hz, 2H, P-CH_2), 4.08 (m, 2H, O-CH_2-CH_2-O), 3.61 (m, 2H, O-CH_2-CH_2-O), 3.34 (s, 3H, O-CH_3), 1.68 (d, $^3J_{PH}$ = 14.1 Hz, 27H, (C(CH_3)$_3$)$_3$). ^{13}C NMR (75 MHz, $CDCl_3$) δ 72.7 (d, $^3J_{CP}$ = 11.7 Hz, O-*C*H_2-CH_2-O), 71.3 (s, O-CH_2-*C*H_2-O), 60.5 (d, $^1J_{CP}$ = 52.8 Hz, P-CH_2), 58.6 (s, O-CH_3), 38.8 (d, $^1J_{CP}$ = 27.6 Hz, (*C*(CH_3)$_3$)$_3$), 29.7 (s, C(*C*H_3)$_3$)$_3$). ^{31}P NMR (121.5 MHz, $CDCl_3$) δ 44.7 (s). HRMS (ESI) Calcd. for $[C_{16}H_{36}PO_2]^+$ $[M]^+$: 291.2447, Found: 291.2453.

Tri-*tert*-butyl((2-methoxyethoxy)methyl)phosphonium tetrafluoroborate (**5**)

Tri-*tert*-butyl((2-methoxyethoxy)methyl)phosphonium chloride (1.67 g, 5.1 mmol) was dissolved in distilled water (3 ml) and then the solution of sodium tetrafluoroborate (0.84 g, 7.6 mmol) in 2 ml water was added. White flaked precipitation appears after 30 min of the solution stirring at room temperature. White solid was isolated by filtration, dissolved in 5 ml methylene chloride and dried over anhydrous magnesium sulfate overnight. The solvent was removed under reduced pressure to give tri-*tert*-butyl(MEM)phosphonium tetrafluoroborate 1.73 g (89%) as white crystalline powder, melting point 92 °C.

^{1}H NMR (300 MHz, $CDCl_3$) δ 4.62 (d, $^2J_{PH}$ = 4.3 Hz, 2H, P-CH_2), 3.89 (m, 2H, O-CH_2-CH_2-O), 3.58 (m, 2H, O-CH_2-CH_2-O), 3.34 (s, 3H, O-CH_3), 1.66 (d, $^3J_{PH}$ = 14.1 Hz, 27H, ($C(CH_3)_3)_3$). ^{13}C NMR (75 MHz, $CDCl_3$) δ 72.7 (d, $^3J_{CP}$ = 11.4 Hz, O-CH_2-CH_2-O), 71.2 (s, O-CH_2-CH_2-O), 59.8 (d, $^1J_{CP}$ = 54.5 Hz, P-CH_2), 58.6 (s, O-CH_3), 38.8 (d, $^1J_{CP}$ = 27.4 Hz, ($C(CH_3)_3)_3$), 29.5 (s, $C(CH_3)_3)_3$). ^{31}P NMR (121.5 MHz, $CDCl_3$) δ 44.3 (s). HRMS (ESI) Calcd. for $[C_{16}H_{36}PO_2]^+$ $[M]^+$: 291.2447, Found: 291.2457.

Tri-*tert*-butyl((2-methoxyethoxy)methyl)phosphonium hexafluorophosphate (**6**)

Tri-*tert*-butyl((2-methoxyethoxy)methyl)phosphonium chloride (0.5 g, 1.5 mmol) was dissolved in distilled water (1 mL) and then the solution of sodium hexfluorophosphate (0.39 g, 2.3 mmol) in 1 mL water was added. White flaked precipitation appears immediately stirred at room temperature. White solid was isolated by filtration, dissolved in 5 mL methylene chloride and dried over anhydrous magnesium sulfate overnight. The solvent was removed under reduced pressure to give tri-*tert*-butyl((2-methoxyethoxy)methyl)phosphonium hexafluorophosphate 0.56 g (85%) as white crystalline powder, melting point 87 °C.

^{1}H NMR (300 MHz, $CDCl_3$) δ 4.55 (d, $^2J_{PH}$ = 4.4 Hz, 2H, P-CH_2), 3.88 (m, 2H, O-CH_2-CH_2-O), 3.59 (m, 2H, O-CH_2-CH_2-O), 3.36 (s, 3H, O-CH_3), 1.65 (d, $^3J_{PH}$ = 14.2 Hz, 27H, ($C(CH_3)_3)_3$). ^{13}C NMR (75 MHz, $CDCl_3$) δ 72.8 (d, $^3J_{CP}$ = 11.7 Hz, O-CH_2-CH_2-O), 71.2 (s, O-CH_2-CH_2-O), 59.8 (d, $^1J_{CP}$ = 54.1 Hz, P-CH_2), 58.6 (s, O-CH_3), 38.9 (d, $^1J_{CP}$ = 26.2 Hz, ($C(CH_3)_3)_3$), 29.4 (s, $C(CH_3)_3)_3$). ^{31}P NMR (121.5 MHz, $CDCl_3$) δ +44.2 (s), -144.3 (sept, $^1J_{PF}$ = 712.6 Hz, PF_6). HRMS (ESI) Calcd. for $[C_{16}H_{36}PO_2]^+$ $[M]^+$: 291.2447, Found: 291.2452.

Tri-*tert*-butyl(2-carboxyethyl)phosphonium bromide (**7**)

Tri-*tert*-butylphosphine (0.96 g, 4.7 mmol) was dissolved in acetonitrile (1.1 ml) and then 3-bromopropanoic acid (0.72 g, 4.7 mmol) in 5 ml acetonitrile was added. The mixture was stirred at 100 °C for 4 hours. The reaction mixture was cooled; the solvent was removed under reduced pressure. White solid was washed with diethyl ether. White crystalline powder, 1.68 g (80%), melting point 173 °C.

^{1}H NMR (300 MHz, $CDCl_3$) δ 3.26 (dt, $^2J_{PH}$ = 8.1 Hz, $^3J_{HH}$ = 7.2 Hz, 2H, CH_2-CO), 2.75 (m, 2H, P-CH_2), 1.64 (d, $^3J_{PH}$ = 14.1 Hz, 27H, $P(C(CH_3)_3)_3$). ^{13}C NMR (75 MHz, $CDCl_3$) δ 171.8 (d, $^3J_{PC}$ = 12.5 Hz, CO), 39.4 (d, $^1J_{PC}$ = 29.1 Hz, $P(C(CH_3)_3)_3$), 30.0 (d, CH_2-CO), 30.0 (s, $P(C(CH_3)_3)_3$), 13.6 (d, $^1J_{PC}$ = 39.7 Hz, P-CH_2). ^{31}P NMR (121.5 MHz, $CDCl_3$) δ 50.0 (s). HRMS (ESI) Calcd. for $[C_{15}H_{32}PO_2]^+$ $[M]^+$: 275.2133, Found: 275.2133.

Tri-*tert*-butyl(5-carboxypentyl)phosphonium bromide (**8**)

Tri-*tert*-butylphosphine (0.31 g, 1.5 mmol) was dissolved in acetonitrile (0.3 ml) and then 6-bromohexanoic acid (0.30 g, 1.5 mmol) in 1.5 ml acetonitrile was added. The mixture was stirred at 50 °C for 4 hours. The reaction mixture was cooled; the solvent was removed under reduced pressure. Solid was washed with diethyl ether. Yellowish amorphous powder, 0.48 g (78%), melting point 186 °C with decomposition.

^{1}H NMR (300 MHz, $CDCl_3$) δ 2.47 (m, 2H, P-CH_2), 1.93 (m, 8H, 4CH_2), 1.63 (d, $^{3}J_{HH}$ = 14.7 Hz, 27H, P(C(CH_3)$_3$)$_3$). ^{13}C NMR (75 MHz, $CDCl_3$) δ 175.9 (s, CO), 39.3 (d, $^{1}J_{PC}$ = 29.2 Hz, P(*C*(CH_3)$_3$)$_3$), 34.5 (s, *C*H_2-CO), 30.9 (d, $^{3}J_{PC}$ = 12.6 Hz, CH_2), 30.1 (s, P(C(*C*H_3)$_3$)$_3$), 24.4 (d, $^{2}J_{PC}$ = 6.8 Hz, P-CH_2-*C*H_2), 24.2 (s, CH_2), 18.7 (d, $^{1}J_{PC}$ = 35.4 Hz, P-CH_2). ^{31}P NMR (121.5 MHz, $CDCl_3$) δ 49.7 (s). HRMS (ESI) Calcd. for [$C_{18}H_{38}PO_2$]$^+$ [M]$^+$: 317.2604, Found: 317.2601.

Tri-*tert*-butyl(2-methoxy-2-oxoethyl)phosphonium bromide (**9**)

Tri-*tert*-butylphosphine (0.97 g, 4.8 mmol) and methyl bromoacetate (0.45 mL, 4.8 mmol) were stirred at 100 °C for 6 hours. The reaction mixture was cooled; white solid was washed with diethyl ether. White crystalline powder, 1.31 g (81%), melting point 170 °C with decomposition.

^{1}H NMR (300 MHz, $CDCl_3$) δ 4.27 (d, $^{3}J_{PH}$ = 12.5 Hz, 2H, P-CH_2), 1.72 (d, $^{3}J_{HH}$ = 14.7 Hz, 27H, P(C(CH_3)$_3$)$_3$), 0.80 (s, 3H, O-CH_3). ^{13}C NMR (75 MHz, $CDCl_3$) δ 166.2 (d, $^{2}J_{PC}$ = 4.1 Hz, CO), 53,6 (s, CH_3), 40.1 (d, $^{1}J_{PC}$ = 27.7 Hz, P(*C*(CH_3)$_3$)$_3$), 29.9 (s, P(C(*C*H_3)$_3$)$_3$), 25.5 (d, $^{1}J_{PC}$ = 38.0 Hz, P-CH_2). ^{31}P NMR (121.5 MHz, $CDCl_3$) δ 51.9 (s). HRMS (ESI) Calcd. for [$C_{15}H_{32}PO_2$]$^+$ [M]$^+$: 275.2134, Found: 275.2136.

Tri-*tert*-butyl(2-ethoxy-2-oxoethyl)phosphonium bromide (**10**)

Tri-*tert*-butylphosphine (1.07 g, 5.2 mmol) and ethyl bromoacetate (0.59 mL, 5.2 mmol) were stirred at 100 °C for 4 hours. The reaction mixture was cooled; white solid was washed with diethyl ether. White crystalline powder, 1.67 g (86%), melting point 168 °C with decomposition.

^{1}H NMR (300 MHz, $CDCl_3$) δ 4.21 (q, $^{3}J_{HH}$ = 7.1 Hz, 2H, C*H*$_2$-CH_3), 4.13 (d, $^{2}J_{PH}$ = 12.7 Hz, 2H, P-CH_2), 1.69 (d, $^{3}J_{PH}$ = 14.7 Hz, 27H, P(C(CH_3)$_3$)$_3$), 1.29 (t, $^{3}J_{HH}$ = 7.1 Hz, 3H, CH_2-CH_3). ^{13}C NMR (75 MHz, $CDCl_3$) δ 165.6 (d, $^{2}J_{PC}$ = 4.1 Hz, CO), 63.0 (s, *C*H_2-CH_3), 40.1 (d, $^{1}J_{PC}$ = 27.6 Hz, P(*C*(CH_3)$_3$)$_3$), 29.9 (s, P(C(*C*H_3)$_3$)$_3$), 25.5 (d, $^{1}J_{PC}$ = 37.4 Hz, P-CH_2), 13.9 (s, CH_2-*C*H_3). ^{31}P NMR (121.5 MHz, $CDCl_3$) δ 51.9 (s). HRMS (ESI) Calcd. for [$C_{16}H_{34}PO_2$]$^+$ [M]$^+$: 289.2291, Found: 289.2292.

Tri-*tert*-butyl(2-*iso*-propoxy-2-oxoethyl)phosphonium bromide (**11**)

Tri-*tert*-butylphosphine (0.94 g, 4.6 mmol) and isopropyl bromoacetate (0.60 mL, 4.6 mmol) were stirred at 100 °C for 6 hours. The reaction mixture was cooled; white solid was washed with diethyl ether. White crystalline powder, 1.40 g (83%), melting point 170 °C with decomposition.

^{1}H NMR (300 MHz, $CDCl_3$) δ 5.05 (pent, $^{3}J_{HH}$ = 6.4 Hz, 1H, O-CH), 4.10 (d, $^{2}J_{PH}$ =13.0 Hz, 2H, P-CH_2), 1.72 (d, $^{3}J_{PH}$ = 14.5 Hz, 27H, P(C(C*H*$_3$)$_3$)$_3$), 1.30 (d, $^{3}J_{HH}$ = 7.3 Hz, 6H, CH-(C*H*$_3$)$_2$). ^{13}C NMR (75 MHz, $CDCl_3$) δ 165.0 (d, $^{2}J_{PC}$ = 4.2 Hz, CO), 71.3 (s, *C*H), 40.1 (d, $^{1}J_{PC}$ = 27.9 Hz, P(*C*(CH_3)$_3$)$_3$), 29.9 (s, P(C(*C*H_3)$_3$)$_3$), 25.8 (d, $^{1}J_{PC}$ = 37.4 Hz, P-CH_2), 21.5 (s, CH-(*C*H_3)$_2$). ^{31}P NMR (121.5 MHz, $CDCl_3$) δ 52.0 (s). HRMS (ESI) Calcd. for [$C_{17}H_{36}PO_2$]$^+$ [M]$^+$: 303.2447, Found: 303.2447.

Tri-*tert*-butyl(2-butoxy-2-oxoethyl)phosphonium bromide (**12**)

Tri-*tert*-butylphosphine (0.99 g, 4.8 mmol) and butyl bromoacetate (0.70 mL, 4.8 mmol) were stirred at 100 °C for 6 hours. The reaction mixture was cooled; white solid was washed with diethyl ether. White crystalline powder, 1.51 g (87%), melting point 125 °C.

^{1}H NMR (300 MHz, $CDCl_3$) δ 4.11 (t, $^{3}J_{HH}$ = 6.94 Hz, 2H, O-CH_2), 4.07 (d, $^{2}J_{PH}$ = 12.83 Hz, 2H, P-CH_2), 1.67 (d, $^{3}J_{PH}$ = 14.7 Hz, 27H, P($C(CH_3)_3)_3$), 1.60 (m, 2H, CH_2), 1.34 (m, 2H, CH_2), 0.88 (t, $^{3}J_{HH}$ = 7.14 Hz, 3H, CH_2-CH_3). ^{13}C NMR (75 MHz, $CDCl_3$) δ 165.6 (d, $^{2}J_{PC}$ = 3.9 Hz, CO), 66.9 (s, O-CH_2), 40.1 (d, $^{1}J_{PC}$ = 27.5 Hz, P(*C*$(CH_3)_3)_3$), 29.9 (s, P(C(*C*$H_3)_3)_3$), 27.8 (s, CH_2), 25.4 (d, $^{1}J_{PC}$ = 37.7 Hz, P-CH_2), 19.0 (s, CH_2), 13.6 (s, CH_2-*C*H_3). ^{31}P NMR (121.5 MHz, $CDCl_3$) δ 52.0 (s). HRMS (ESI) Calcd. for $[C_{18}H_{38}PO_2]^+$ $[M]^+$: 317.2604, Found: 317.2604.

NMR spectra

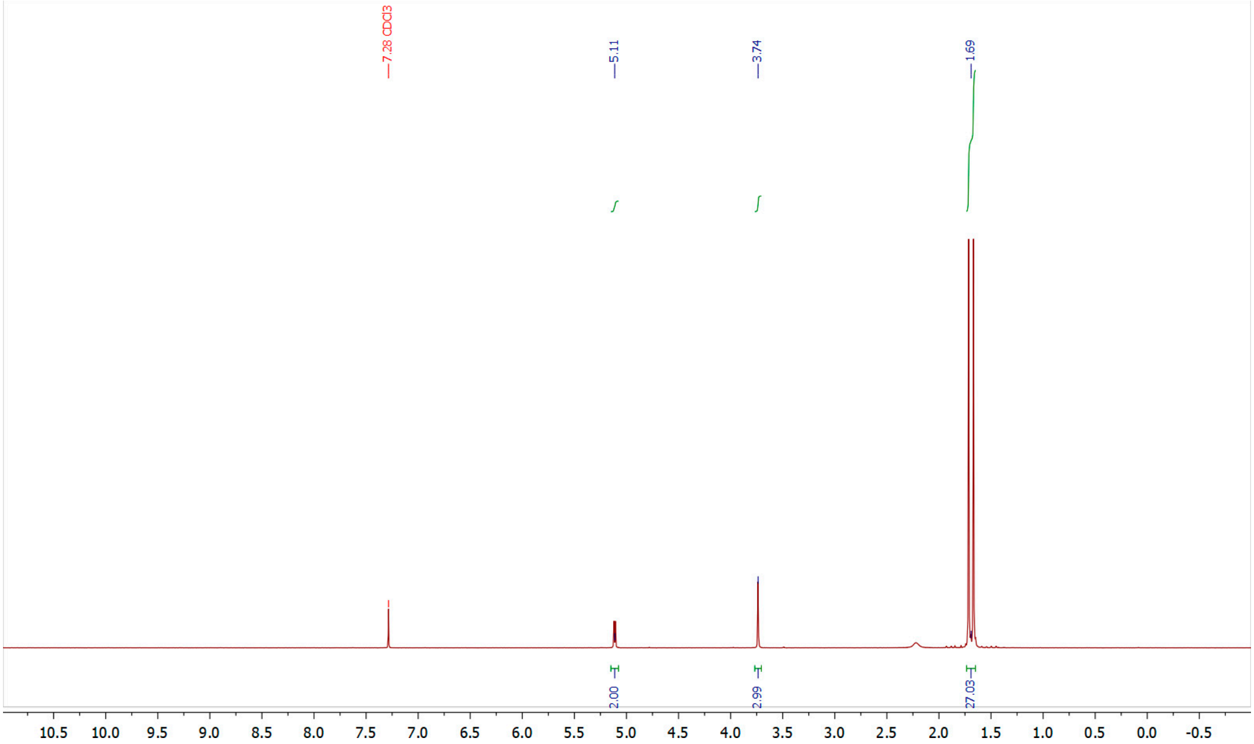

Figure S1. The ^{1}H NMR (300 MHz, $CDCl_3$) of tri-*tert*-butyl(methoxymethyl)phosphonium chloride (**1**).

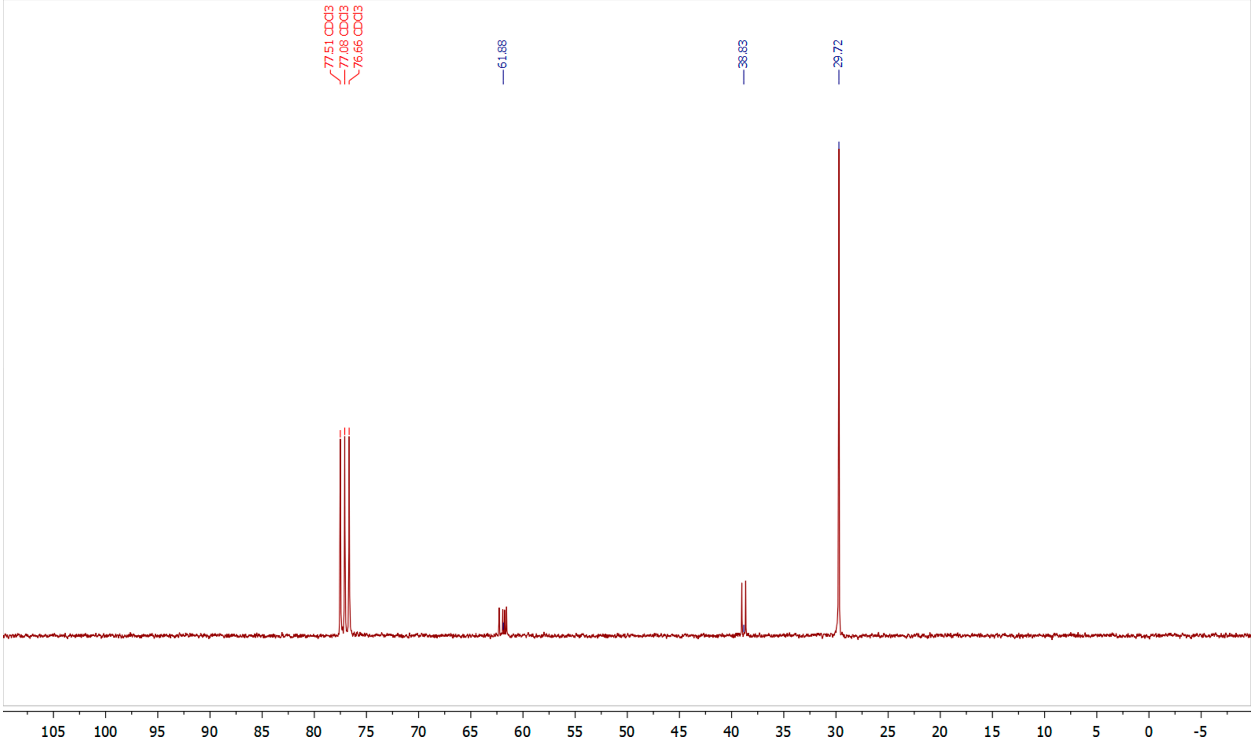

Figure S2. The $^{13}C\{^{1}H\}$ NMR (75 MHz, $CDCl_3$) of tri-*tert*-butyl(methoxymethyl)phosphonium chloride (**1**).

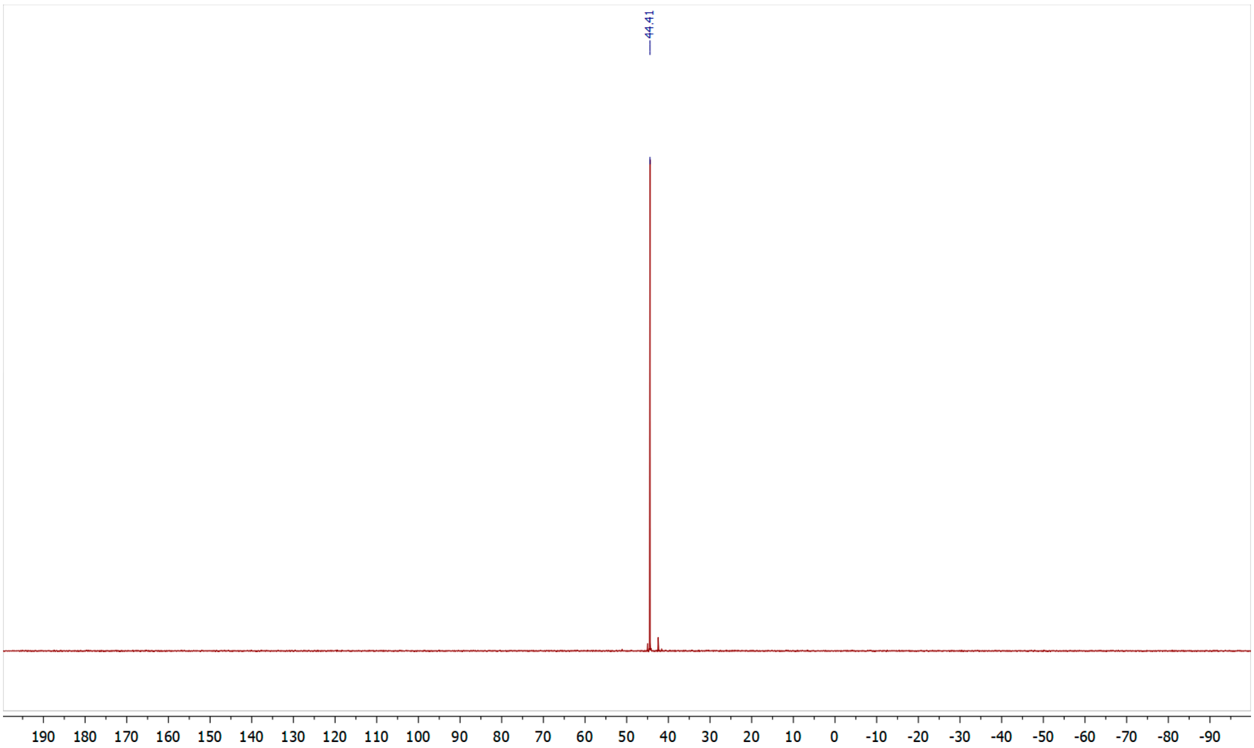

Figure S3. The $^{31}P\{^{1}H\}$ NMR (121.5 MHz, $CDCl_3$) of tri-*tert*-butyl(methoxymethyl)phosphonium chloride (**1**).

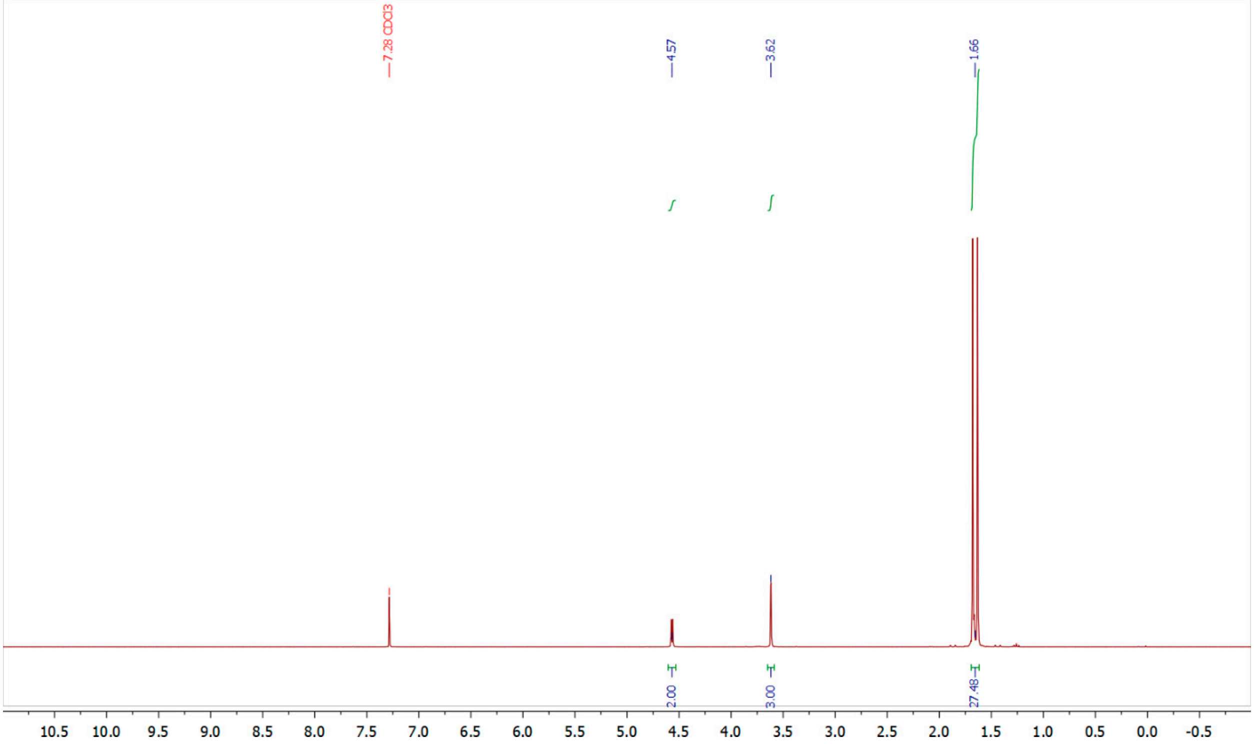

Figure S4. The ^{1}H NMR (300 MHz, $CDCl_3$) of tri-*tert*-butyl(methoxymethyl)phosphonium tetrafluoroborate (**2**).

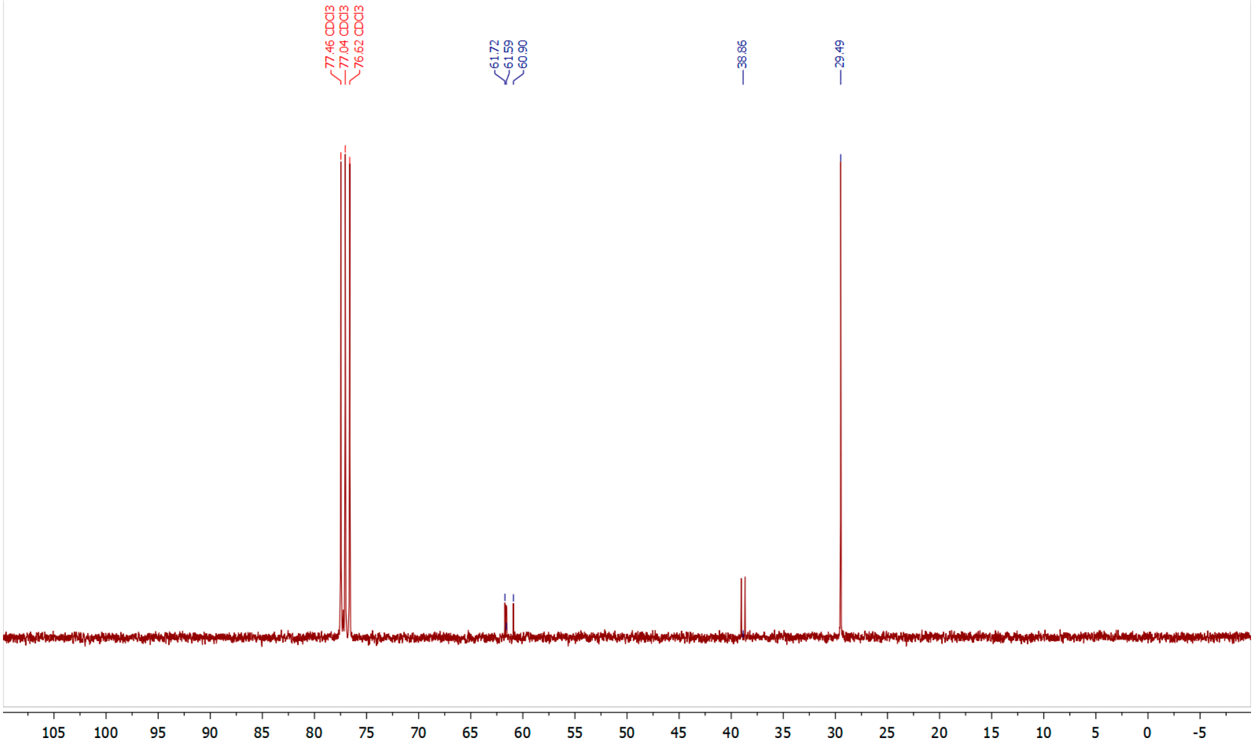

Figure S5. The $^{13}C\{^{1}H\}$ NMR (75 MHz, $CDCl_3$) of tri-*tert*-butyl(methoxymethyl)phosphonium tetrafluoroborate (**2**).

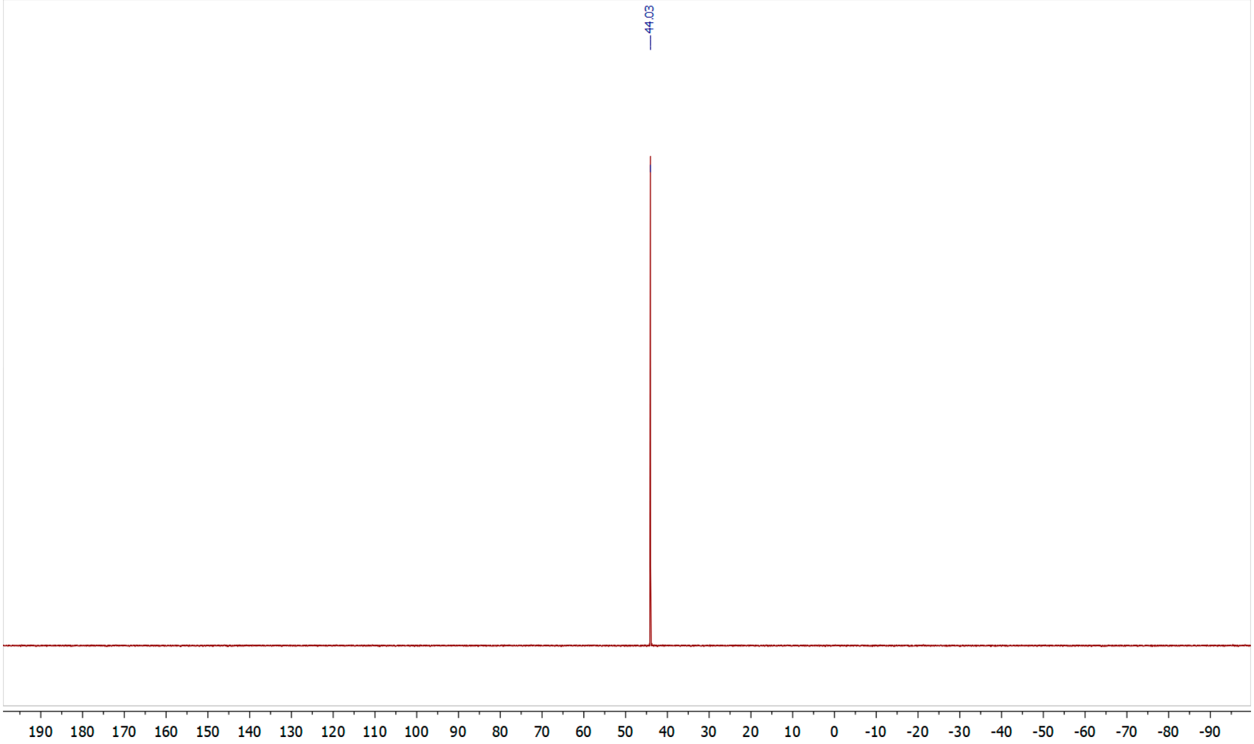

Figure S6. The $^{31}P\{^{1}H\}$ NMR (121.5 MHz, $CDCl_3$) of tri-*tert*-butyl(methoxymethyl)phosphonium tetrafluoroborate (**2**).

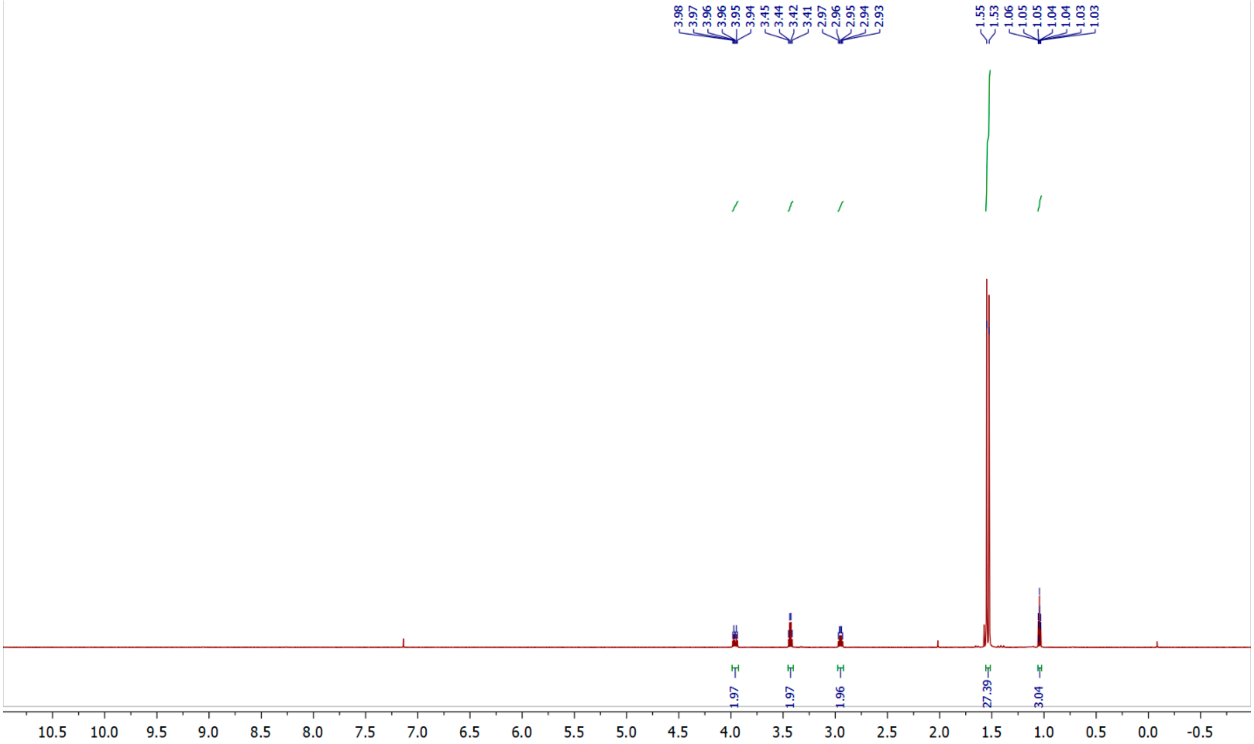

Figure S7. The ^{1}H NMR (300 MHz, $CDCl_3$) of tri-*tert*-butyl(2-ethoxyethyl)phosphonium bromide (**3**).

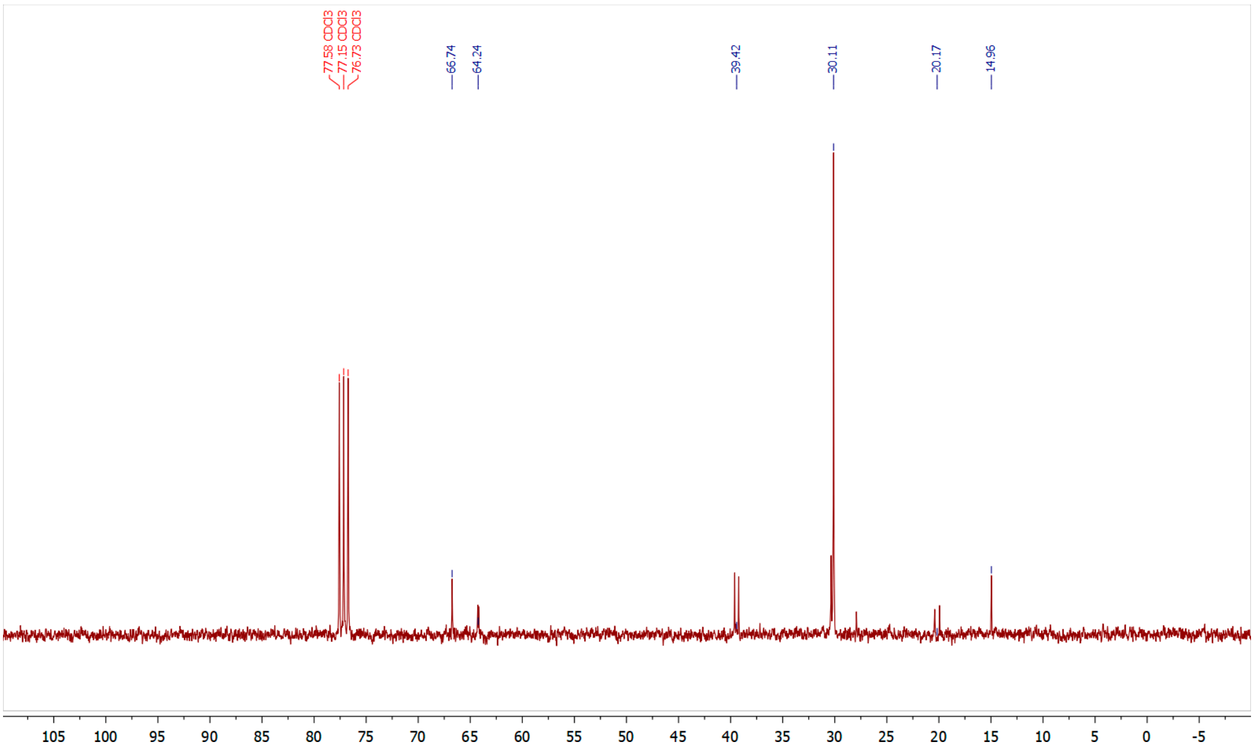

Figure S8. The $^{13}C\{^1H\}$ NMR (75 MHz, $CDCl_3$) of tri-*tert*-butyl(2-ethoxyethyl)phosphonium bromide (**3**).

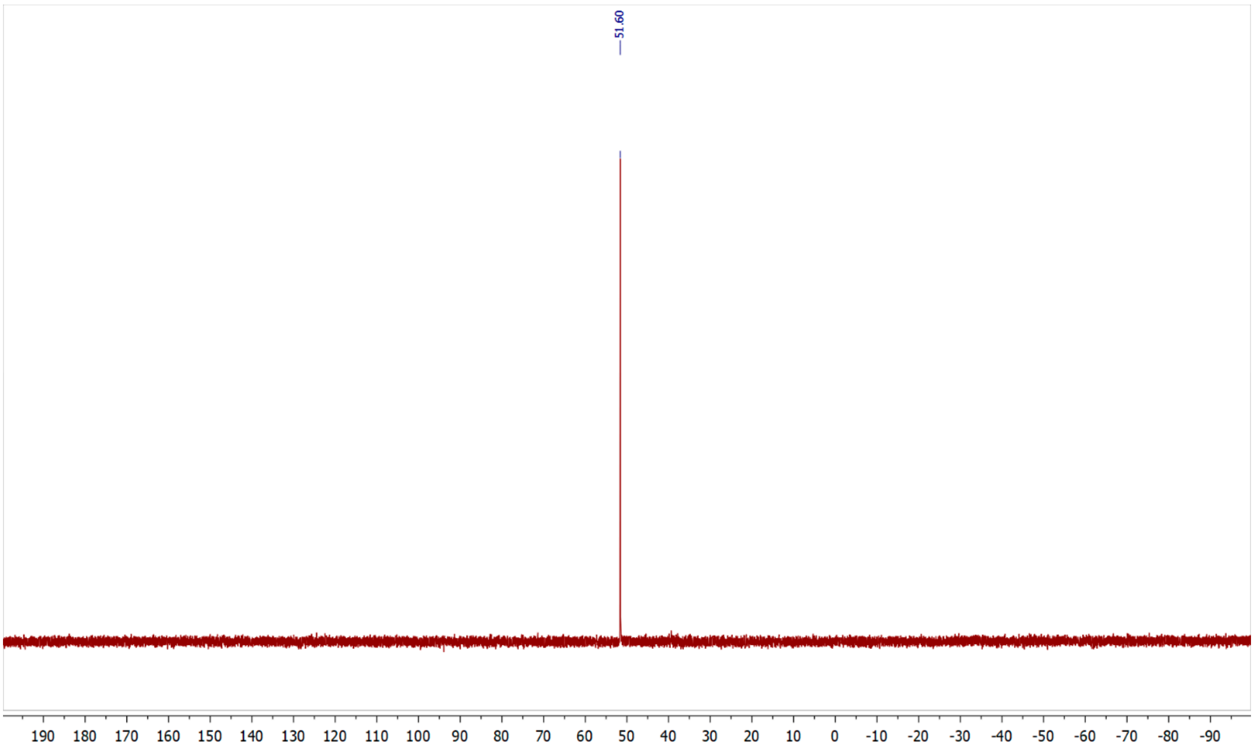

Figure S9. The $^{31}P\{^1H\}$ NMR (121.5 MHz, $CDCl_3$) of tri-*tert*-butyl(2-ethoxyethyl)phosphonium bromide (**3**).

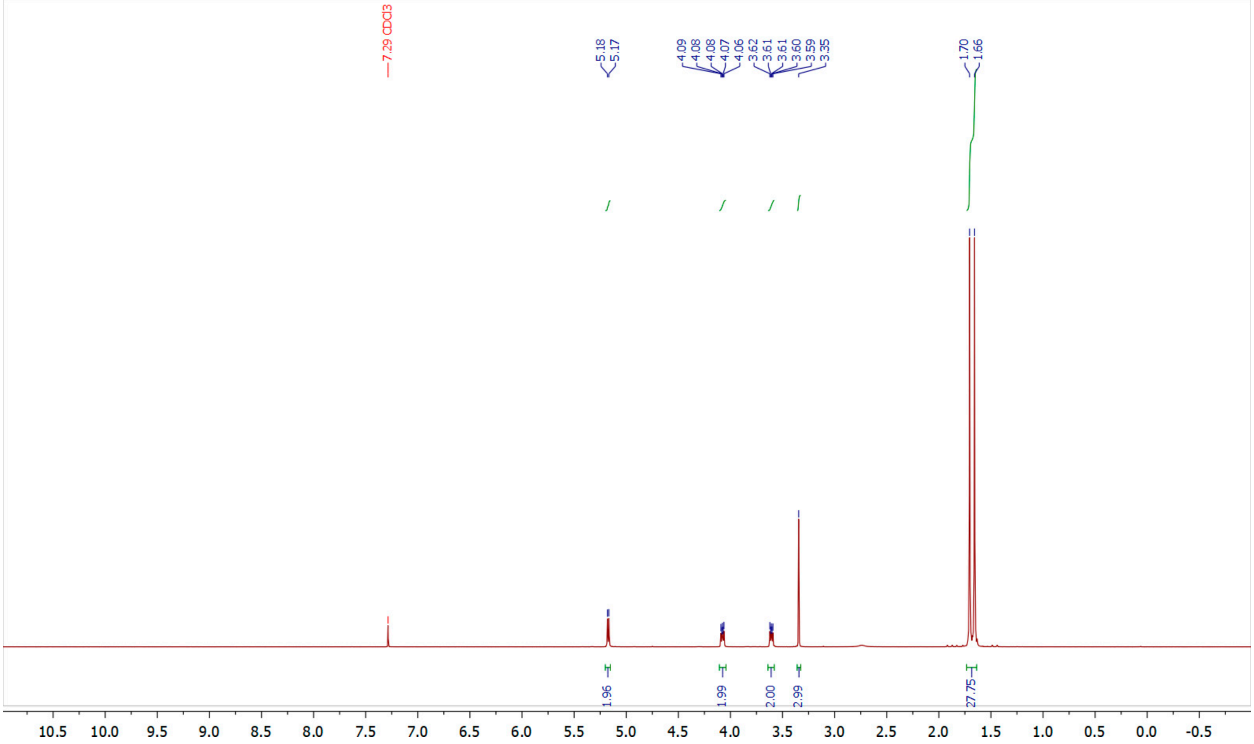

Figure S10. The ^{1}H NMR (300 MHz, $CDCl_3$) of tri-*tert*-butyl((2-methoxyethoxy)methyl)phosphonium chloride (**4**).

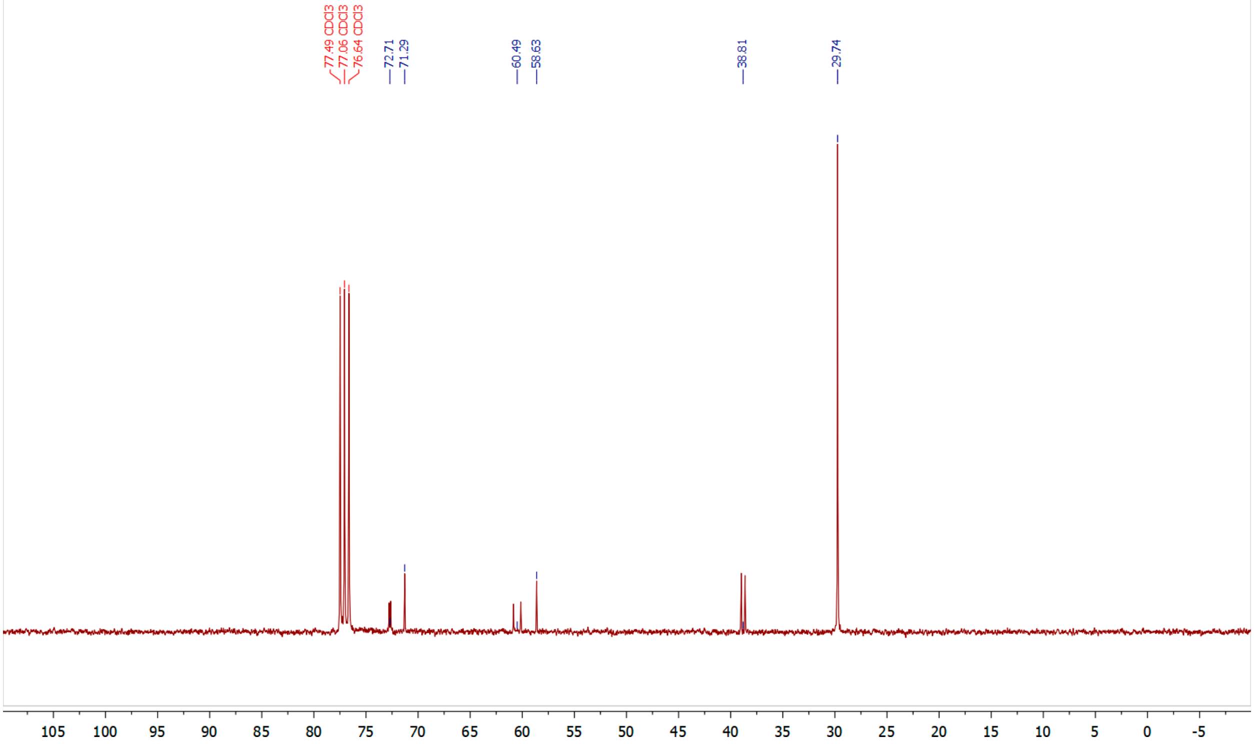

Figure S11. The $^{13}C\{^{1}H\}$ NMR (75 MHz, $CDCl_3$) of tri-*tert*-butyl((2-methoxyethoxy)methyl)phosphonium chloride (**4**).

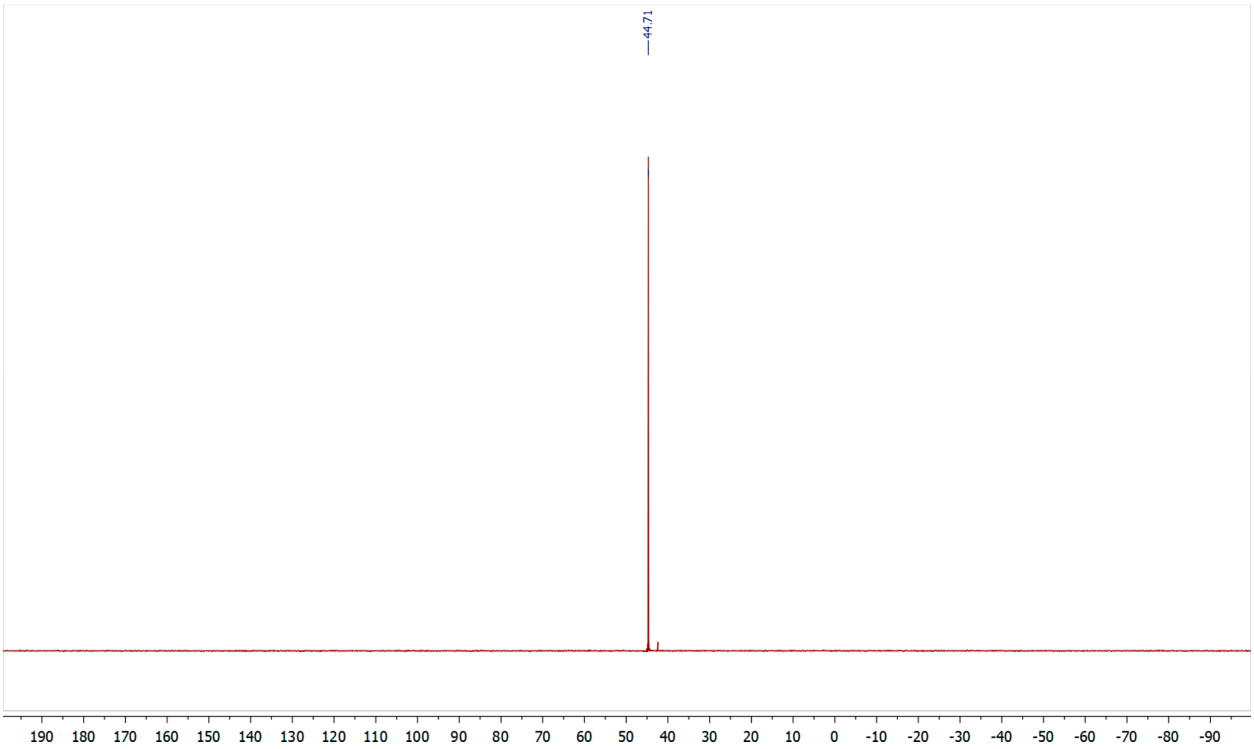

Figure S12. The $^{31}P\{^{1}H\}$ NMR (121.5 MHz, $CDCl_3$) of tri-*tert*-butyl((2-methoxyethoxy)methyl)phosphonium chloride (**4**).

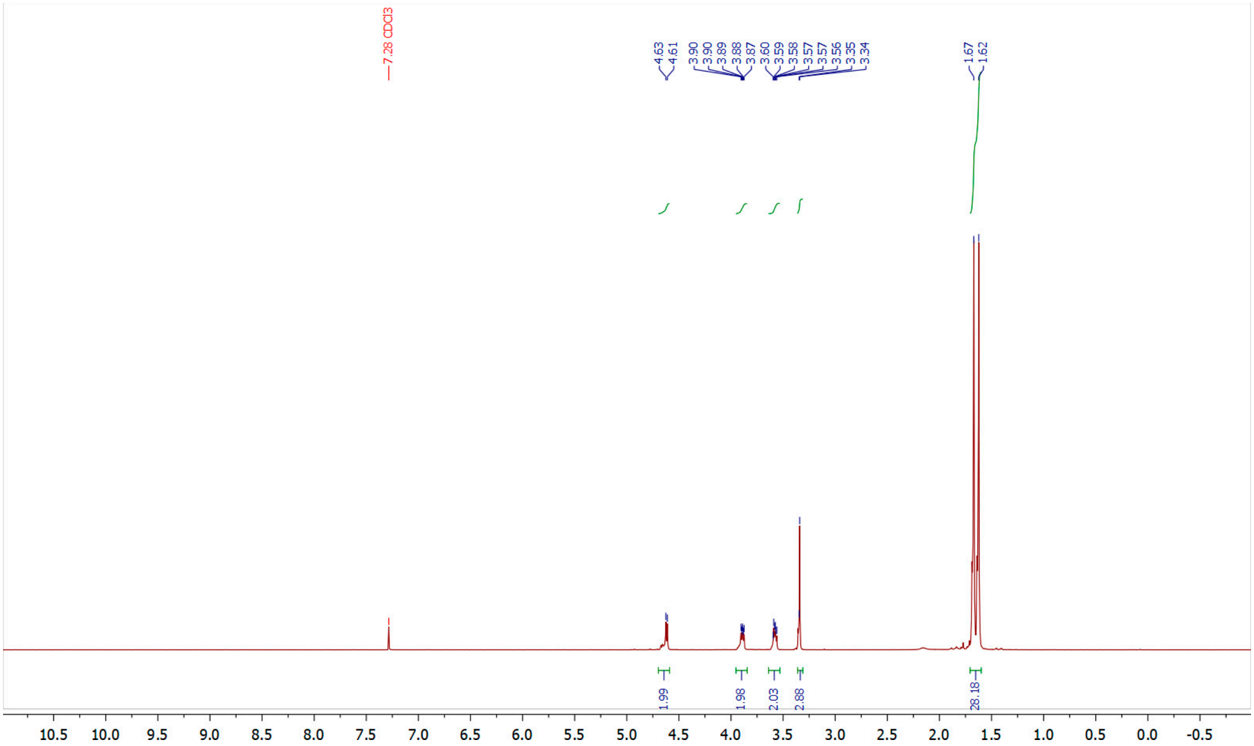

Figure S13. The ^{1}H NMR (300 MHz, $CDCl_3$) of tri-*tert*-butyl((2-methoxyethoxy)methyl)phosphonium tetrafluoroborate (**5**).

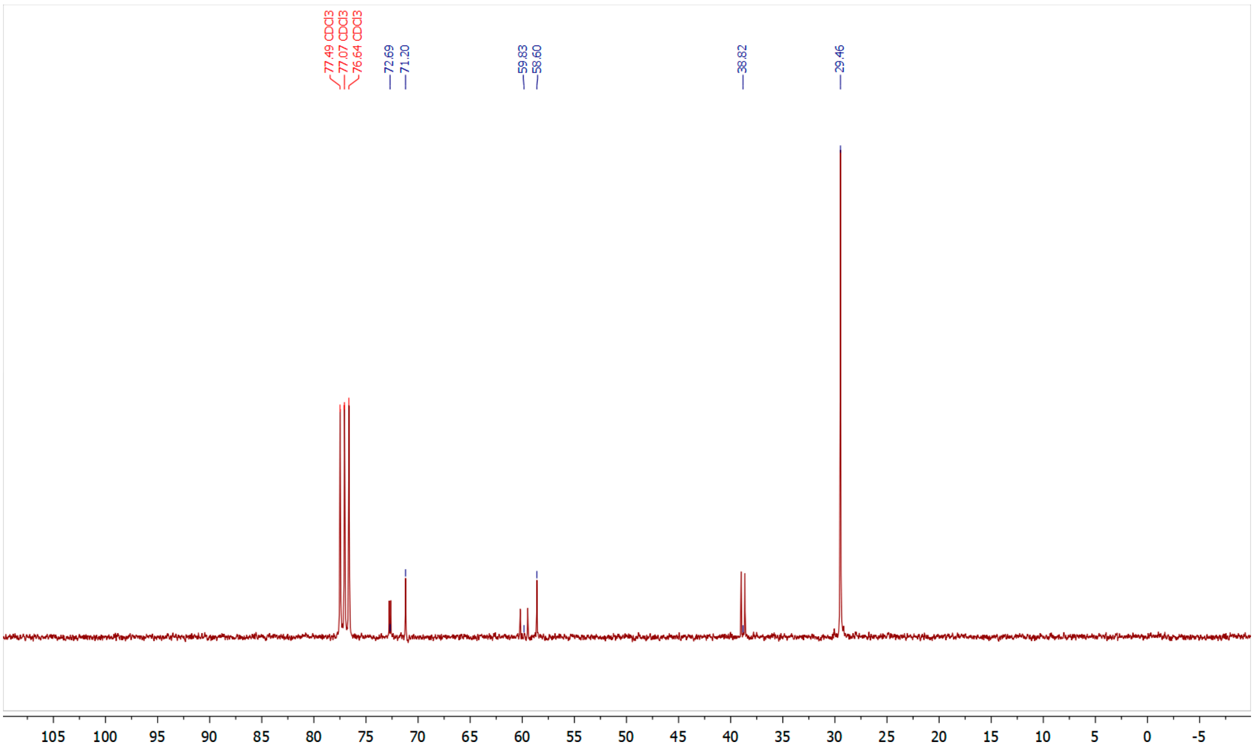

Figure S14. The $^{13}C\{^1H\}$ NMR (75 MHz, $CDCl_3$) of tri-*tert*-butyl((2-methoxyethoxy)methyl)phosphonium tetrafluoroborate (**5**).

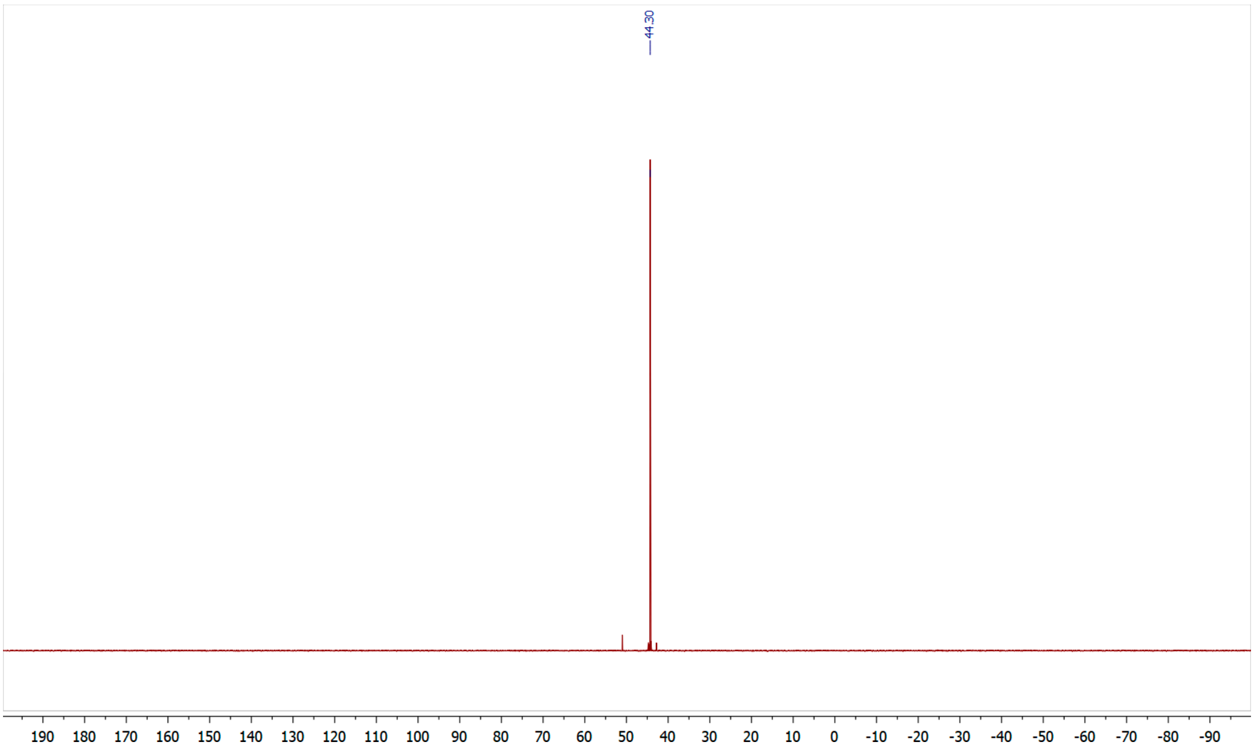

Figure S15. The $^{31}P\{^1H\}$ NMR (121.5 MHz, $CDCl_3$) of tri-*tert*-butyl((2-methoxyethoxy)methyl)phosphonium tetrafluoroborate (**5**).

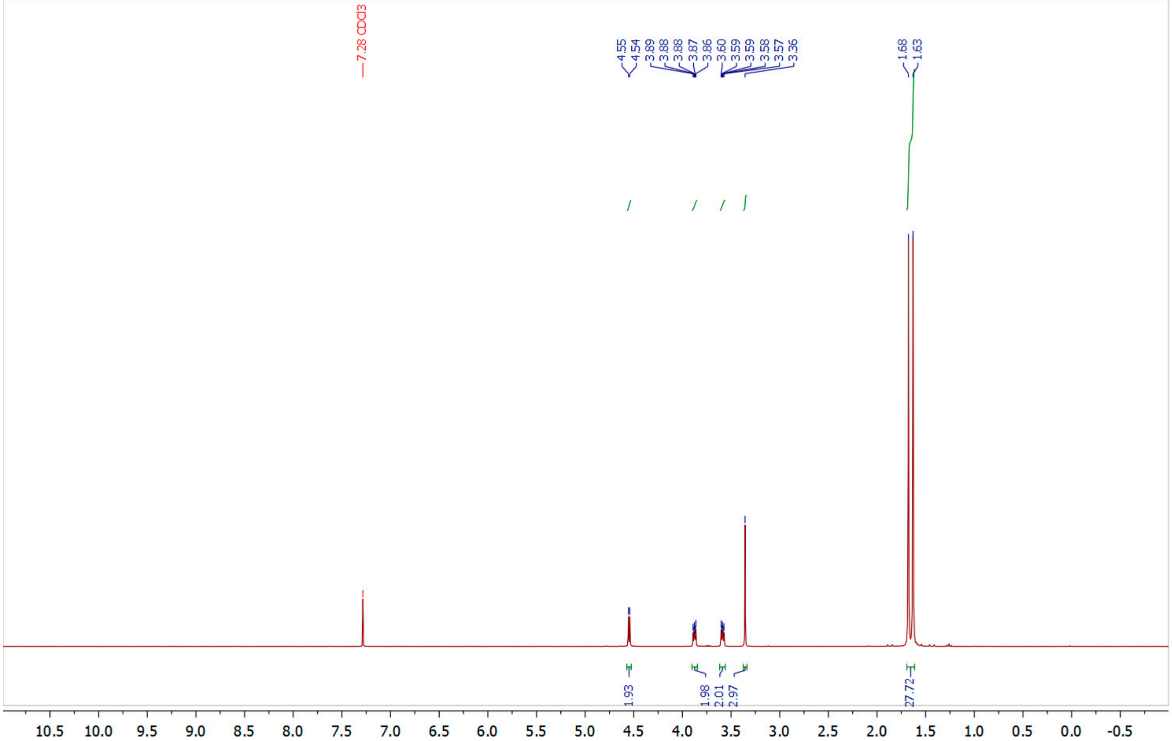

Figure S16. The ^{1}H NMR (300 MHz, $CDCl_3$) tri-*tert*-butyl((2-methoxyethoxy)methyl)phosphonium hexafluorophosphate (**6**).

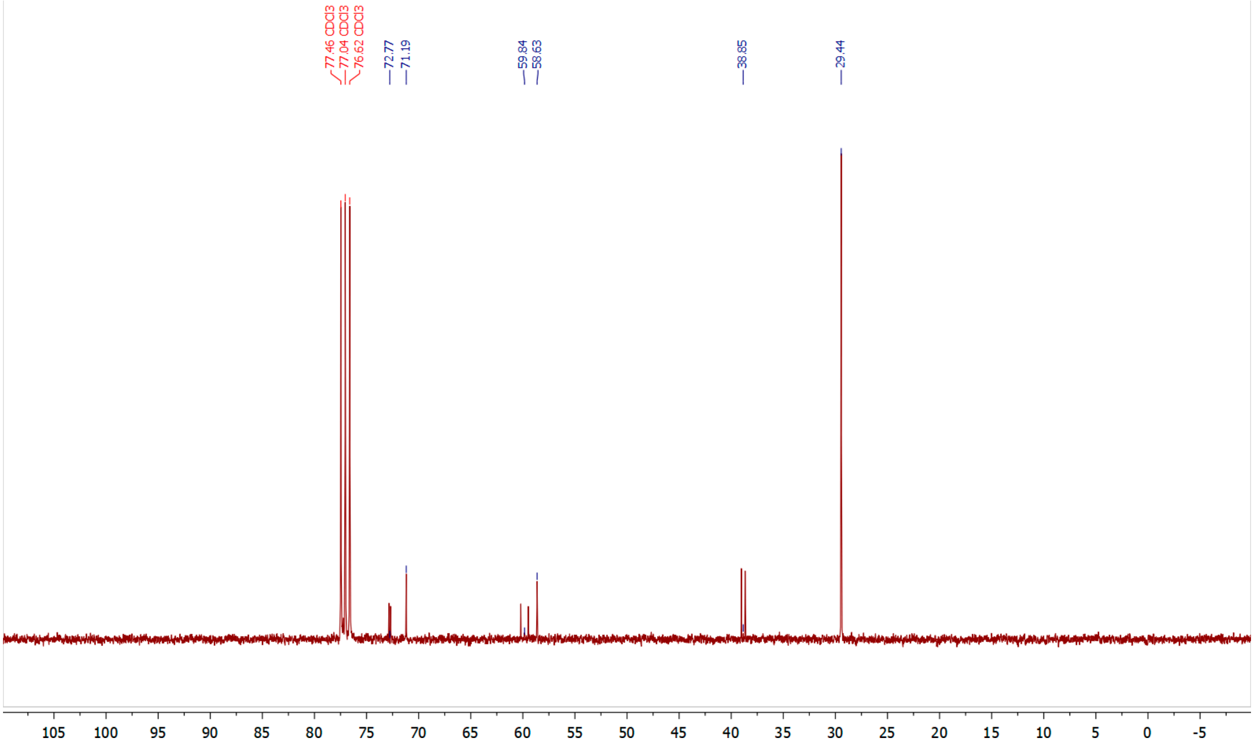

Figure S17. The $^{13}C\{^{1}H\}$ NMR (75 MHz, $CDCl_3$) of tri-*tert*-butyl((2-methoxyethoxy)methyl)phosphonium hexafluorophosphate (**6**).

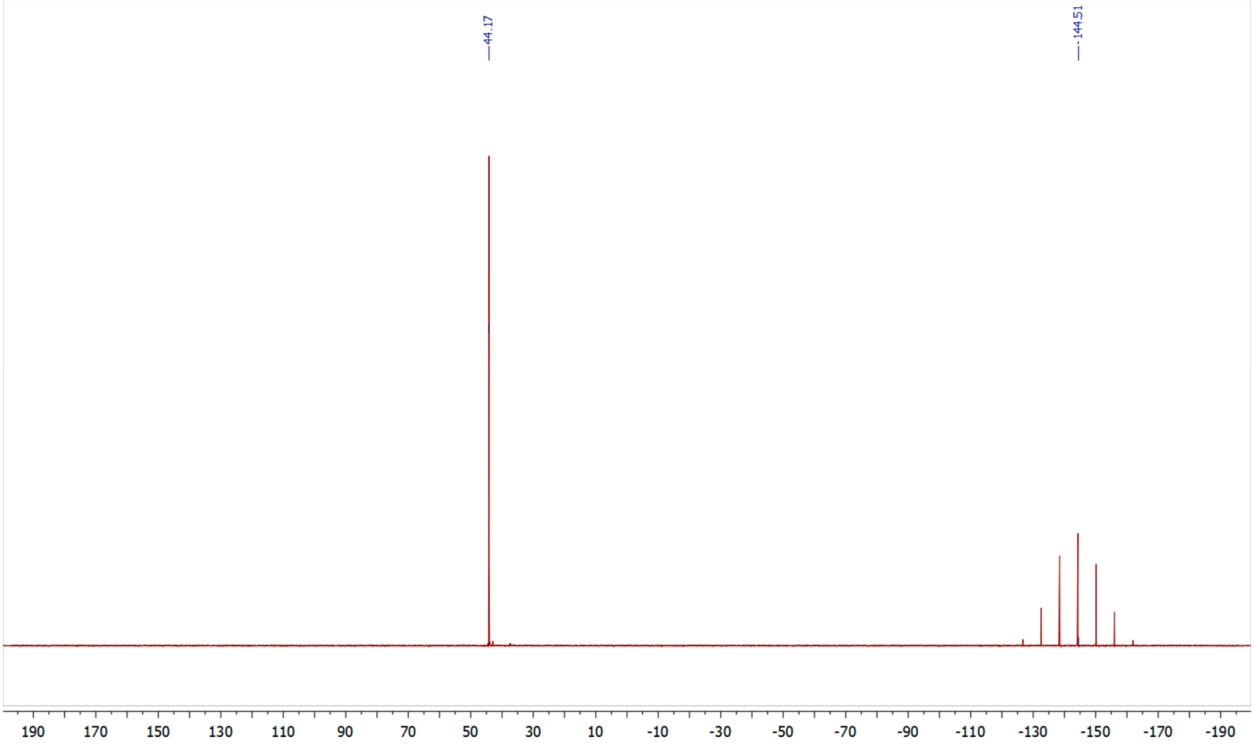

Figure S18. The $^{31}P\{^{1}H\}$ NMR (121.5 MHz, $CDCl_3$) of tri-*tert*-butyl((2-methoxyethoxy)methyl)phosphonium hexafluorophosphate (**6**).

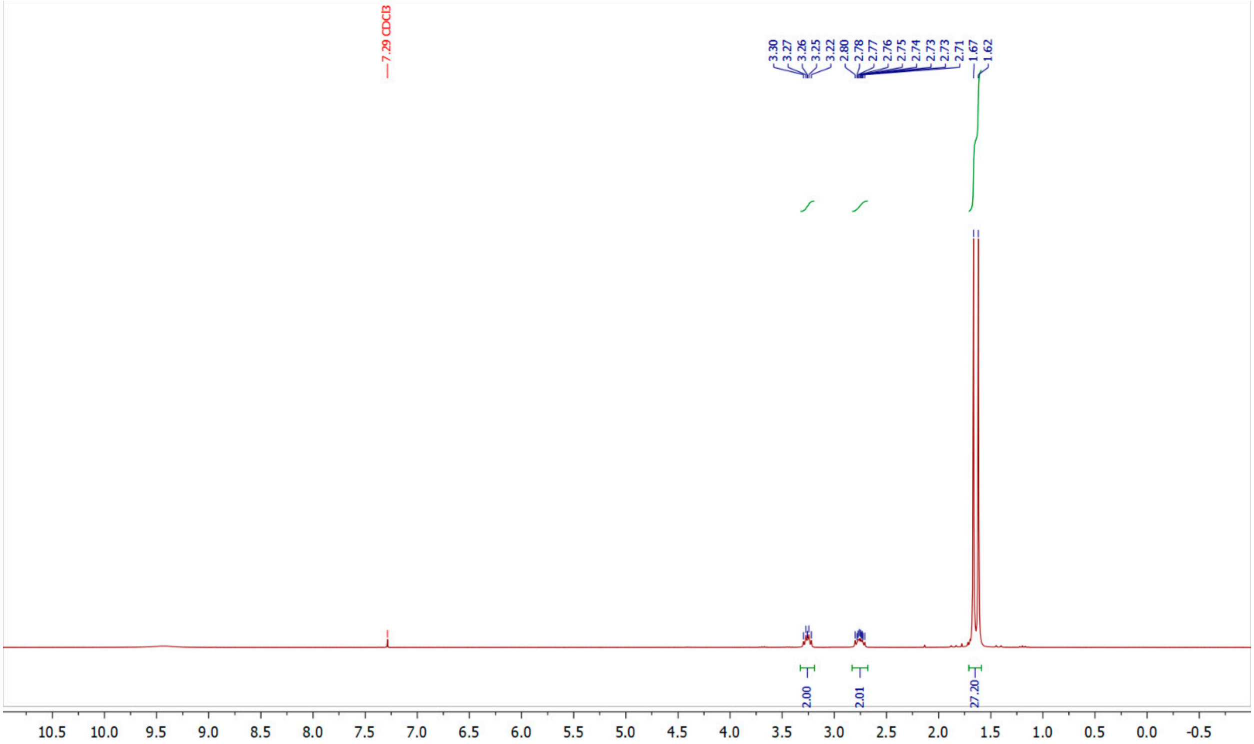

Figure S19. The ^{1}H NMR (300 MHz, $CDCl_3$) tri-*tert*-butyl(2-carboxyethyl)phosphonium bromide (**7**).

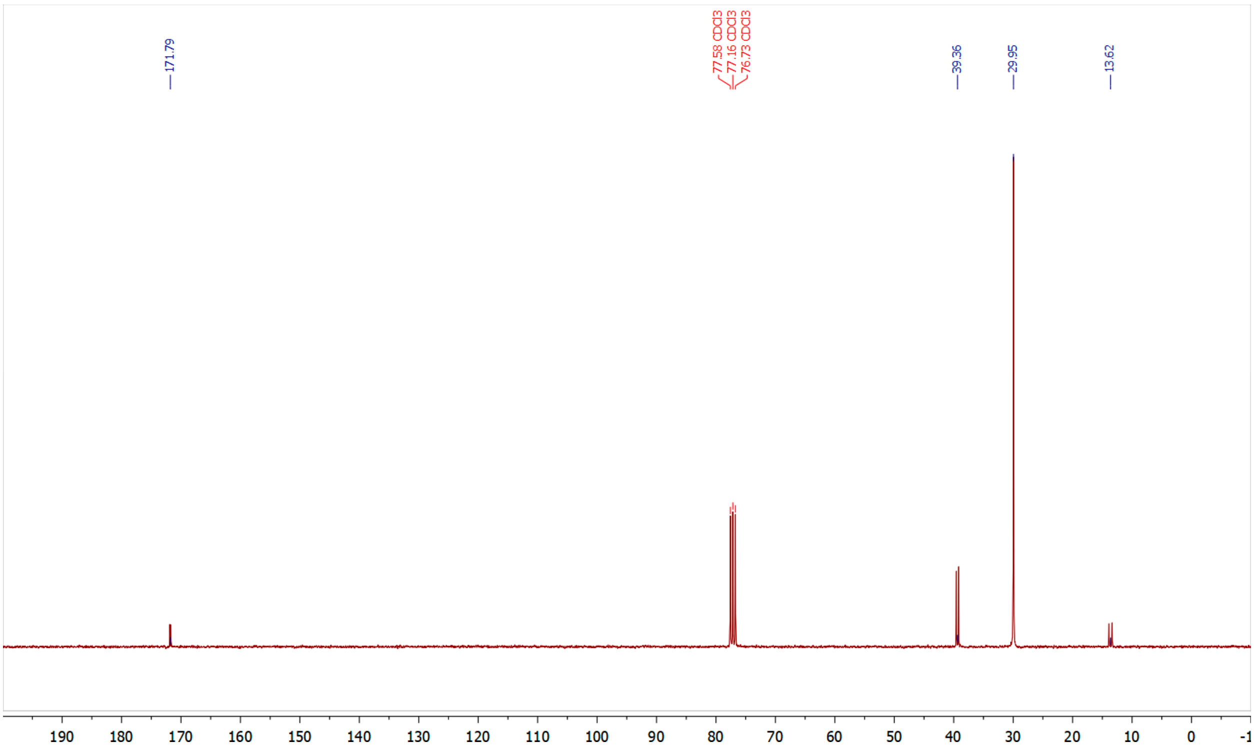

Figure S20. The $^{13}C\{^1H\}$ NMR (75 MHz, $CDCl_3$) of tri-*tert*-butyl(2-carboxyethyl)phosphonium bromide (**7**).

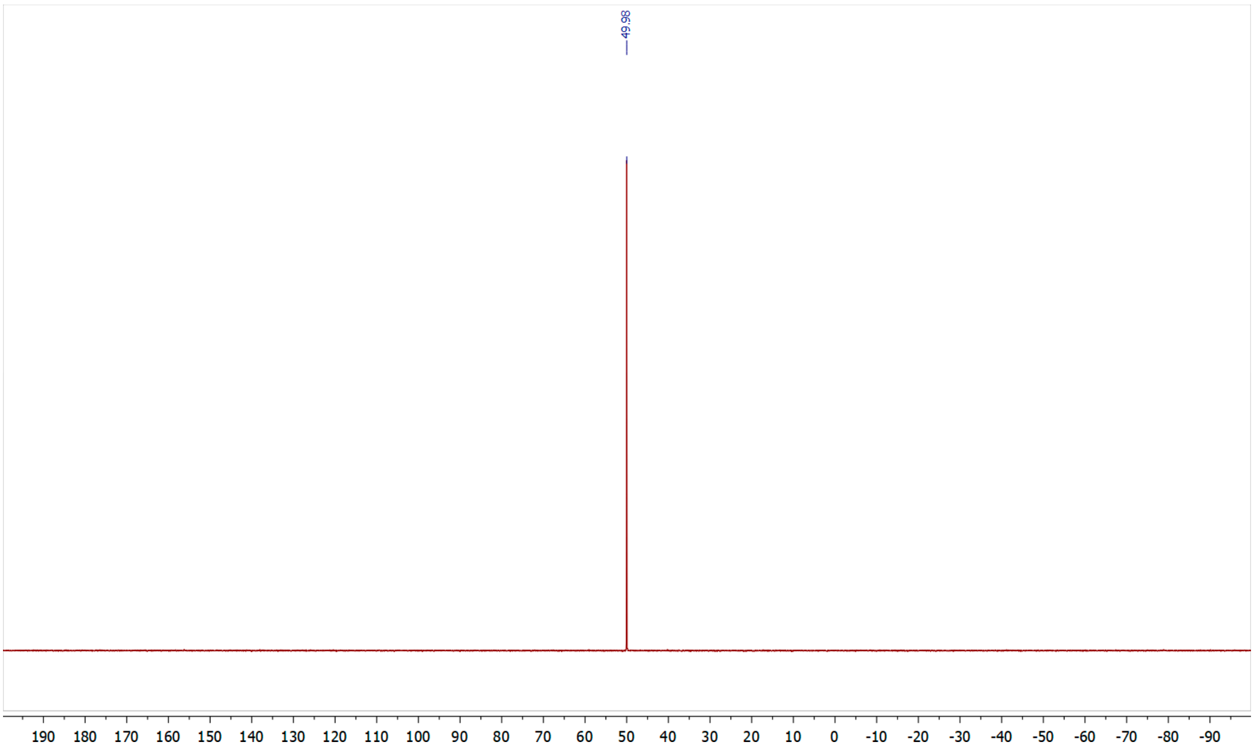

Figure S21. The $^{31}P\{^1H\}$ NMR (121.5 MHz, $CDCl_3$) of tri-*tert*-butyl(2-carboxyethyl)phosphonium bromide (**7**).

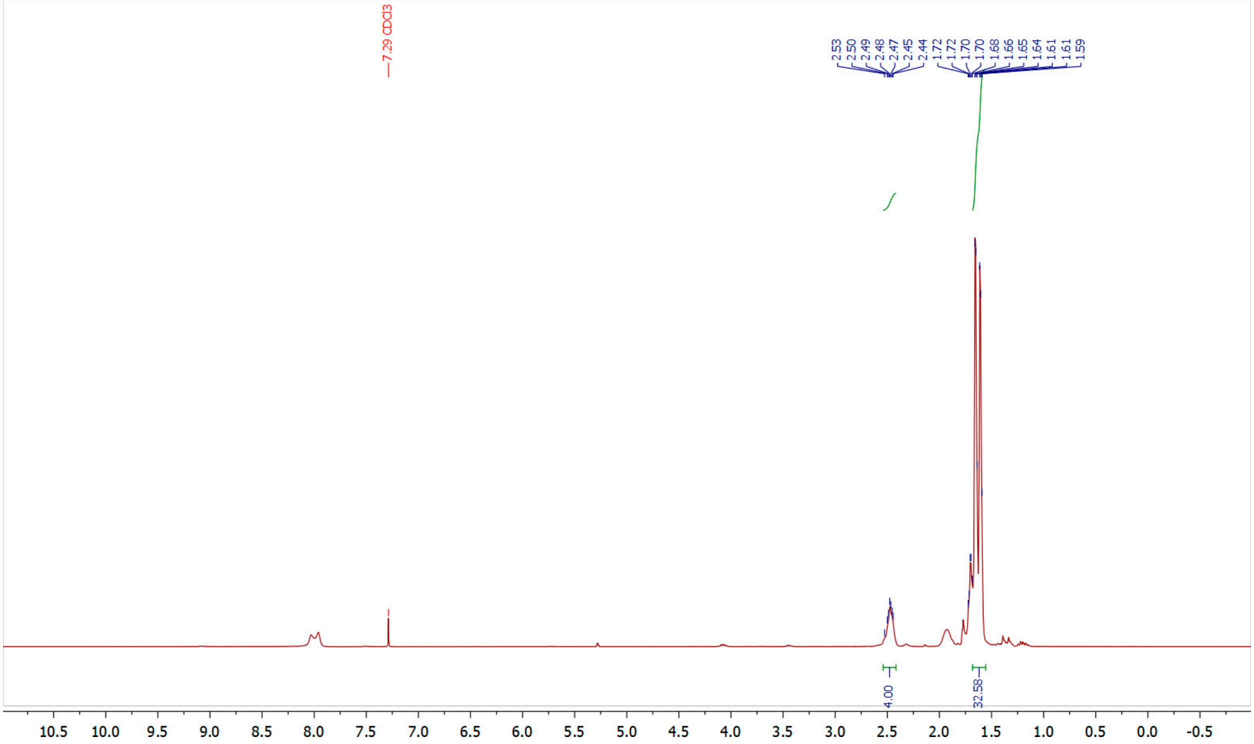

Figure S22. The ^{1}H NMR (300 MHz, $CDCl_3$) tri-*tert*-butyl(5-carboxypentyl)phosphonium bromide (**8**).

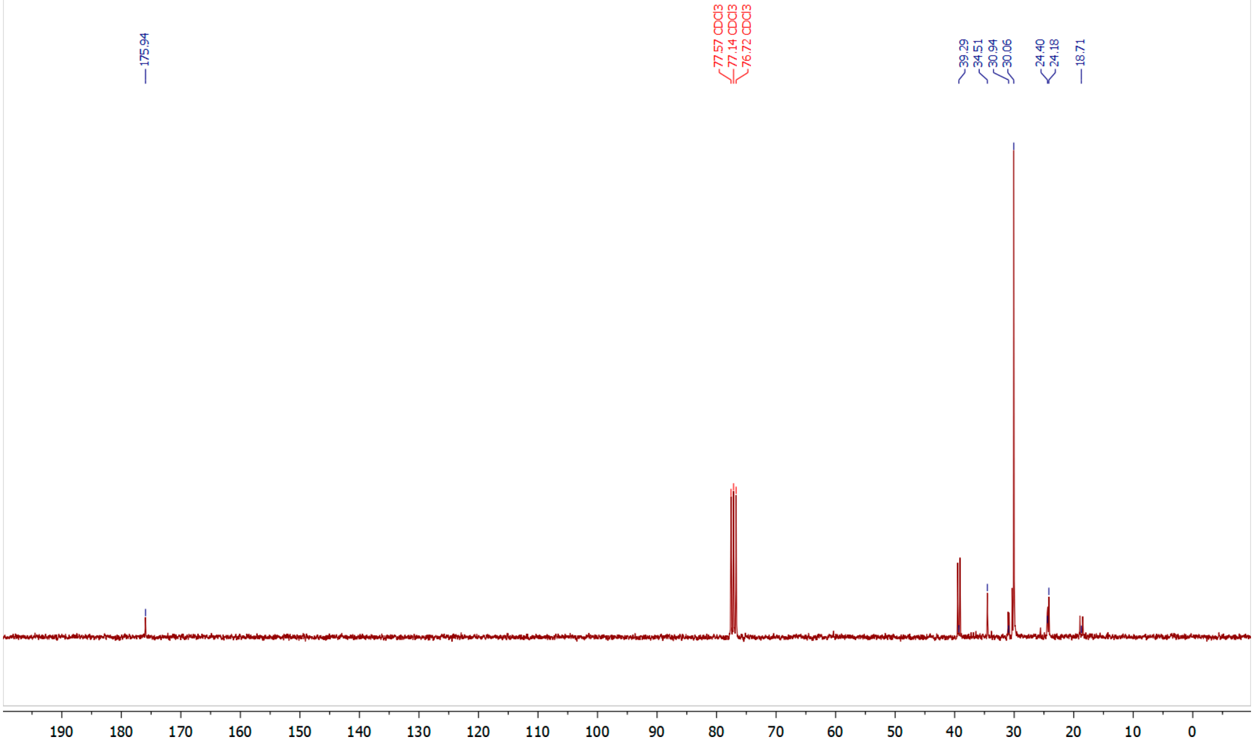

Figure S23. The $^{13}C\{^{1}H\}$ NMR (75 MHz, $CDCl_3$) of tri-*tert*-butyl(5-carboxypentyl)phosphonium bromide (**8**).

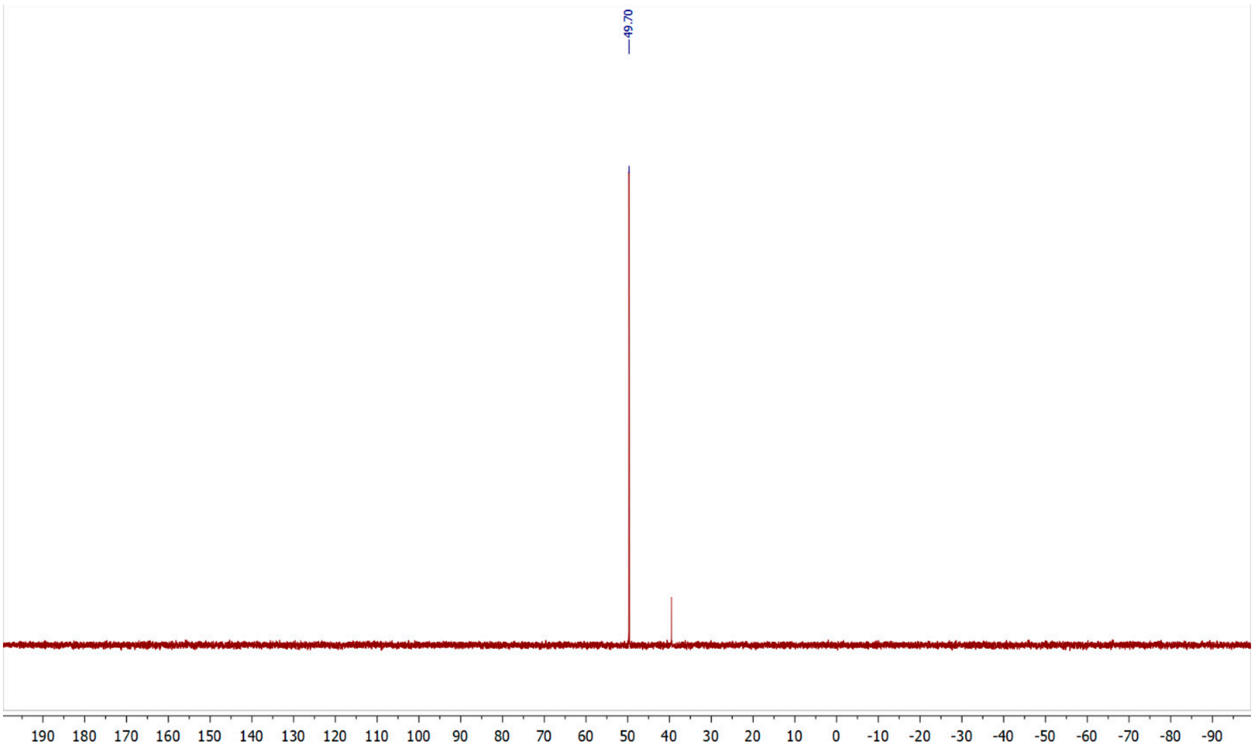

Figure S24. The $^{31}P\{^{1}H\}$ NMR (121.5 MHz, $CDCl_3$) of tri-*tert*-butyl(5-carboxypentyl)phosphonium bromide (**8**).

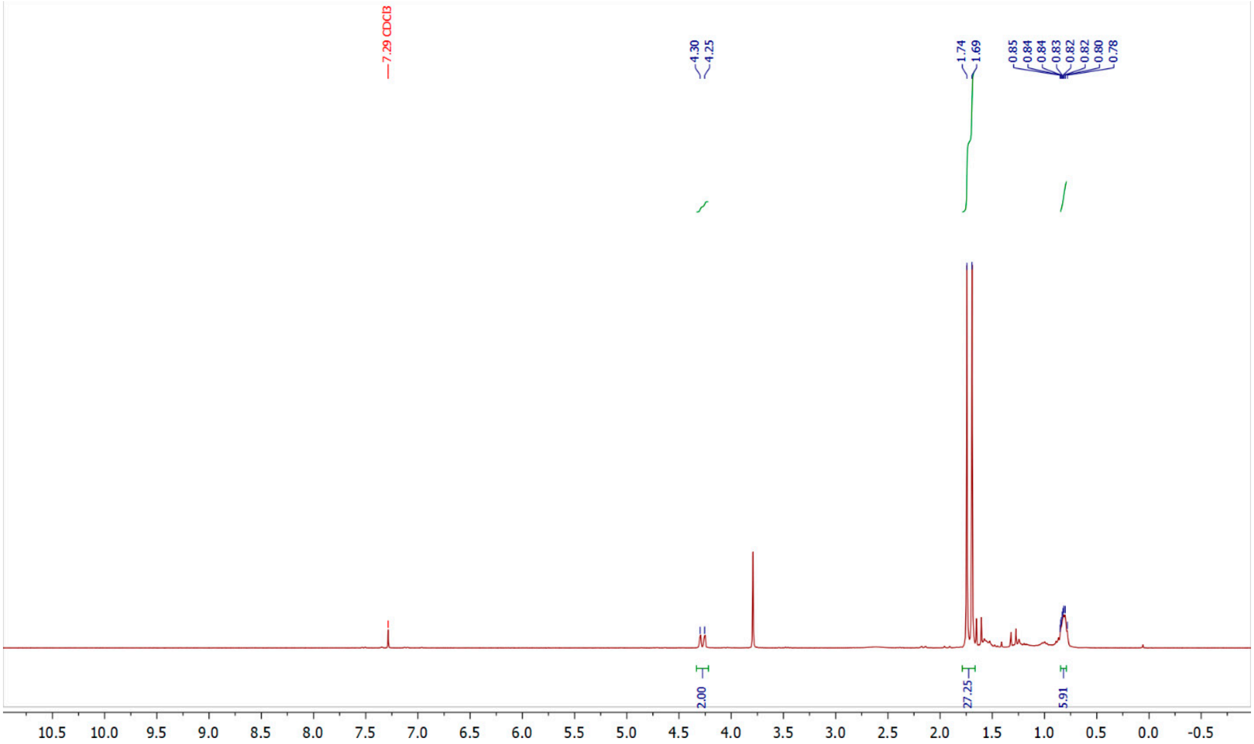

Figure S25. The ^{1}H NMR (300 MHz, $CDCl_3$) tri-*tert*-butyl(2-methoxy-2-oxoethyl)phosphonium bromide (**9**).

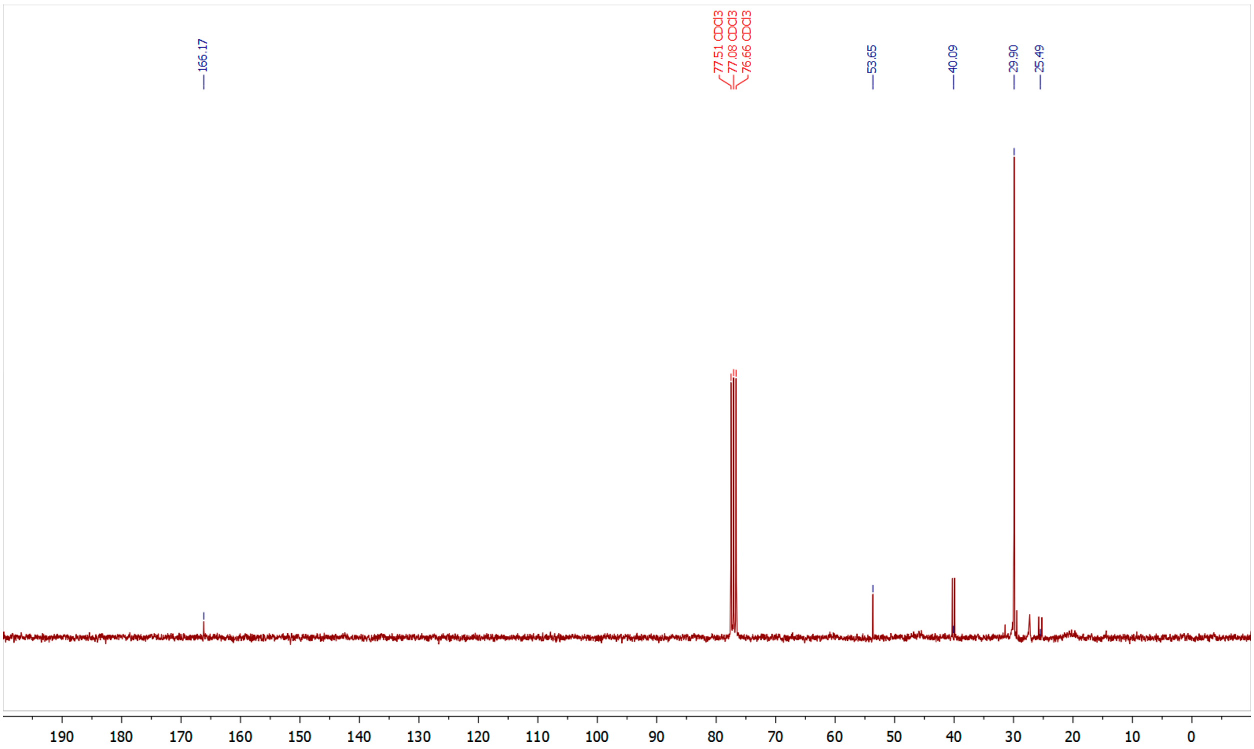

Figure S26. The $^{13}C\{^{1}H\}$ NMR (75 MHz, $CDCl_3$) of tri-*tert*-butyl(2-methoxy-2-oxoethyl)phosphonium bromide (**9**).

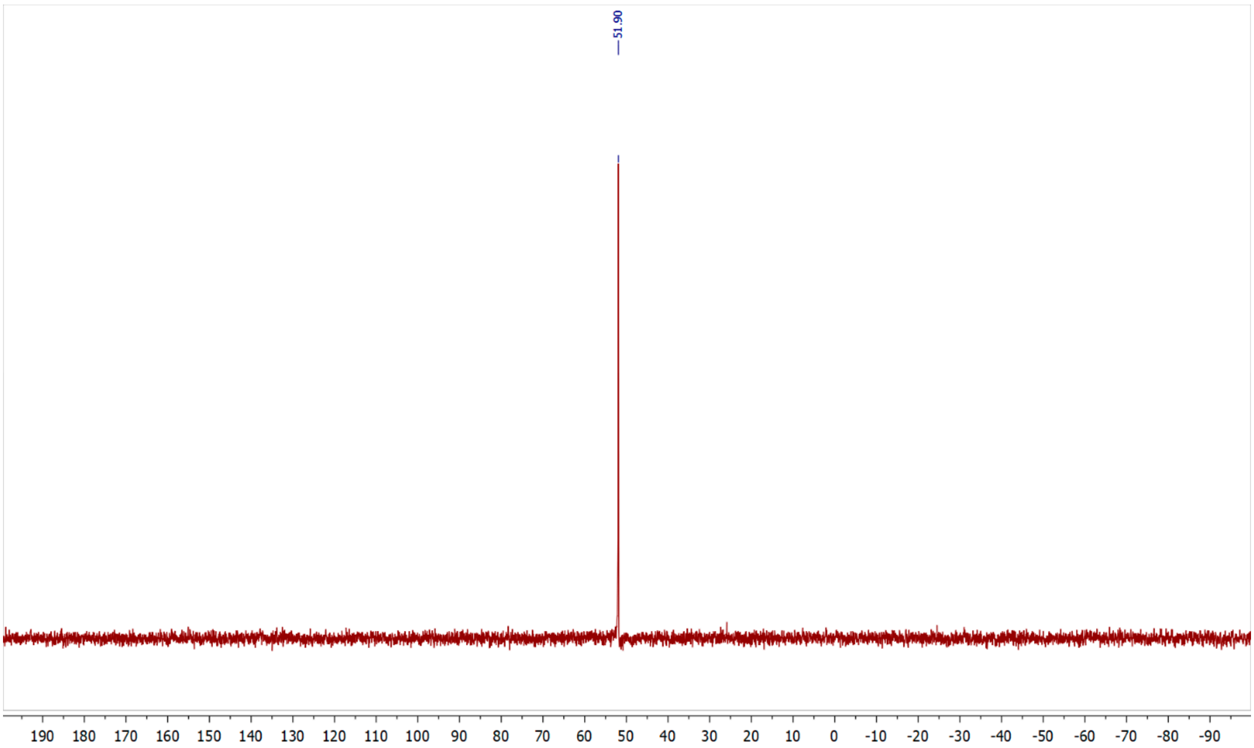

Figure S27. The $^{31}P\{^{1}H\}$ NMR (121.5 MHz, $CDCl_3$) of tri-*tert*-butyl(2-methoxy-2-oxoethyl)phosphonium bromide (**9**).

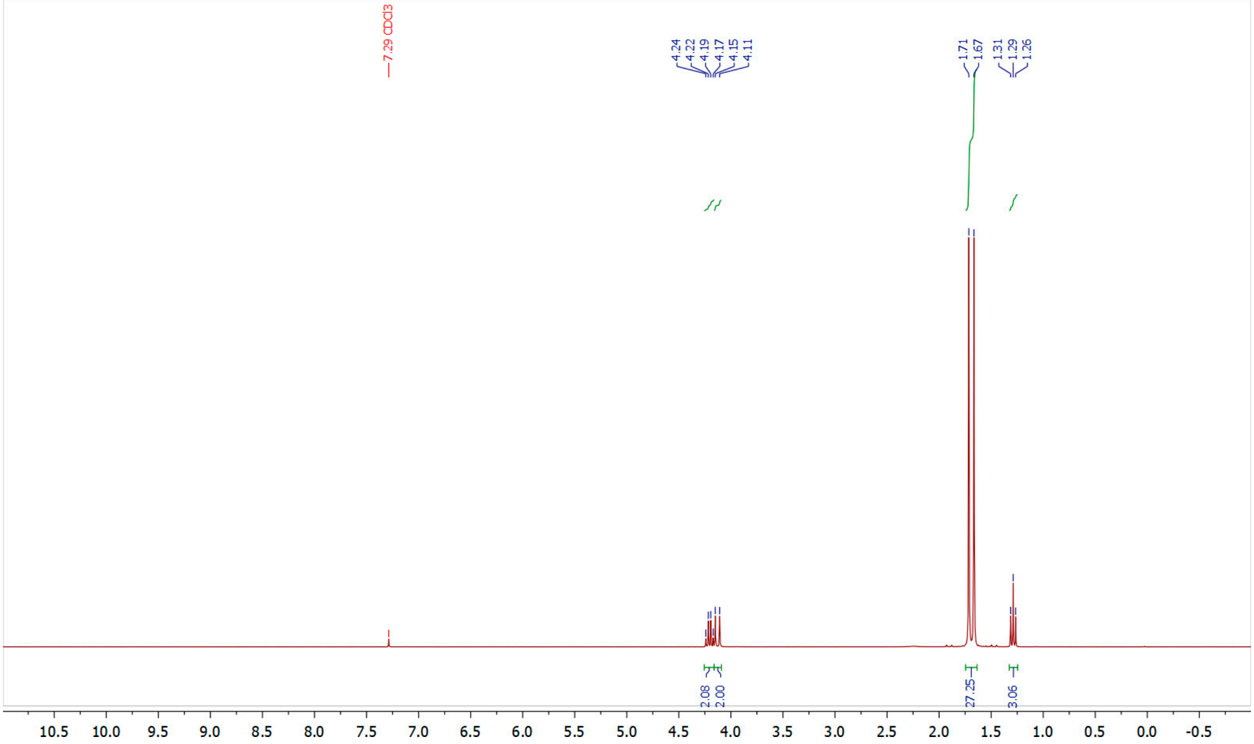

Figure S28. The ^{1}H NMR (300 MHz, $CDCl_3$) tri-*tert*-butyl(2-ethoxy-2-oxoethyl)phosphonium bromide (**10**).

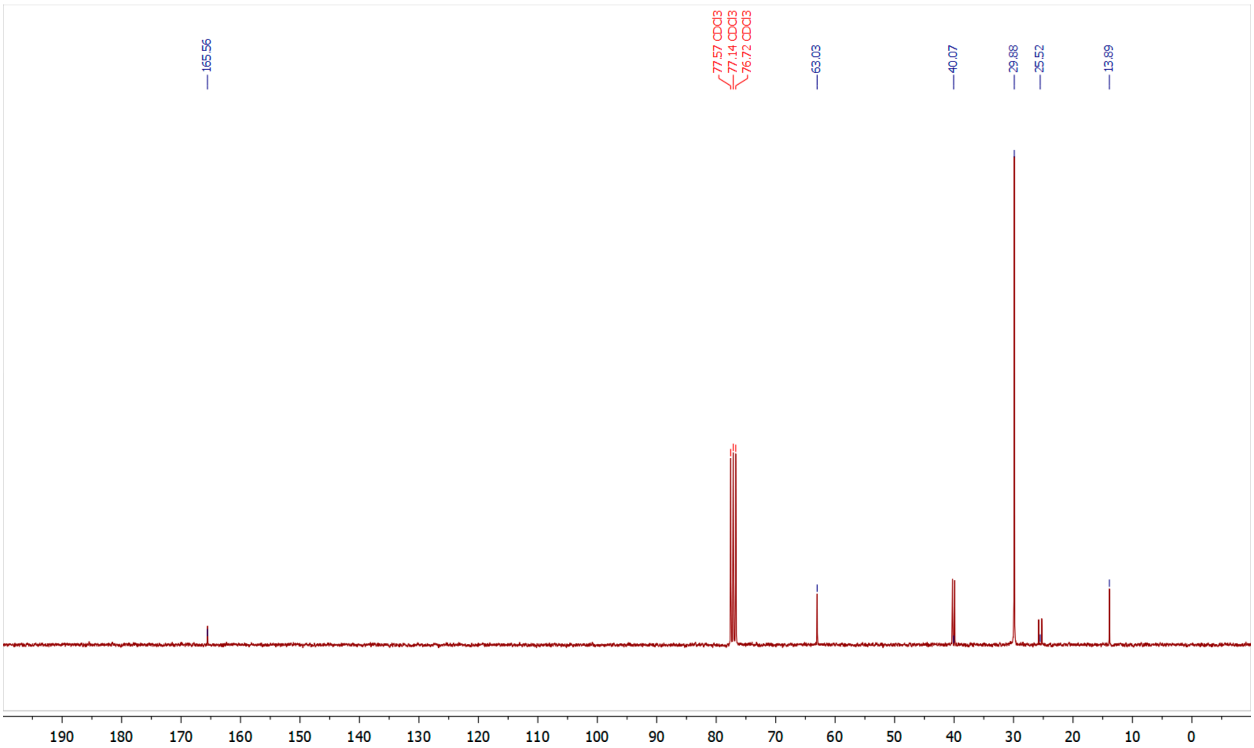

Figure S29. The ^{13}C{^{1}H} NMR (75 MHz, $CDCl_3$) of tri-*tert*-butyl(2-ethoxy-2-oxoethyl)phosphonium bromide (**10**).

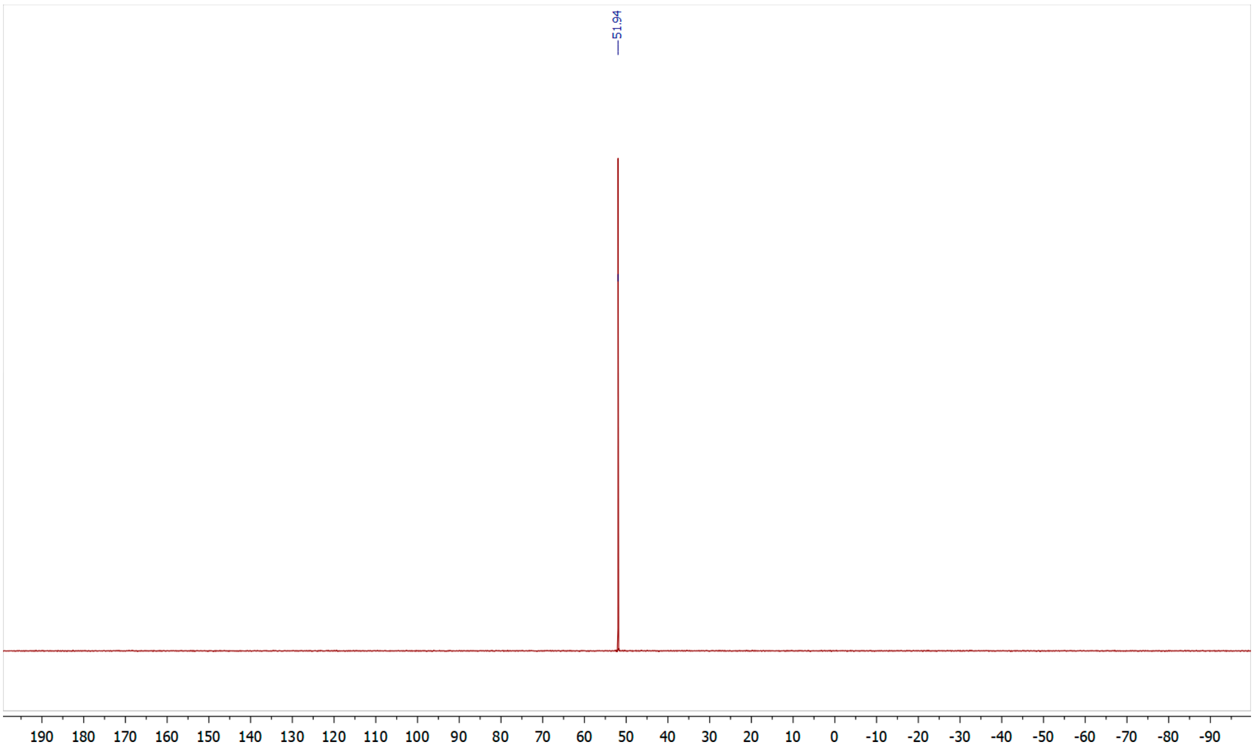

Figure S30. The $^{31}P\{^{1}H\}$ NMR (121.5 MHz, $CDCl_3$) of tri-*tert*-butyl(2-ethoxy-2-oxoethyl)phosphonium bromide (**10**).

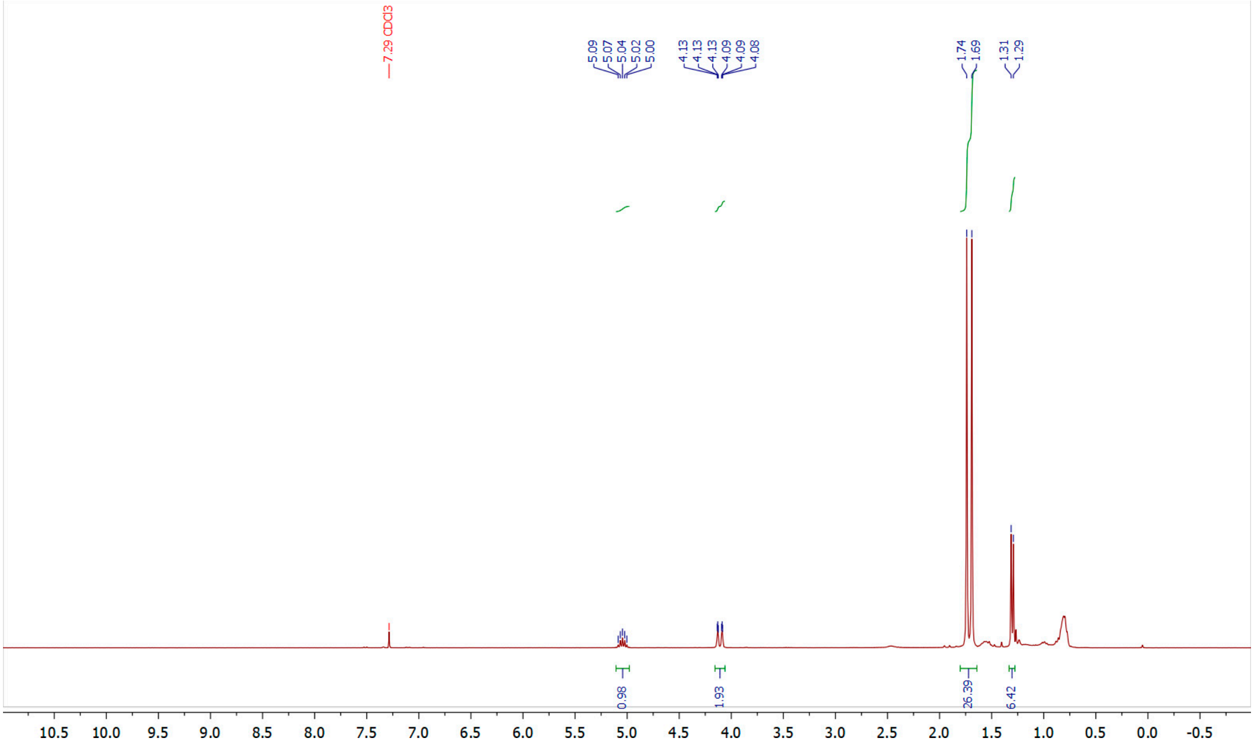

Figure S31. The ^{1}H NMR (300 MHz, $CDCl_3$) tri-*tert*-butyl(2-*iso*-propoxy-2-oxoethyl)phosphonium bromide (**11**).

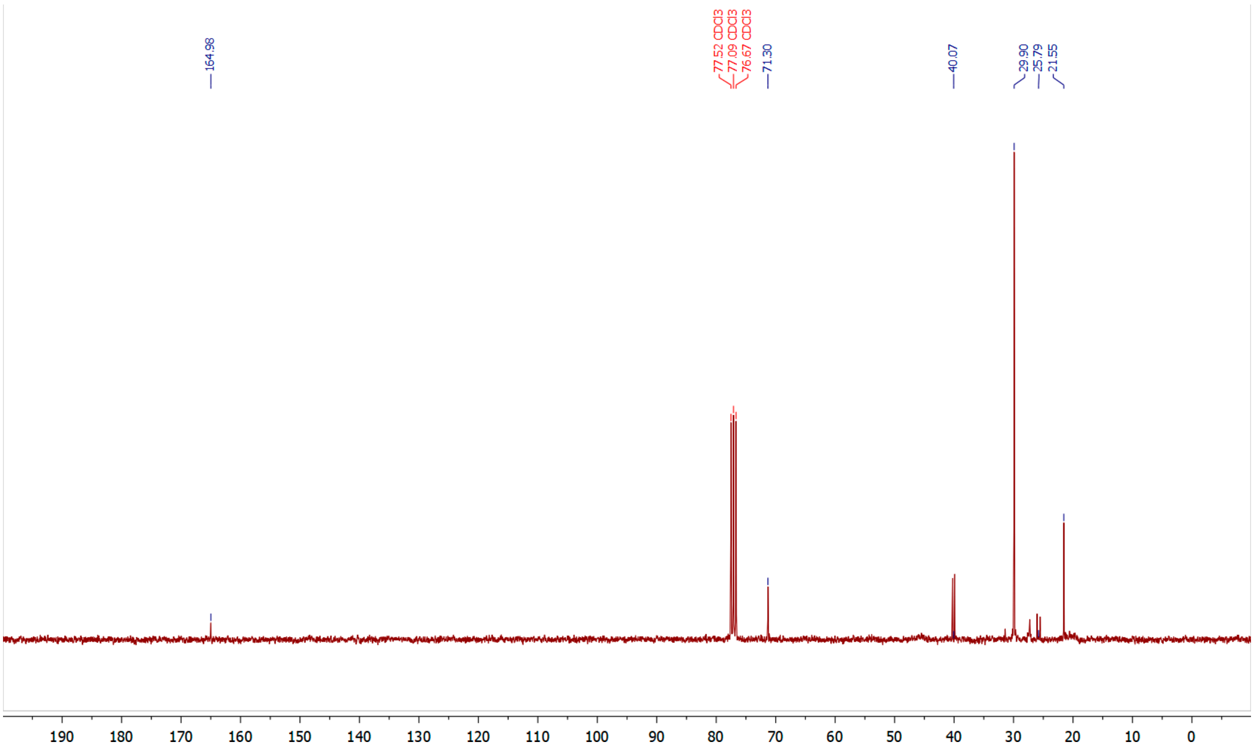

Figure S32. The $^{13}C\{^1H\}$ NMR (75 MHz, $CDCl_3$) of tri-*tert*-butyl(2-*iso*-propoxy-2-oxoethyl)phosphonium bromide (**11**).

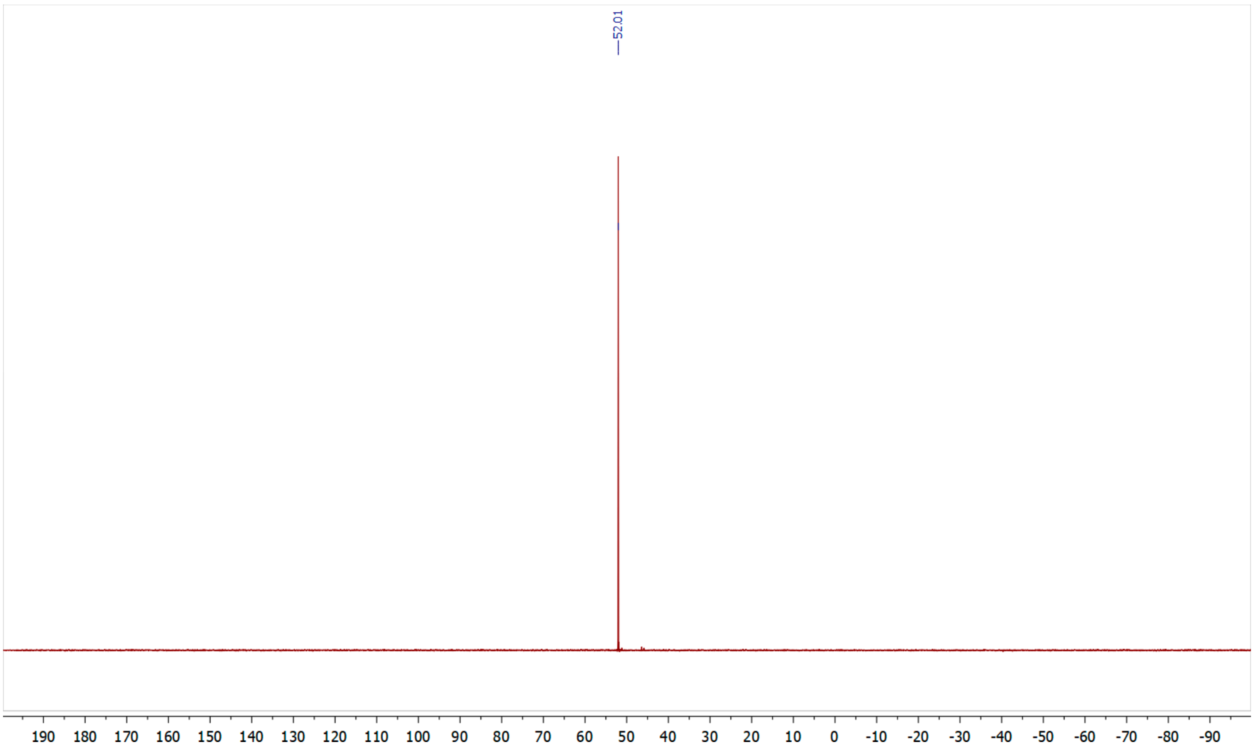

Figure S33. The $^{31}P\{^1H\}$ NMR (121.5 MHz, $CDCl_3$) of tri-*tert*-butyl(2-*iso*-propoxy-2-oxoethyl)phosphonium bromide (**11**).

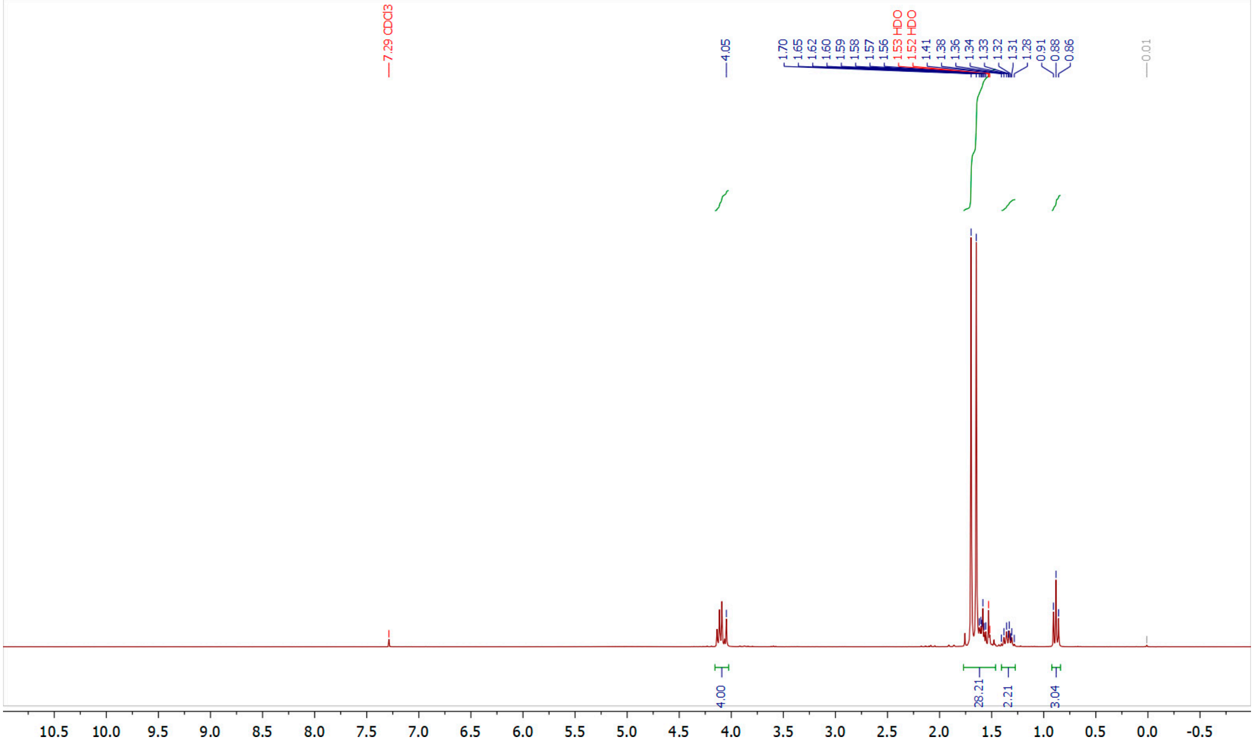

Figure S34. The ^{1}H NMR (300 MHz, $CDCl_3$) tri-*tert*-butyl(2-butoxy-2-oxoethyl)phosphonium bromide (**12**).

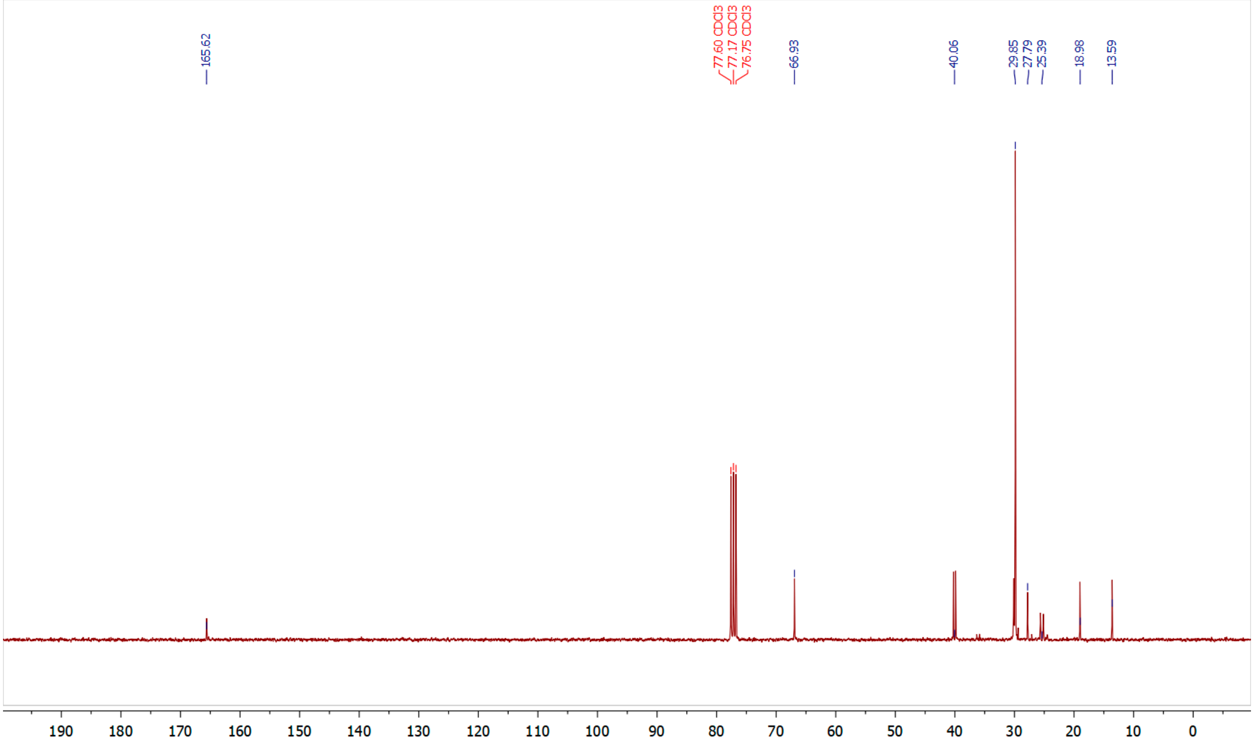

Figure S35. The ^{13}C{^{1}H} NMR (75 MHz, $CDCl_3$) of tri-*tert*-butyl(2-butoxy-2-oxoethyl)phosphonium bromide (**12**).

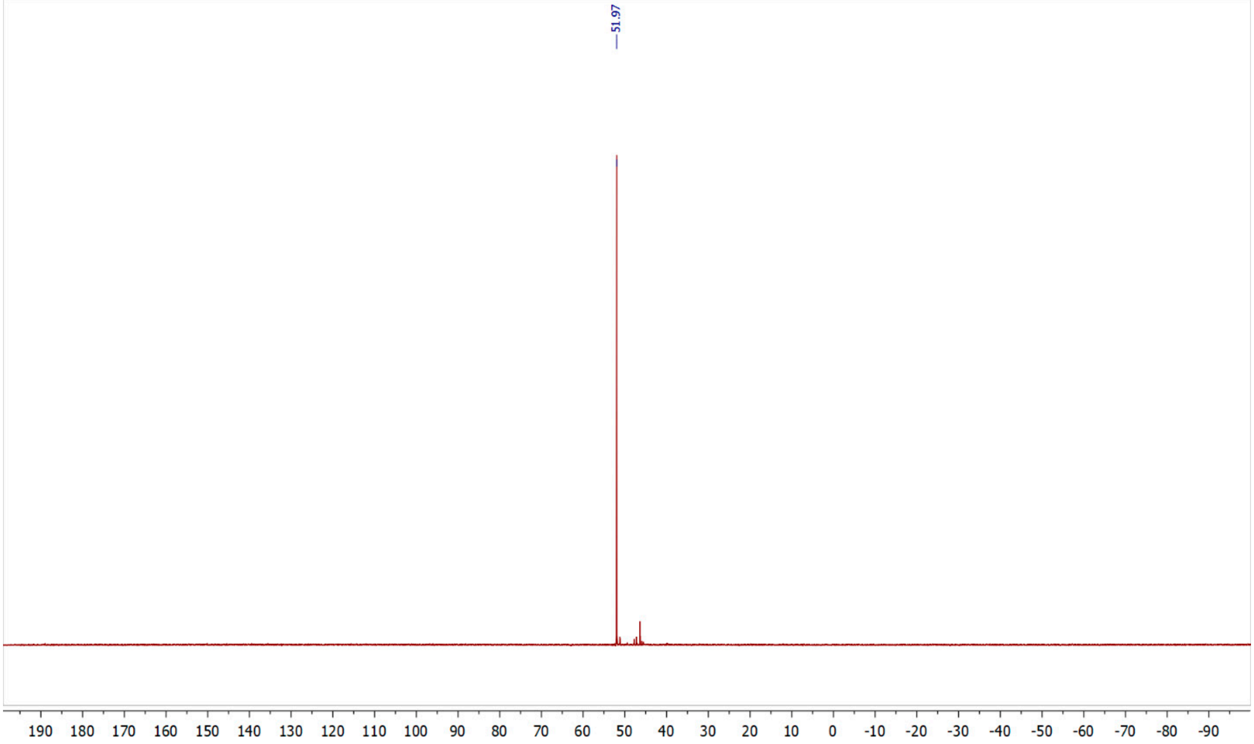

Figure S36. The $^{31}P\{^{1}H\}$ NMR (121.5 MHz, $CDCl_3$) of tri-*tert*-butyl(2-butoxy-2-oxoethyl)phosphonium bromide (**12**).

IR spectra

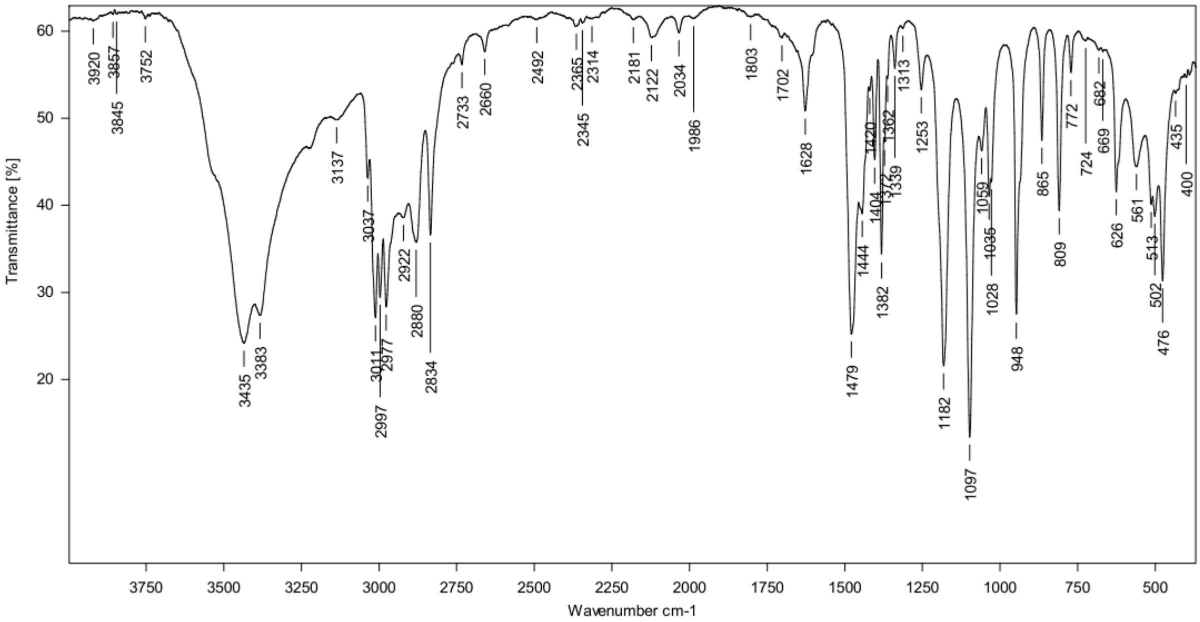

Figure S37. The IR spectra of tri-*tert*-butyl(methoxymethyl)phosphonium chloride (**1**).

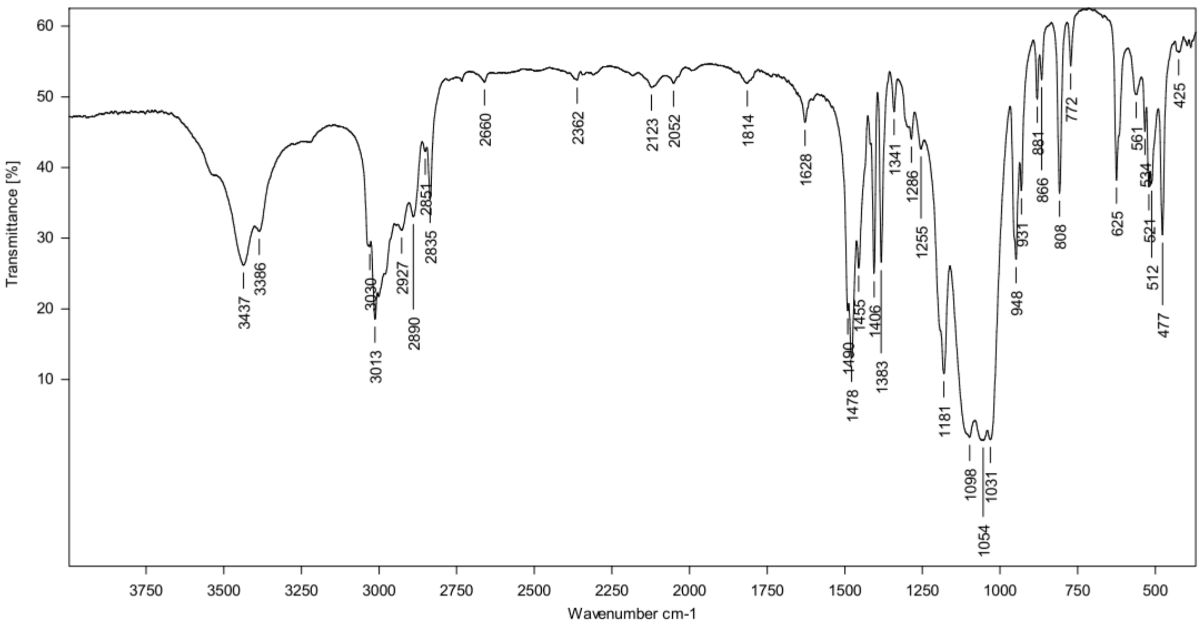

Figure S38. The IR spectra of tri-*tert*-butyl(methoxymethyl)phosphonium tetrafluoroborate (**2**).

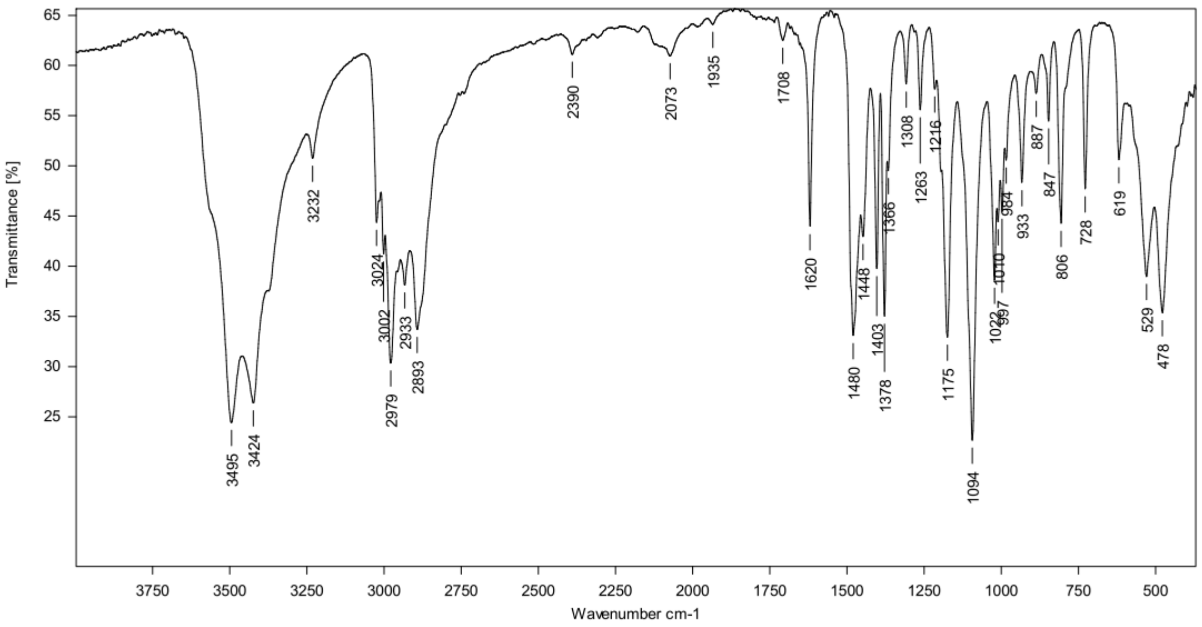

Figure S39. The IR spectra of tri-*tert*-butyl(2-ethoxyethyl)phosphonium bromide (**3**).

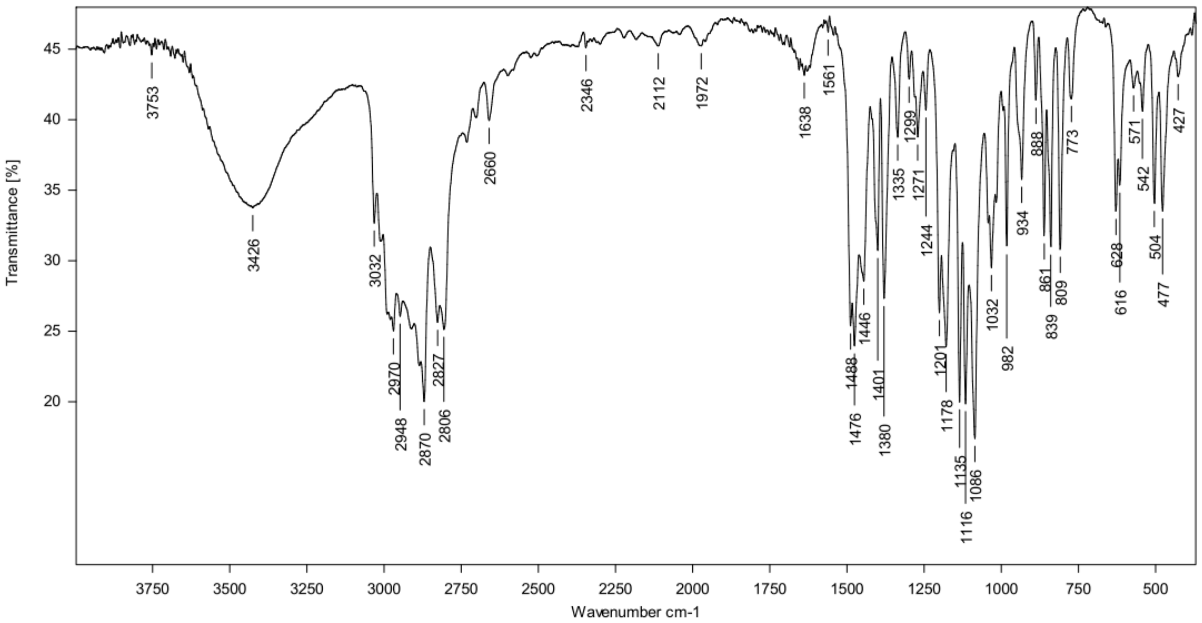

Figure S40. The IR spectra of tri-*tert*-butyl((2-methoxyethoxy)methyl)phosphonium chloride (**4**).

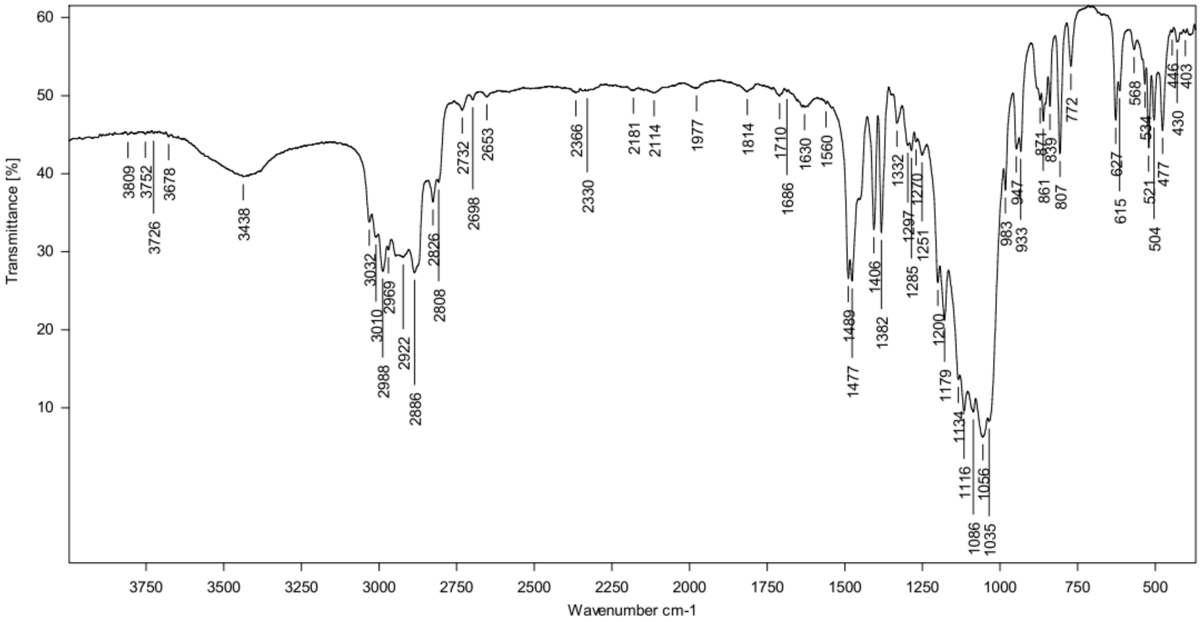

Figure S41. The IR spectra of tri-*tert*-butyl((2-methoxyethoxy)methyl)phosphonium tetrafluoroborate (**5**).

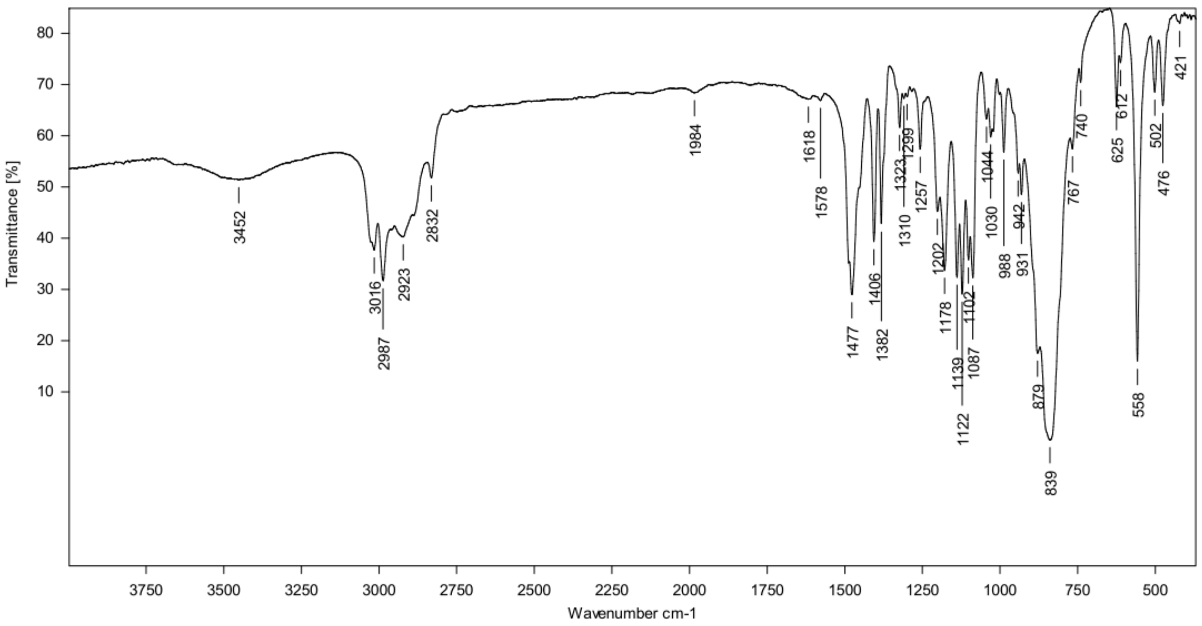

Figure S42. The IR spectra of tri-*tert*-butyl((2-methoxyethoxy)methyl)phosphonium hexafluorophosphate (**6**).

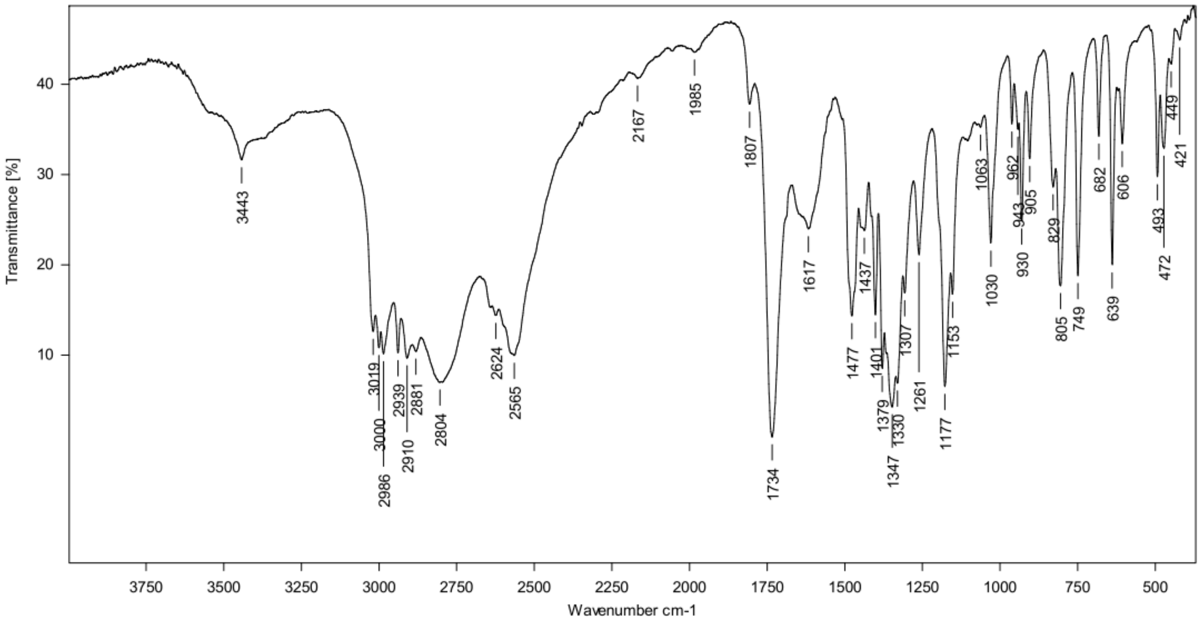

Figure S43. The IR spectra of tri-*tert*-butyl(2-carboxyethyl)phosphonium bromide (**7**).

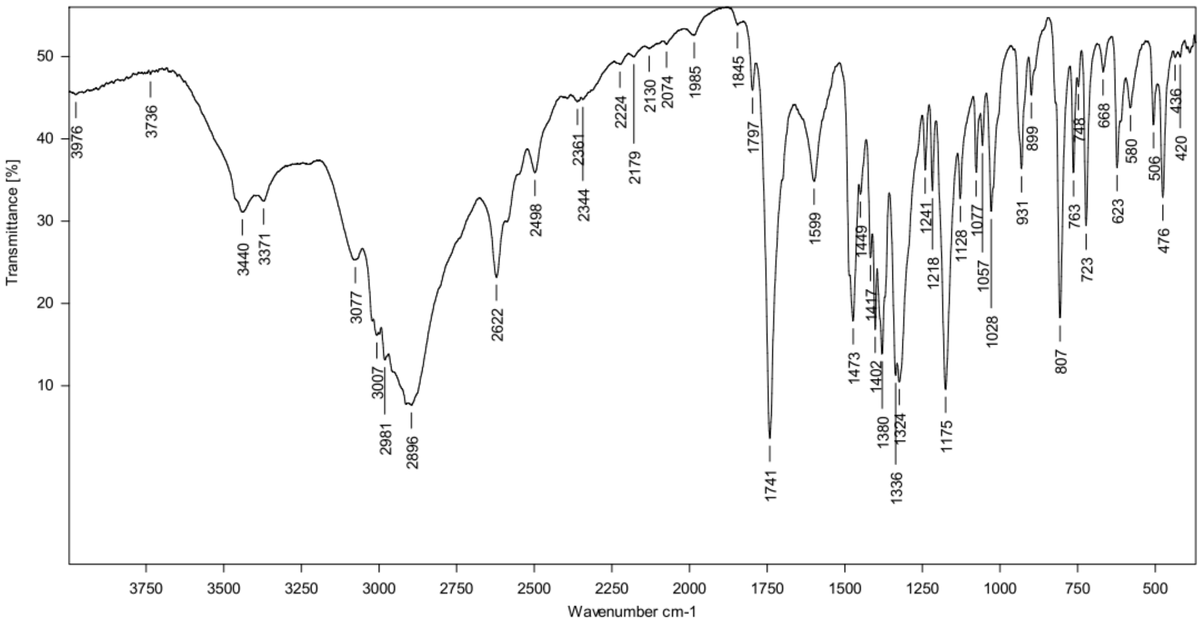

Figure S44. The IR spectra of tri-*tert*-butyl(5-carboxypentyl)phosphonium bromide (**8**).

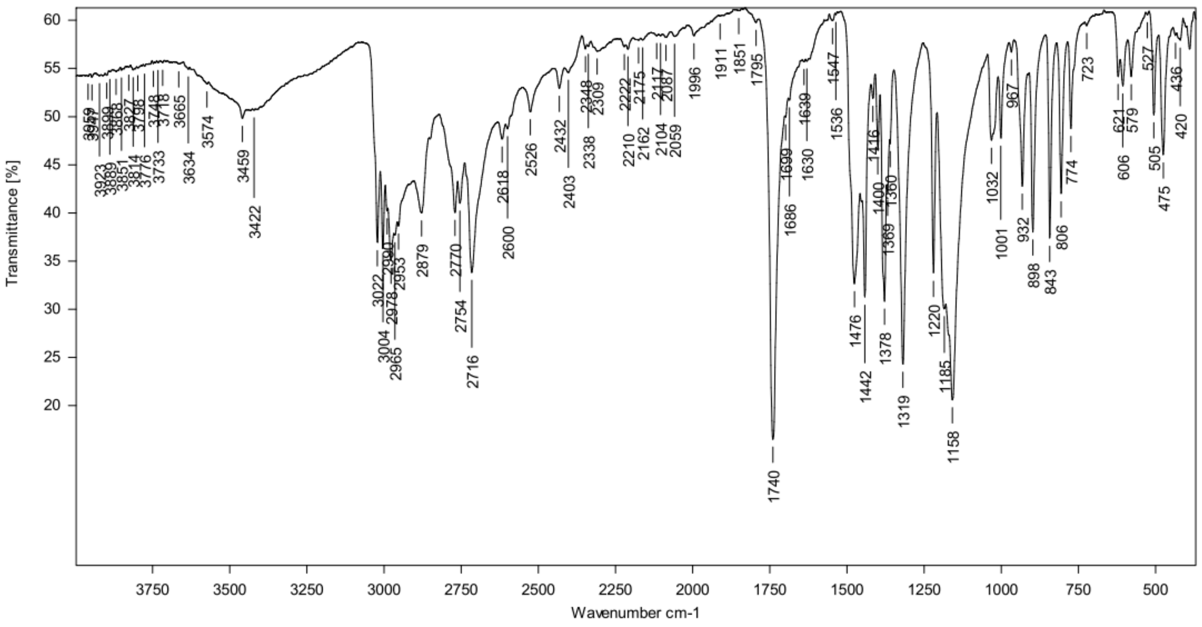

Figure S45. The IR spectra of tri-*tert*-butyl(2-methoxy-2-oxoethyl)phosphonium bromide (**9**).

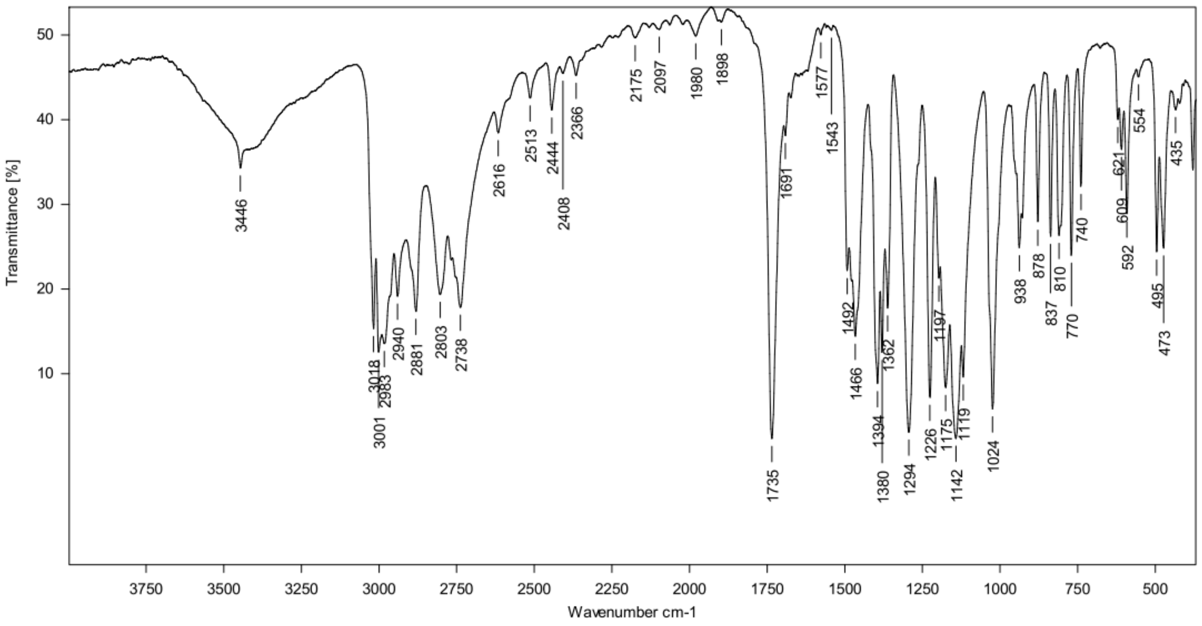

Figure S46. The IR spectra of tri-*tert*-butyl(2-ethoxy-2-oxoethyl)phosphonium bromide (**10**).

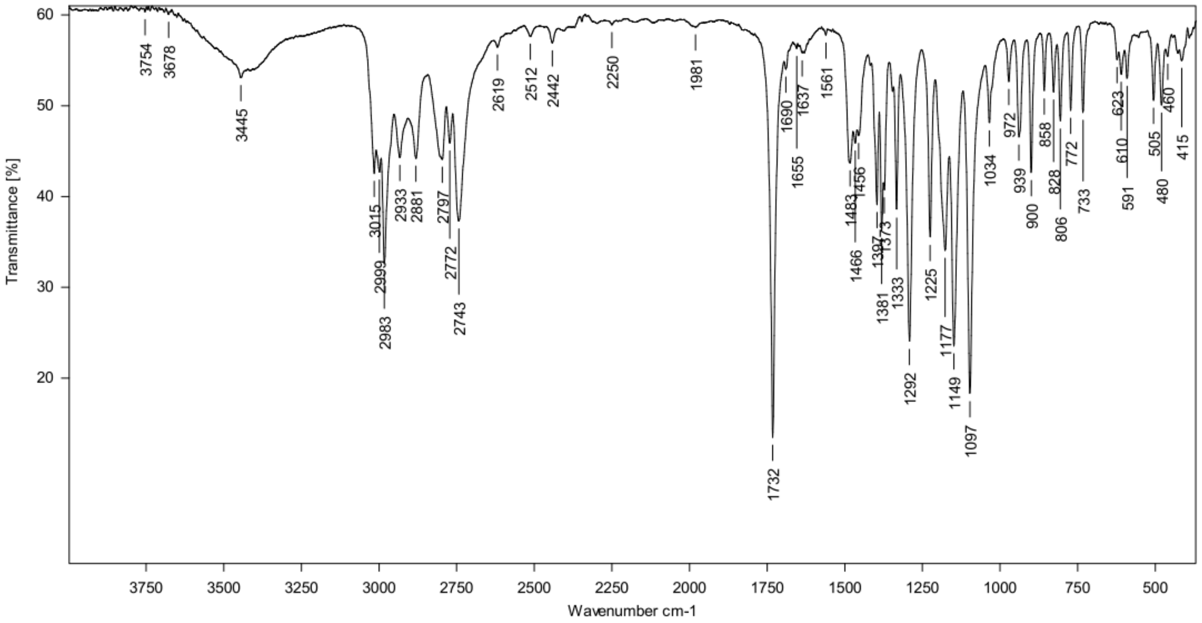

Figure S47. The IR spectra of tri-*tert*-butyl(2-*iso*-propoxy-2-oxoethyl)phosphonium bromide (**11**).

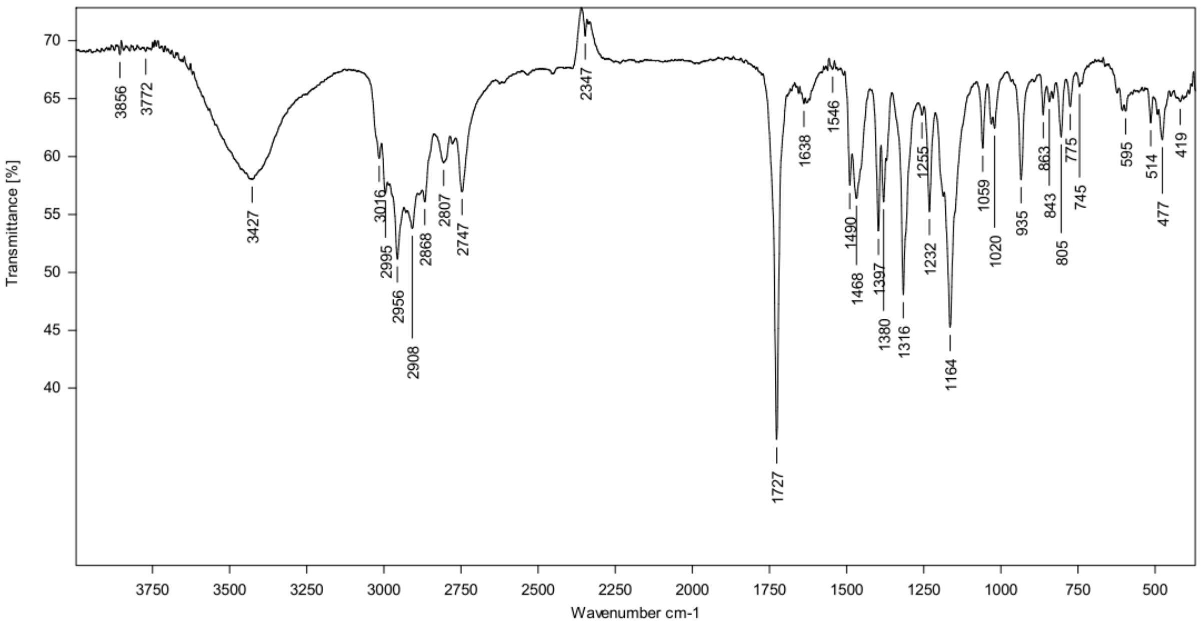

Figure S48. The IR spectra of tri-tert-butyl(2-butoxy-2-oxoethyl)phosphonium bromide (**12**).

TG-DSC curves

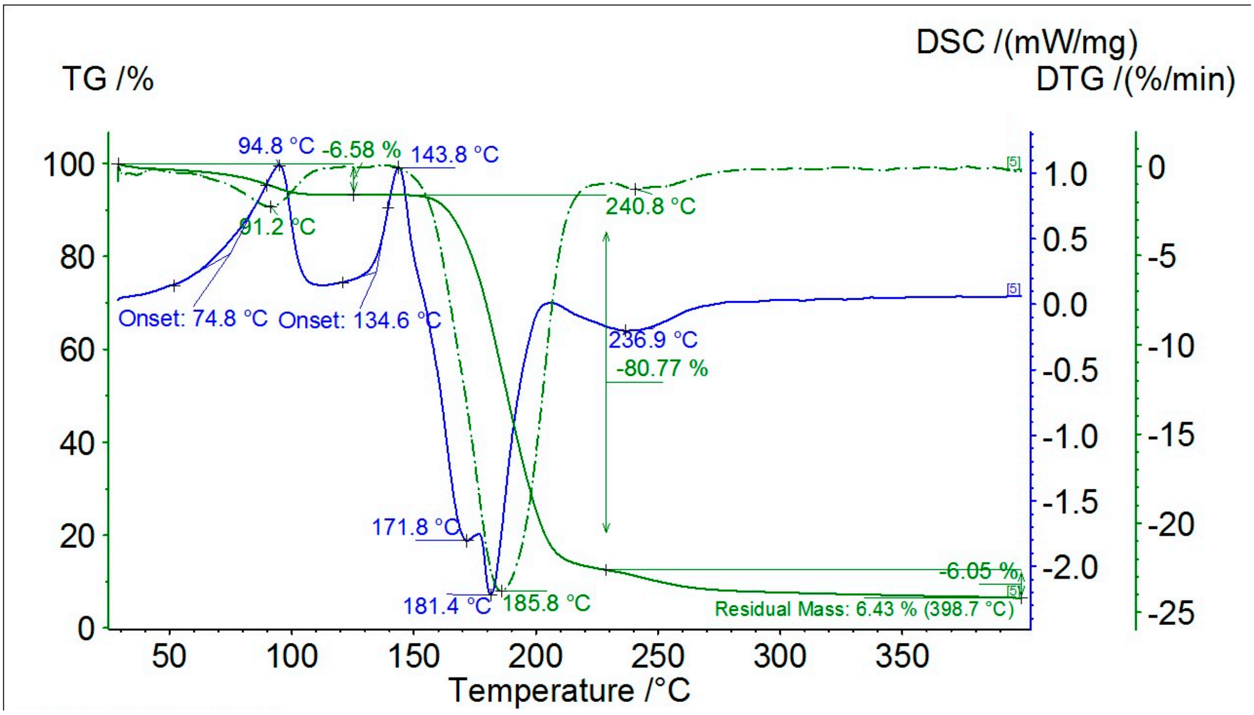

Figure S49. TG-DSC curves for **1** (tri-*tert*-butyl(methoxymethyl)phosphonium chloride): TG curve (green line), DSC curve (blue line), DTG curve (green dashed line).

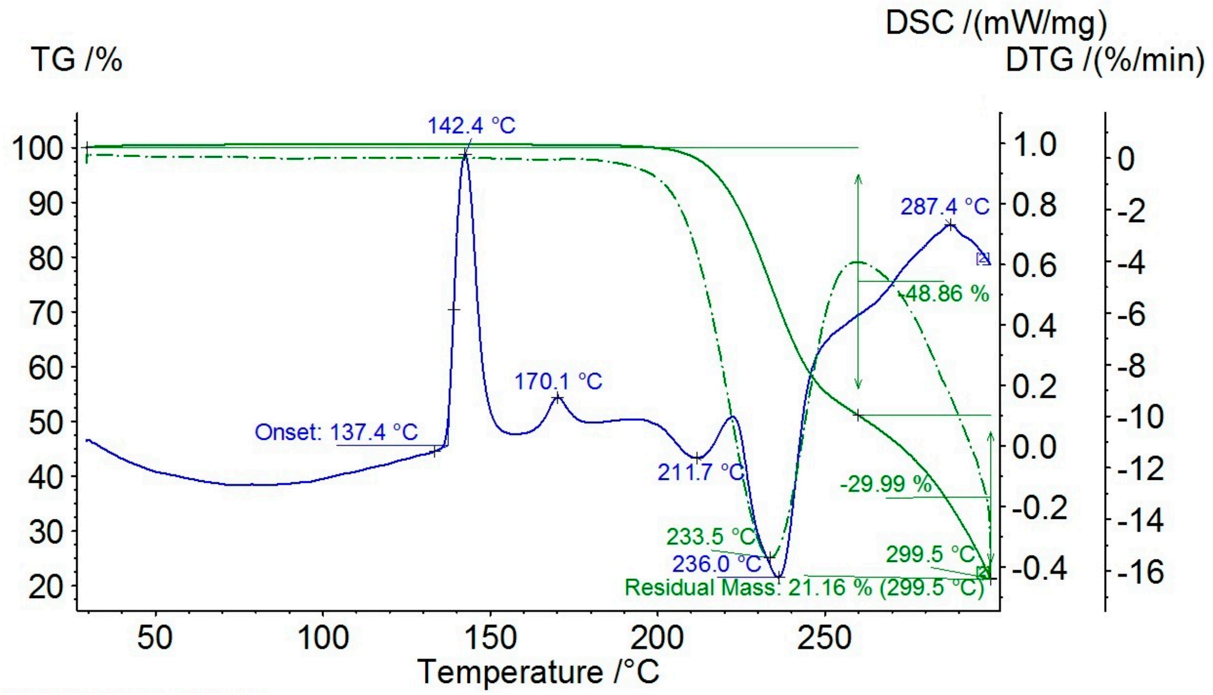

Figure S50. TG-DSC curves for **2** (tri-*tert*-butyl(methoxymethyl)phosphonium tetrafluoroborate): TG curve (green line), DSC curve (blue line), DTG curve (green dashed line).

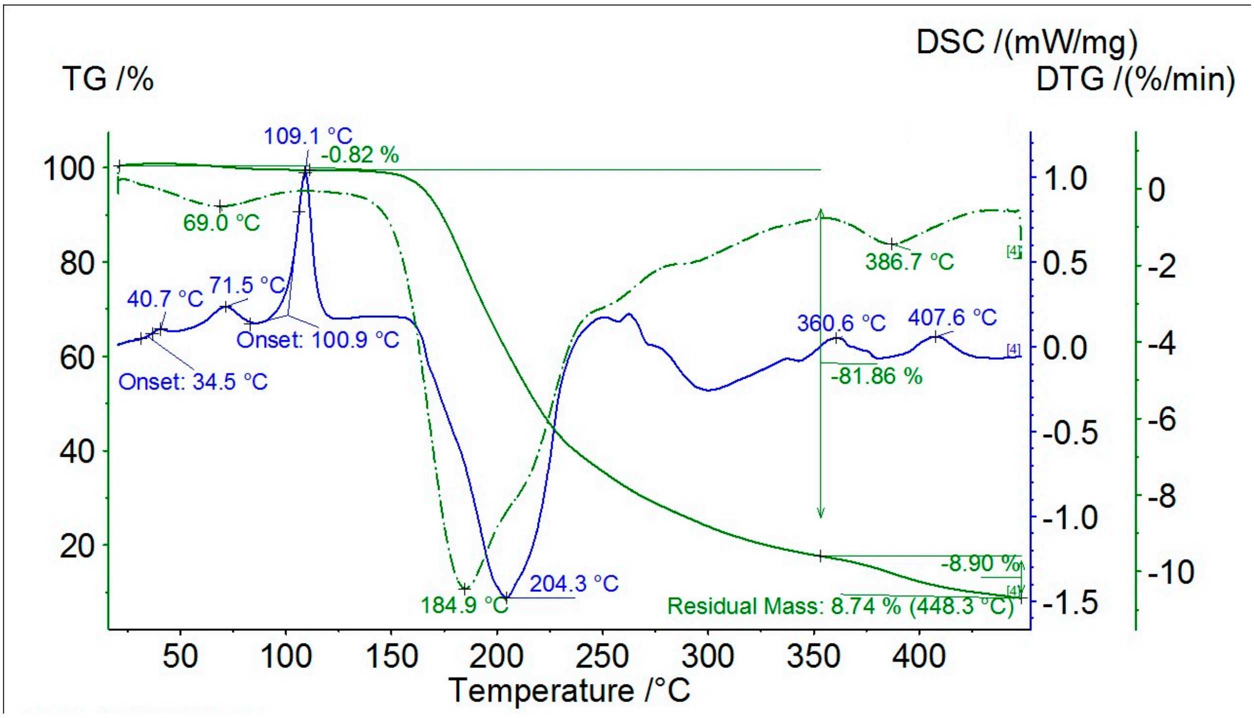

Figure S51. TG-DSC curves for **3** (tri-*tert*-butyl(2-ethoxyethyl)phosphonium bromide): TG curve (green line), DSC curve (blue line), DTG curve (green dashed line).

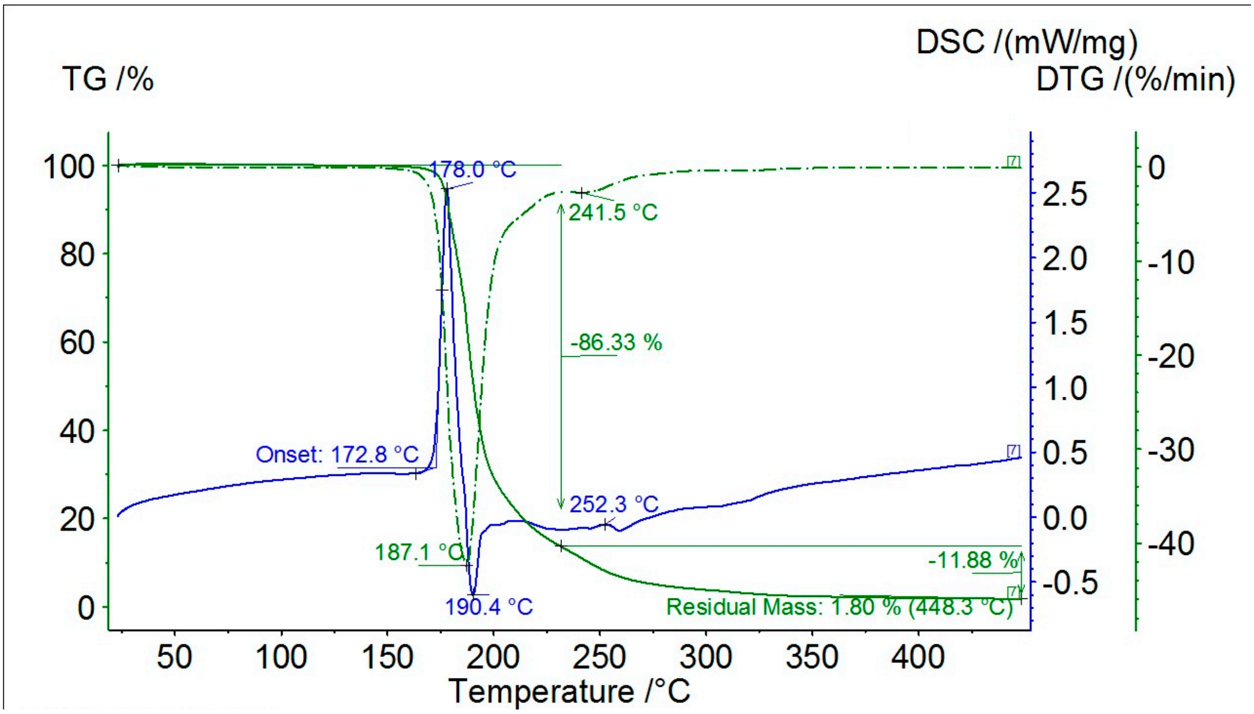

Figure S52. TG-DSC curves for **4** (tri-*tert*-butyl(MEM)phosphonium chloride): TG curve (green line), DSC curve (blue line), DTG curve (green dashed line).

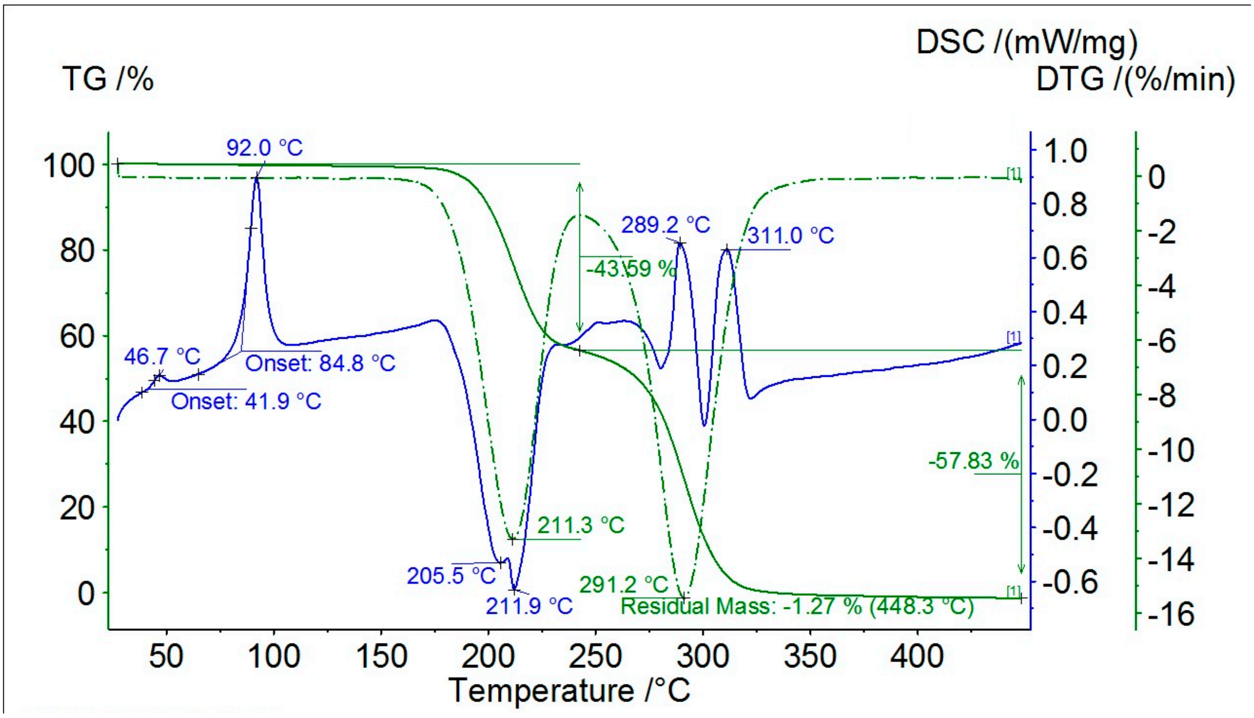

Figure S53. TG-DSC curves for **5** (tri-*tert*-butyl(MEM)phosphonium tetrafluoroborate): TG curve (green line), DSC curve (blue line), DTG curve (green dashed line).

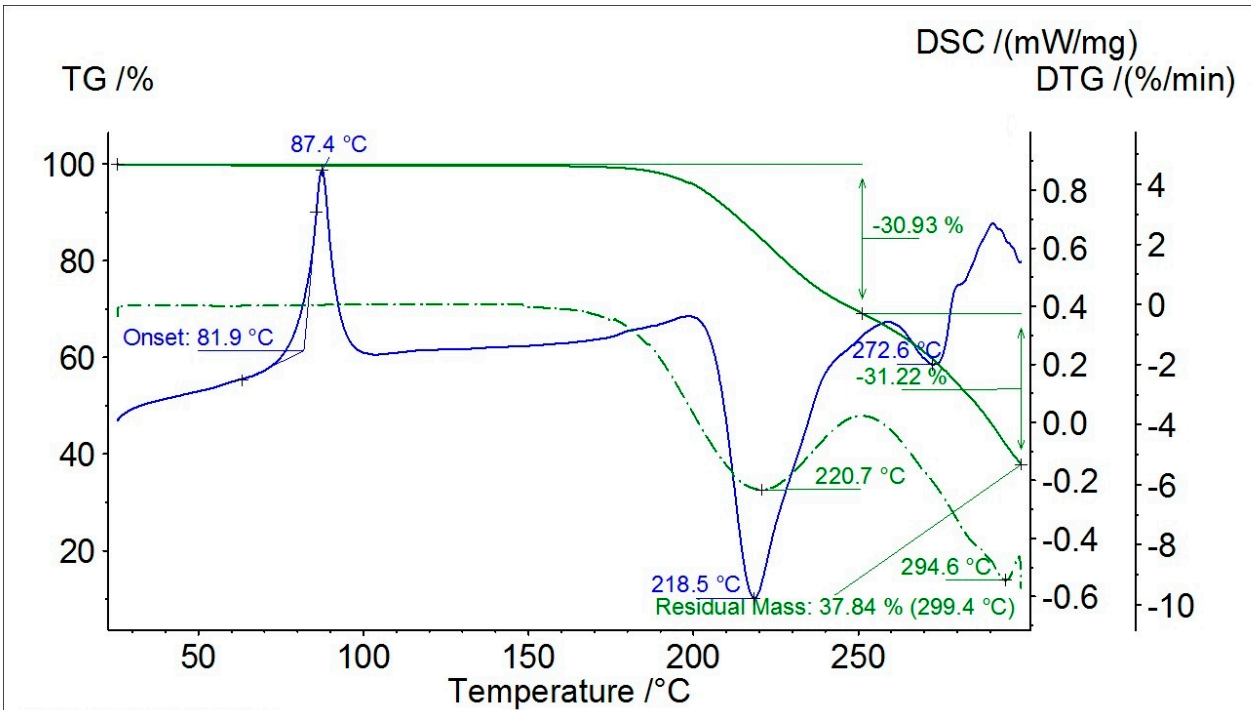

Figure S54. TG-DSC curves for **6** (tri-*tert*-butyl(MEM)phosphonium hexafluorophosphate): TG curve (green line), DSC curve (blue line), DTG curve (green dashed line).

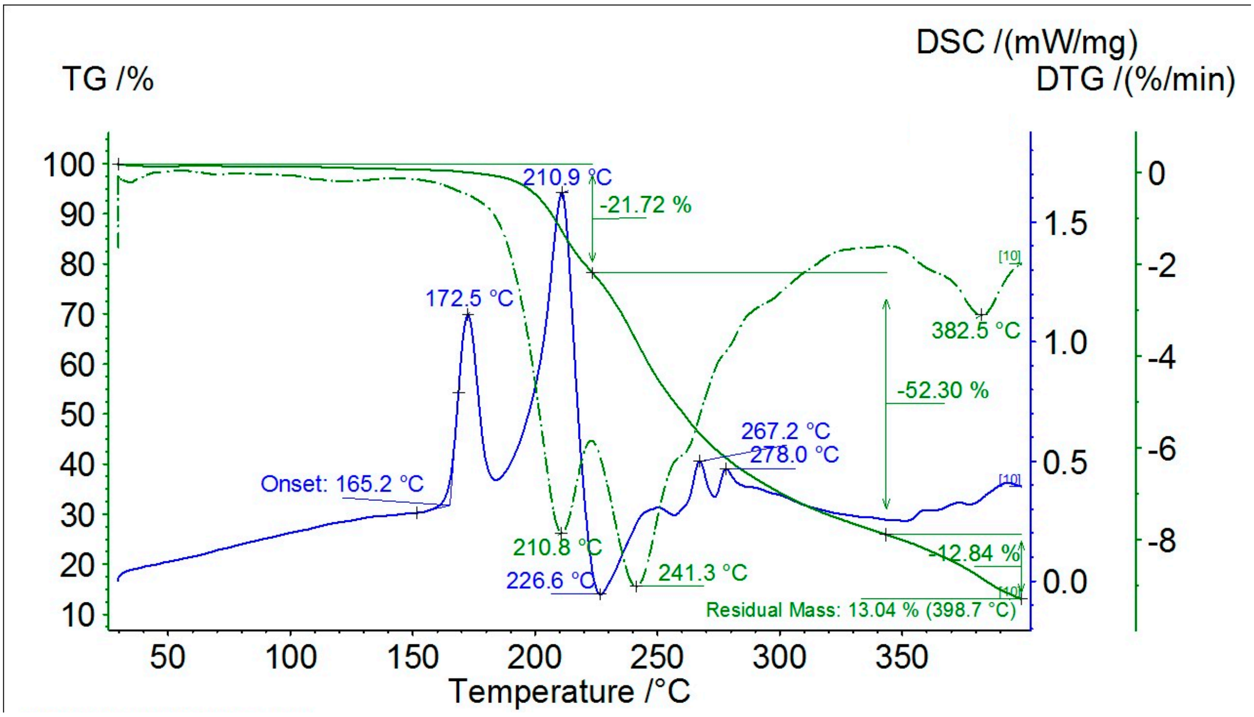

Figure S55. TG-DSC curves for **7** (tri-*tert*-butyl(2-carboxyethyl)phosphonium bromide): TG curve (green line), DSC curve (blue line), DTG curve (green dashed line).

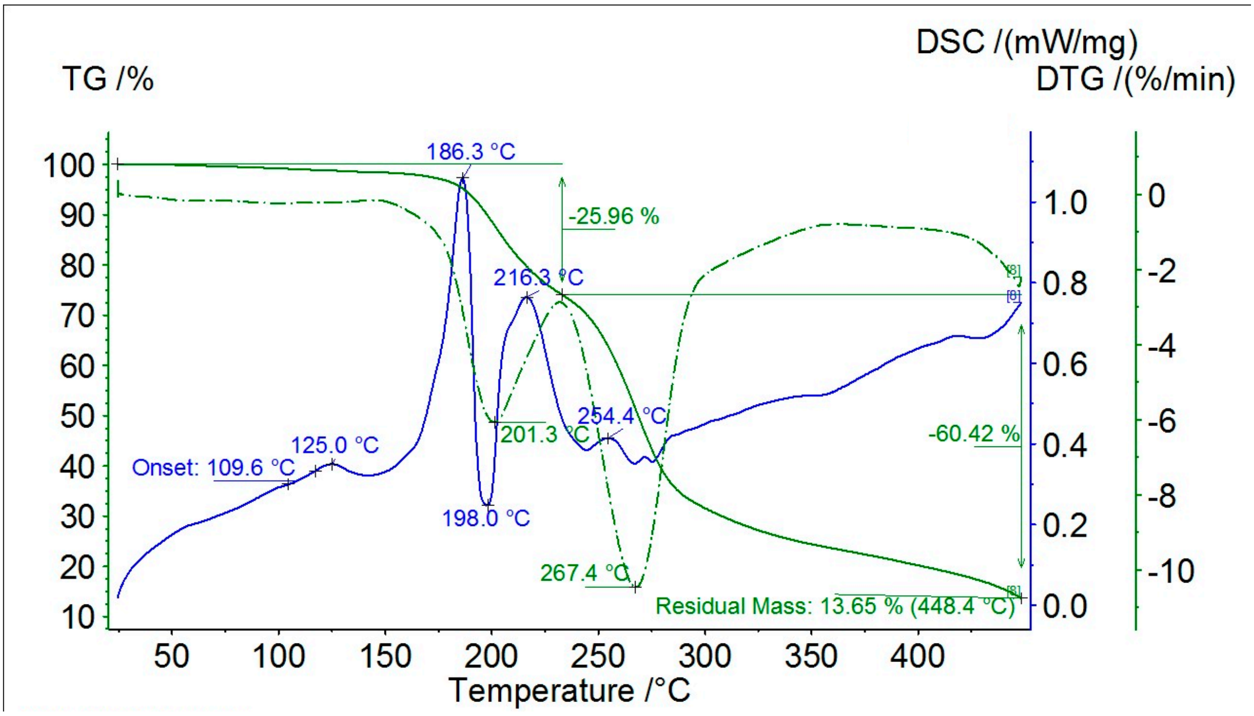

Figure S56. TG-DSC curves for **8** (tri-*tert*-butyl(5-carboxypentyl)phosphonium bromide): TG curve (green line), DSC curve (blue line), DTG curve (green dashed line).

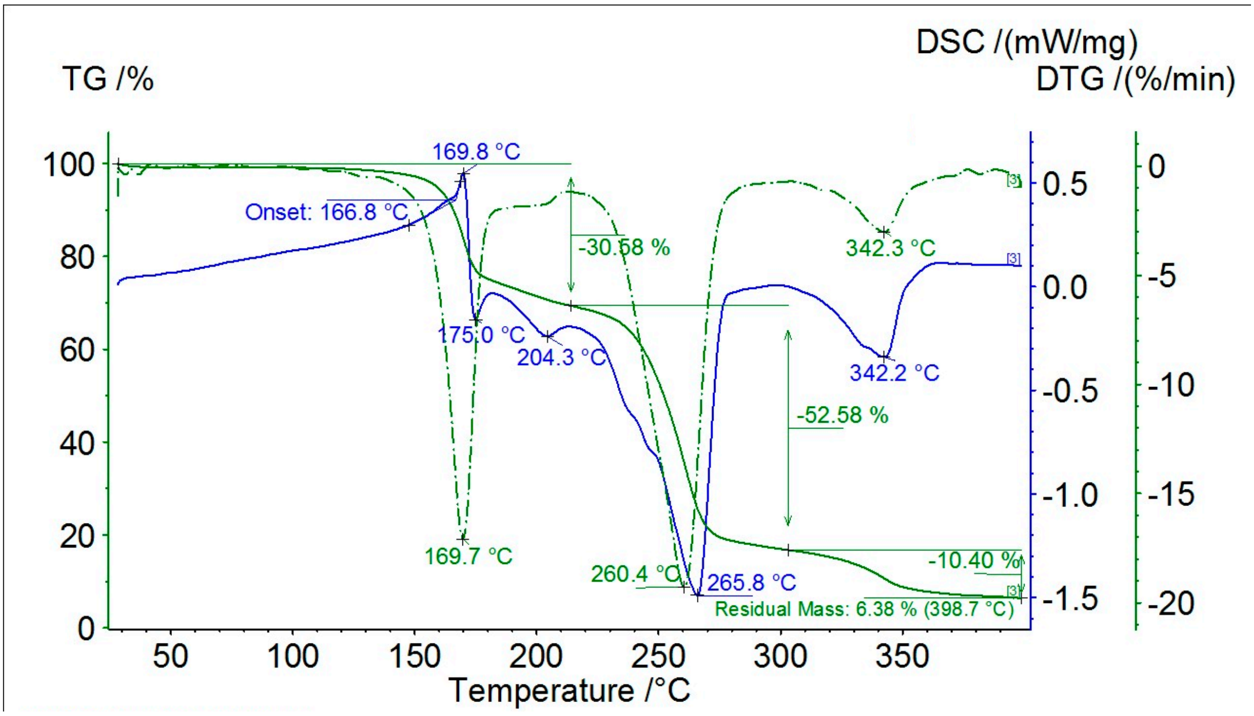

Figure S57. TG-DSC curves for **9** (tri-*tert*-butyl(2-methoxy-2-oxoethyl)phosphonium bromide): TG curve (green line), DSC curve (blue line), DTG curve (green dashed line).

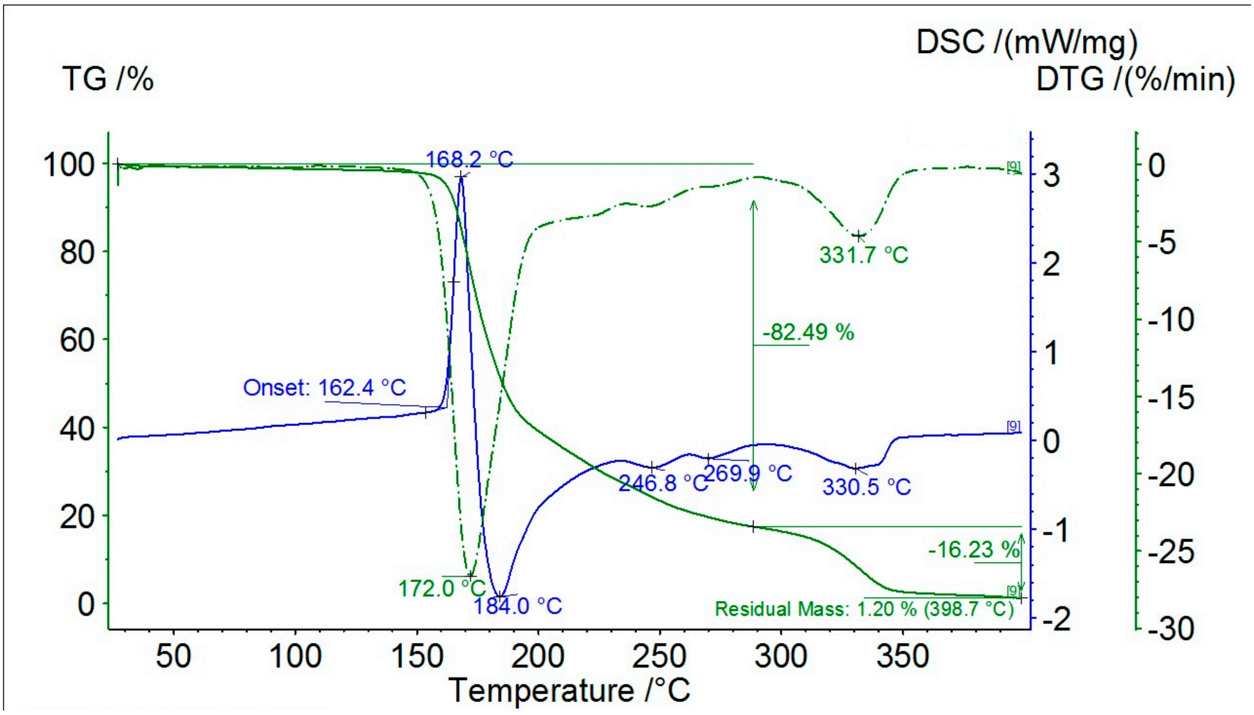

Figure S58. TG-DSC curves for **10** (tri-*tert*-butyl(2-ethoxy-2-oxoethyl)phosphonium bromide): TG curve (green line), DSC curve (blue line), DTG curve (green dashed line).

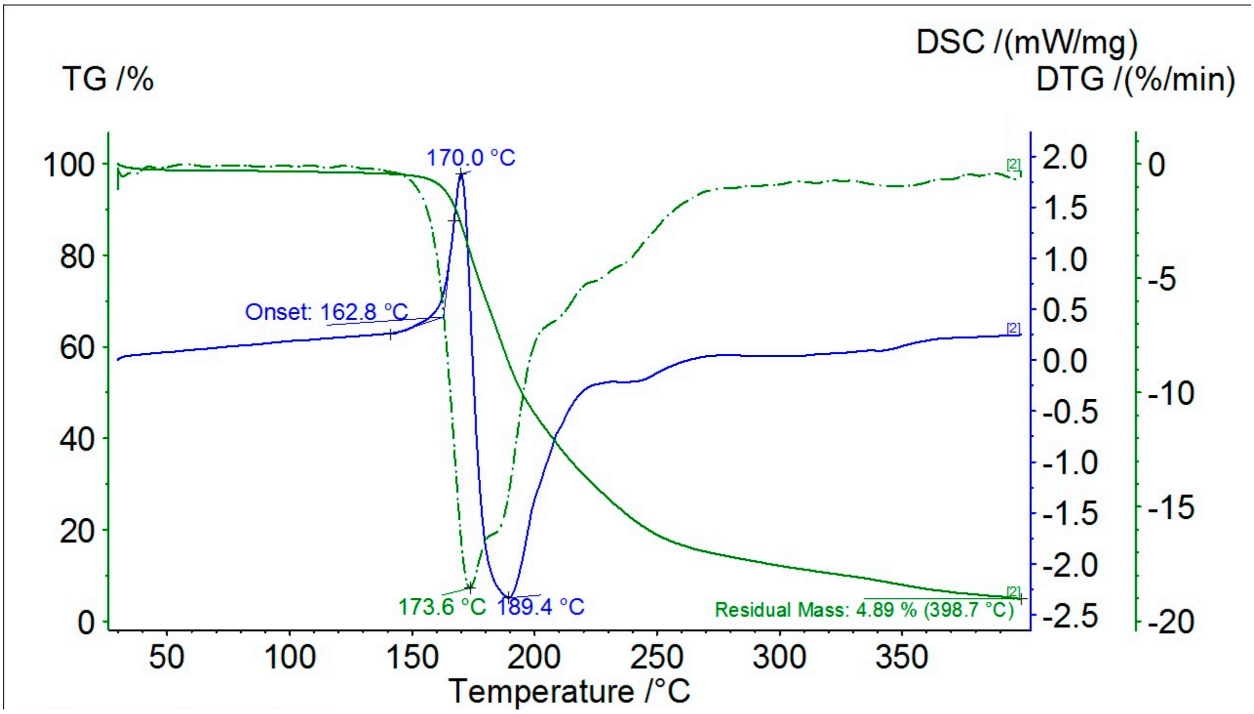

Figure S59. TG-DSC curves for **11** (tri-*tert*-butyl(2-*iso*-propoxy-2-oxoethyl)phosphonium bromide): TG curve (green line), DSC curve (blue line), DTG curve (green dashed line).

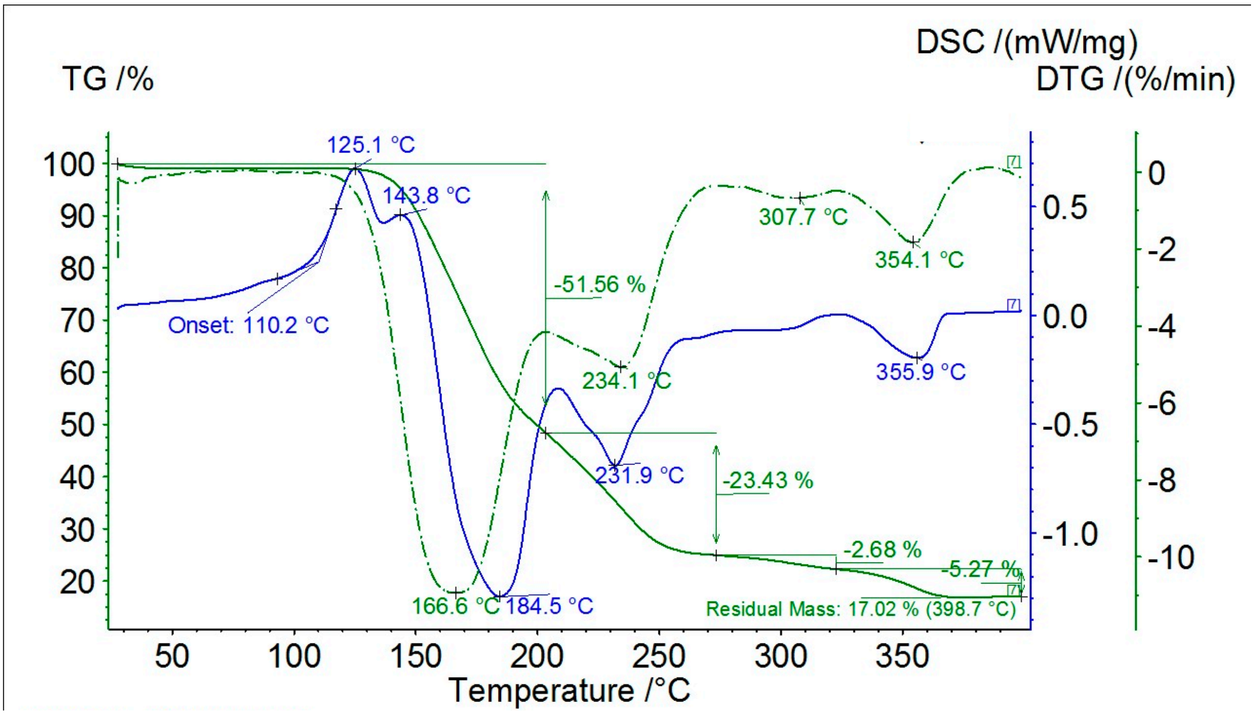

Figure S60. TG-DSC curves for **12** (tri-*tert*-butyl(2-butoxy-2-oxoethyl)phosphonium bromide): TG curve (green line), DSC curve (blue line), DTG curve (green dashed line).

X-Ray data

Crystal data for **2**, $C_{14}H_{32}BF_4OP$ (*M* = 334.17 g/mol): monoclinic, space group *P*2$_1$/n (no. 14), *a* = 8.25375(4) Å, *b* = 14.46286(8) Å, *c* = 15.07244(9) Å, *β* = 95.1251(5)°, *V* = 1792.046(17) Å^3, Z = 4, T = 100 K, *μ*(CuKα) = 1.673 mm^{-1}, *Dcalc* = 1.239 g/cm^3, 46088 reflections measured (4.245° ≤ 2Θ ≤ 77.819°), 3823 unique (R_{int} = 0.0284) which were used in all calculations. The final R_1 was 0.0327 ($I > 2\sigma(I)$) and wR_2 was 0.0854 (all data). CCDC refcode: 2288793.

Crystal data for **4**, $C_{16}H_{36}ClO_2P$ (*M* = 326.87 g/mol): monoclinic, space group *P*2$_1$/c (no. 14), *a* = 7.73708(4) Å, *b* = 14.85477(8) Å, *c* = 16.43637(8) Å, *β* = 97.0552(5)°, *V* = 1874.769(17) Å^3, Z = 4, T = 100 K, *μ*(CuKα) = 2.601 mm^{-1}, *Dcalc* = 1.158 g/cm^3, 41215 reflections measured (4.025° ≤ 2Θ ≤ 77.761°), 3993 unique (R_{int} = 0.0394) which were used in all calculations. The final R_1 was 0.0295 ($I > 2\sigma(I)$) and wR_2 was 0.0769 (all data). CCDC refcode: 2288794.

Crystal data for **6**, $C_{16}H_{36}F_6O_2P_2$ (*M* = 436.39 g/mol): monoclinic, space group *P*2$_1$/c (no. 14), *a* = 8.37685(13) Å, *b* = 21.2978(3) Å, *c* = 12.4934(2) Å, *β* = 108.3892(18)°, *V* = 2115.11(6) Å^3, Z = 4, T = 100 K, *μ*(CuKα) = 2.424 mm^{-1}, *Dcalc* = 1.370 g/cm^3, 28367 reflections measured (4.152° ≤ 2Θ ≤ 77.718°), 4483 unique (R_{int} = 0.0327) which were used in all calculations. The final R_1 was 0.0478 ($I > 2\sigma(I)$) and wR_2 was 0.1263 (all data). CCDC refcode: 2288795.

Crystal data for **7**, $C_{15}H_{32}BrO_2P$ (*M* = 355.28 g/mol): monoclinic, space group *P*2$_1$/n (no. 14), *a* = 8.04309(4) Å, *b* = 16.04588(7) Å, *c* = 13.84738(6) Å, *β* = 101.3561(5)°, *V* = 1752.134(14) Å^3, Z = 4, T = 100 K, *μ*(CuKα) = 4.031 mm^{-1}, *Dcalc* = 1.347 g/cm^3, 45812 reflections measured (4.266° ≤ 2Θ ≤ 77.729°), 3736 unique (R_{int} = 0.0297) which were used in all calculations. The final R_1 was 0.0245 ($I > 2\sigma(I)$) and wR_2 was 0.0644 (all data). CCDC refcode: 2288796.

Crystal data for **8**, $C_{18}H_{38}BrO_2P$ (*M* = 397.36 g/mol): monoclinic, space group *P*2$_1$/c (no. 14), *a* = 8.12754(6) Å, *b* = 24.5527(2) Å, *c* = 10.34299(7) Å, *β* = 97.3579(7)°, *V* = 2046.98(3) Å^3, Z = 4, T = 100 K, μ(CuKα) = 3.504 mm^{-1}, *Dcalc* = 1.289 g/cm^3, 51939 reflections measured (3.600° ≤ 2Θ ≤ 77.793°), 4358 unique (R_{int} = 0.0403) which were used in all calculations. The final R_1 was 0.0361 ($I > 2\sigma(I)$) and wR_2 was 0.0950 (all data). CCDC refcode: 2288797

Crystal data for **9**, $C_{15}H_{32}BrO_2P$ (*M* = 355.28 g/mol): orthorhombic, space group *P*bcn (no. 60), *a* = 12.67842(16) Å, *b* = 15.5011(2) Å, *c* = 17.43758(17) Å, *V* = 3427.00(7) Å^3, Z = 8, T = 100 K, μ(CuKα) = 4.122 mm^{-1}, *Dcalc* = 1.377 g/cm^3, 22958 reflections measured (4.505° ≤ 2Θ ≤ 77.877°), 3650 unique (R_{int} = 0.0253) which were used in all calculations. The final R_1 was 0.0270 ($I > 2\sigma(I)$) and wR_2 was 0.0722 (all data). CCDC refcode: 2288798.

Crystal data for **10**, $C_{16}H_{34}BrO_2P$ (*M* = 369.31 g/mol): monoclinic, space group *P*2$_1$/n (no. 14), *a* = 8.78124(3) Å, *b* = 15.12725(5) Å, *c* = 14.11123(5) Å, *β* = 92.5824(3)°, *V* = 1872.575(11) Å^3, Z = 4, T = 100 K, μ(CuKα) = 3.791 mm^{-1}, *Dcalc* = 1.310 g/cm^3, 48199 reflections measured (4.287° ≤ 2Θ ≤ 77.878°), 3997 unique (R_{int} = 0.0322) which were used in all calculations. The final R_1 was 0.0221 ($I > 2\sigma(I)$) and wR_2 was 0.0560 (all data). CCDC refcode: 2288799.

Crystal data for **11**, $C_{17}H_{36}BrO_2P$ (*M* = 383.34 g/mol): orthorhombic, space group *P*bca (no. 61), *a* = 16.11687(9) Å, *b* = 14.80976(11) Å, *c* = 16.89922(11) Å, *V* = 4033.62(5) Å^3, Z = 8, T = 100 K, μ(CuKα) = 3.538 mm^{-1}, *Dcalc* = 1.262 g/cm^3, 29878 reflections measured (4.826° ≤ 2Θ ≤ 77.902°), 4275 unique (R_{int} = 0.0326) which were used in all calculations. The final R_1 was 0.0227 ($I > 2\sigma(I)$) and wR_2 was 0.0596 (all data). CCDC refcode: 2288800.

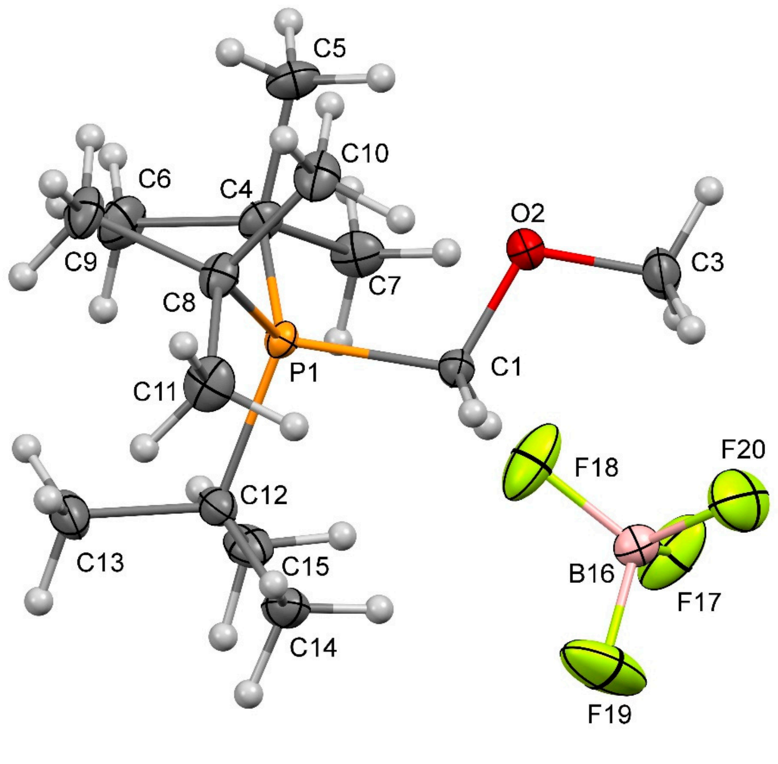

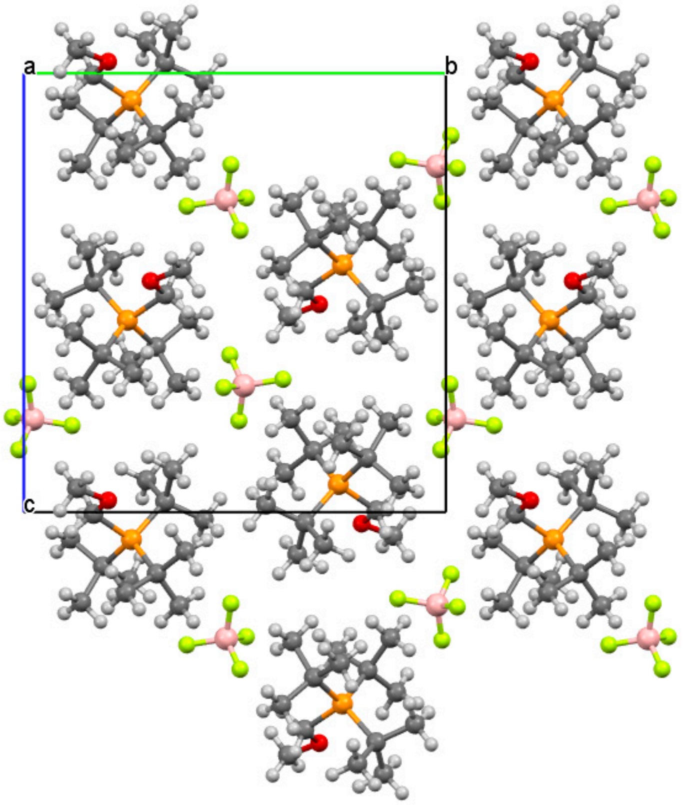

Figure S61. Molecular structure of **2** in crystal and fragment of the crystal packing (view along the *0a* axis). Atoms are represented by spheres indicating their isotropic thermal displacements (ρ = 50%).

Table S1. Selected bond length (Å) and angle (°) in the crystals for compounds **2**.

Compound 2			
Bond (Å)			
Bond	Bond length, Å	Bond	Bond length, Å
P(1)-C(1)	1.8309(11)	F(17)-B(16)	1.3820(15)
P(1)-C(4)	1.8780(12)	F(18)-B(16)	1.3845(16)
P(1)-C(8)	1.8798(11)	F(19)-B(16)	1.3729(16)
P(1)-C(12)	1.8802(12)	F(20)-B(16)	1.3816(16)
O(2)-C(1)	1.4166(13)		
O(2)-C(3)	1.4258(14)		
Angle (°)			
Angle	Angle value, °	Angle	Angle value, °
C(1)-P(1)-C(4)	107.12(5)	F(17)-B(16)-F(18)	108.24(10)
C(1)-P(1)-C(8)	107.36(5)	F(19)-B(16)-F(17)	109.64(11)
C(1)-P(1)-C(12)	102.82(5)	F(19)-B(16)-F(18)	110.23(12)
C(1)-O(2)-C(3)	110.09(9)	F(19)-B(16)-F(20)	108.26(11)
O(2)-C(1)-P(1)	110.71(7)	F(20)-B(16)-F(17)	110.69(12)
C(4)-P(1)-C(8)	112.77(5)	F(20)-B(16)-F(18)	109.77(11)

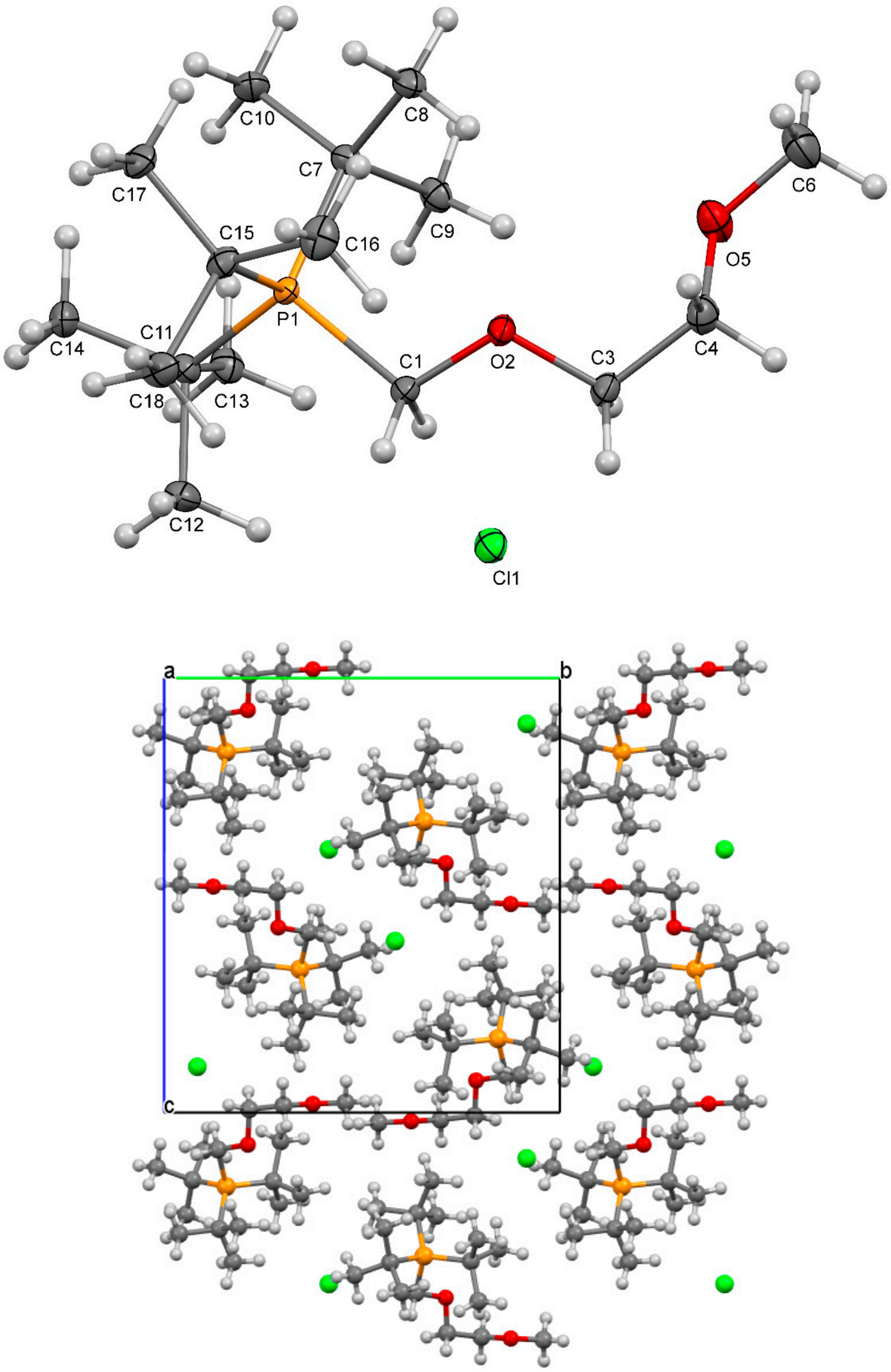

Figure S62. Molecular structure of **4** in crystal and fragment of the crystal packing (view along the *0a* axis). Atoms are represented by spheres indicating their isotropic thermal displacements (ρ = 50%).

Table S2. Selected bond length (Å) and angle (°) in the crystals for compounds **4**.

Compound 4			
Bond (Å)			
Bond	Bond length, Å	Bond	Bond length, Å
P(1)-C(1)	1.8327(11)	O(2)-C(3)	1.4308(13)
P(1)-C(11)	1.8811(11)	O(5)-C(4)	1.4186(15)

P(1)-C(7)	1.8774(11)	O(5)-C(6)	1.4115(16)
P(1)-C(15)	1.8764(11)	C(3)-C(4)	1.5011(16)
O(2)-C(1)	1.4236(13)		
Angle (°)			
Angle	Angle value, °	Angle	Angle value, °
C(1)-P(1)-C(11)	104.02(5)	C(1)-O(2)-C(3)	111.52(8)
C(1)-P(1)-C(7)	106.99(5)	C(6)-O(5)-C(4)	111.57(10)
C(1)-P(1)-C(15)	107.54(5)	O(2)-C(3)-C(4)	107.98(9)
C(7)-P(1)-C(11)	112.11(5)	O(5)-C(4)-C(3)	110.54(10)
O(2)-C(1)-P(1)	109.50(7)		

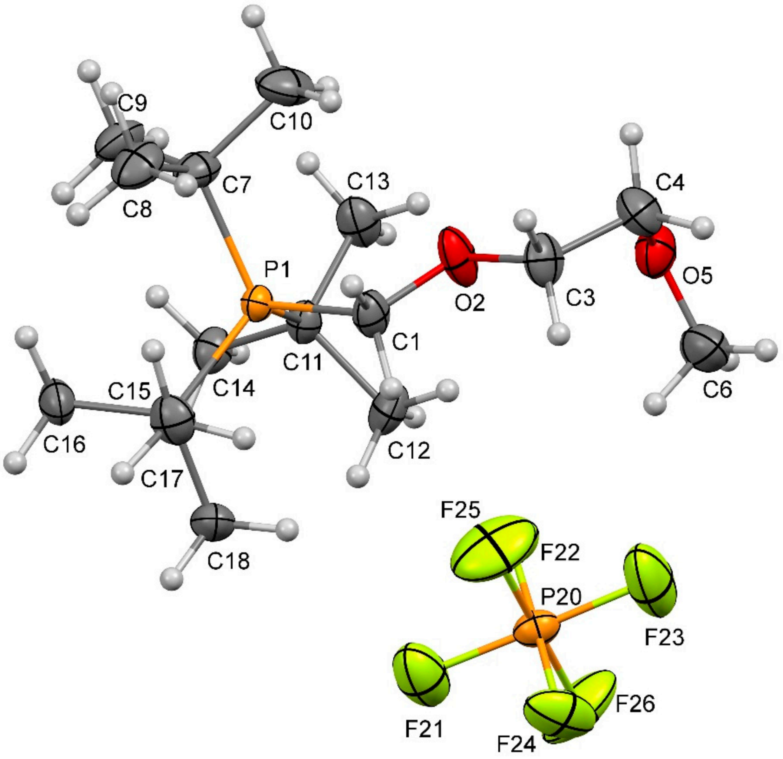

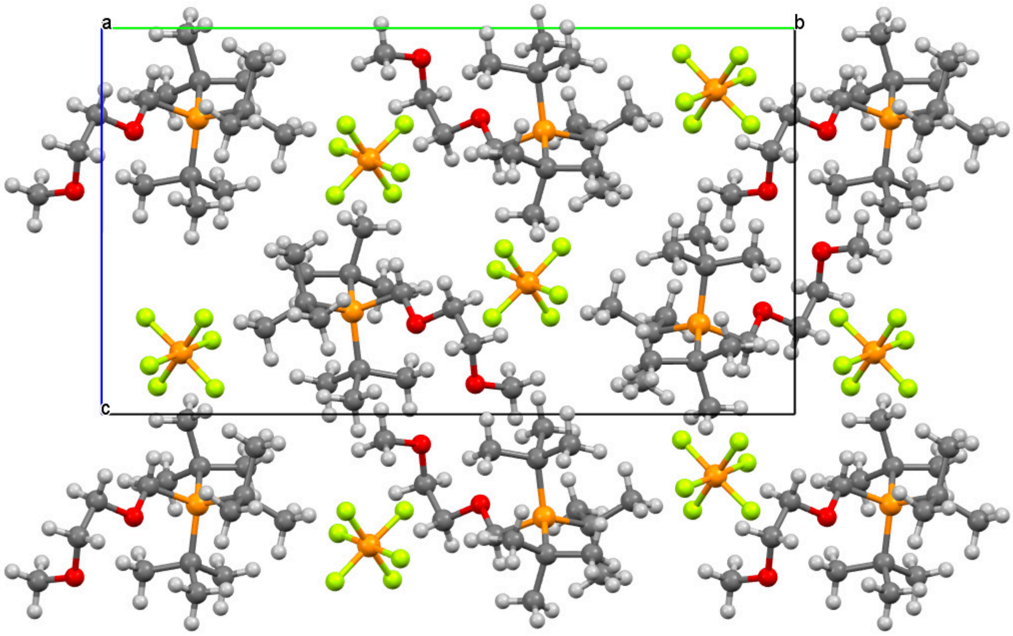

Figure S63. Molecular structure of **6** in crystal and fragment of the crystal packing (view along the *0a* axis). Atoms are represented by spheres indicating their isotropic thermal displacements (ρ = 50%). Hexafluorophosphate-anion is disordered over two positions with the contribution of main component of 0.882(2). Terminal fragment O5-C6 is disordered over two positions with the contribution of main component of 0.525(3).

Table S3. Selected bond length (Å) and angle (°) in the crystals for compounds **6**.

Compound 6			
Bond (Å)			
Bond	Bond length, Å	Bond	Bond length, Å
P(1)-C(1)	1.8315(19)	O(2)-C(3)	1.431(2)
P(1)-C(7)	1.8798(19)	O(5)-C(6)	1.385(18)
P(1)-C(11)	1.8759(19)	O(5)-C(4)	1.438(4)
P(1)-C(15)	1.8781(19)	C(3)-C(4)	1.504(3)
O(2)-C(1)	1.407(2)		
Angle (°)			
Angle	Angle value, °	Angle	Angle value, °
C(1)-P(1)-C(7)	107.29(10)	O(2)-C(3)-C(4)	109.13(17)
C(1)-P(1)-C(11)	108.45(9)	O(5)-C(4)-C(3)	111.6(2)
C(1)-P(1)-C(15)	102.40(9)	C(6)-O(5)-C(4)	116.0(11)
C(1)-O(2)-C(3)	110.36(15)	O(2)-C(1)-P(1)	111.24(13)

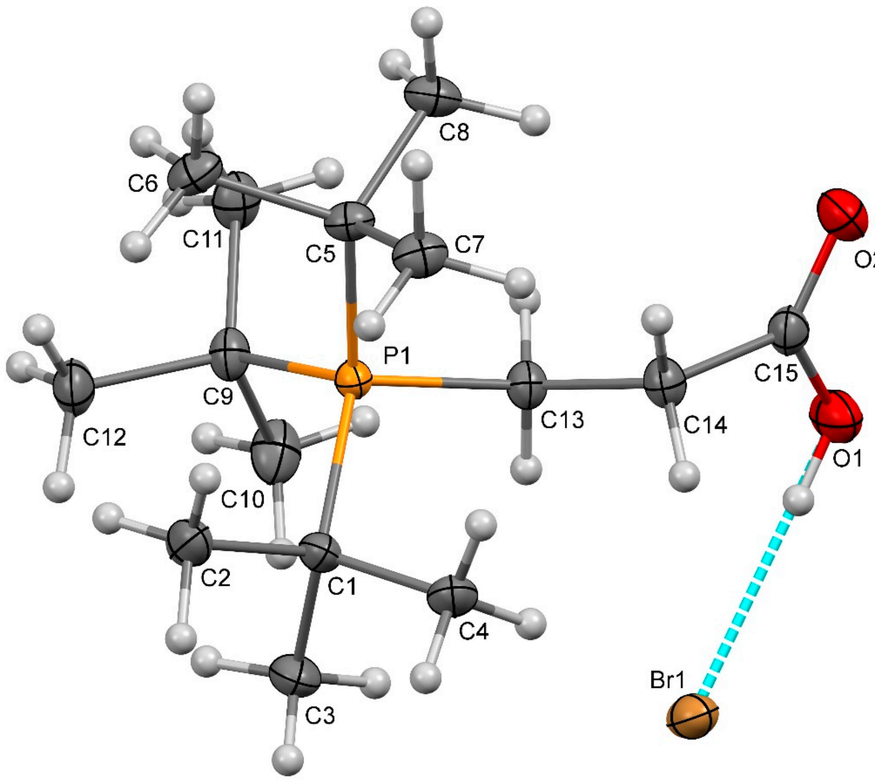

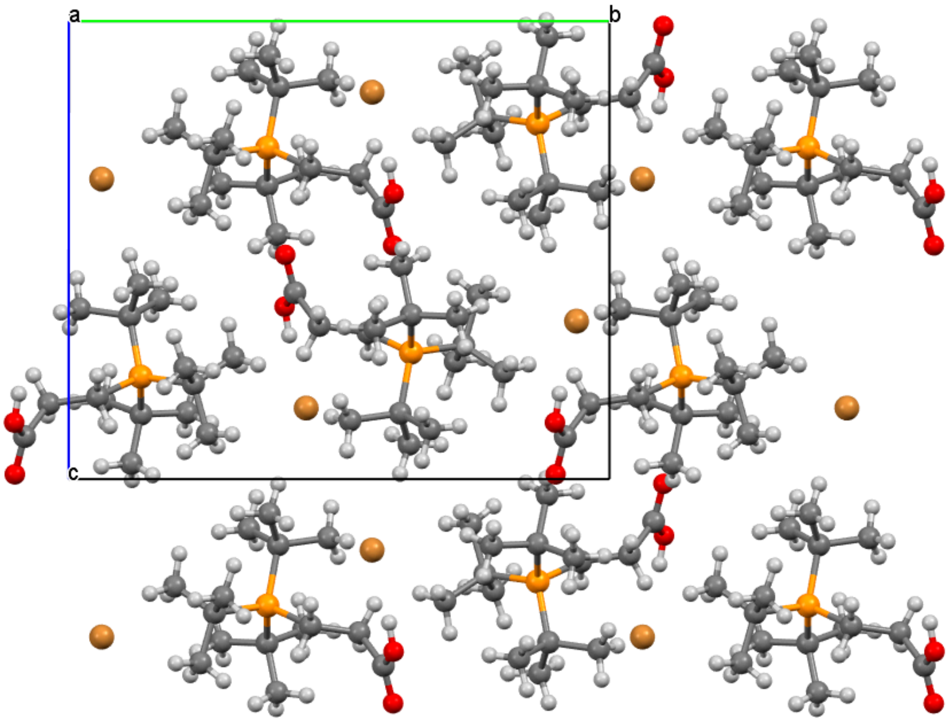

Figure S64. Molecular structure of **7** in crystal and fragment of the crystal packing (view along the *0a* axis). Atoms are represented by spheres indicating their isotropic thermal displacements (ρ = 50%). Hydrogen bond O-H...Br is represented by the cyan dashed line.

Table S4. Selected bond length (Å) and angle (°) in the crystals for compounds **7**.

Compound 7			
Bond (Å)			
Bond	Bond length, Å	Bond	Bond length, Å
P(1)-C(1)	1.8820(14)	C(14)-C(15)	1.514(2)
P(1)-C(5)	1.8837(15)	C(15)-O(1)	1.325(2)
P(1)-C(9)	1.8913(15)	C(15)-O(2)	1.2089(19)
P(1)-C(13)	1.8229(15)	C(13)-C(14)	1.539(2)
Angle (°)			
Angle	Angle value, °	Angle	Angle value, °
C(13)-P(1)-C(1)	109.89(7)	C(14)-C(13)-P(1)	120.19(10)
C(13)-P(1)-C(1)	109.89(7)	C(15)-C(14)-C(13)	110.25(12)
C(13)-P(1)-C(1)	109.89(7)	O(1)-C(15)-C(14)	117.51(13)
C(13)-P(1)-C(5)	108.78(7)	O(2)-C(15)-C(14)	122.33(14)
C(13)-P(1)-C(9)	101.26(7)	O(2)-C(15)-O(1)	120.15(14)

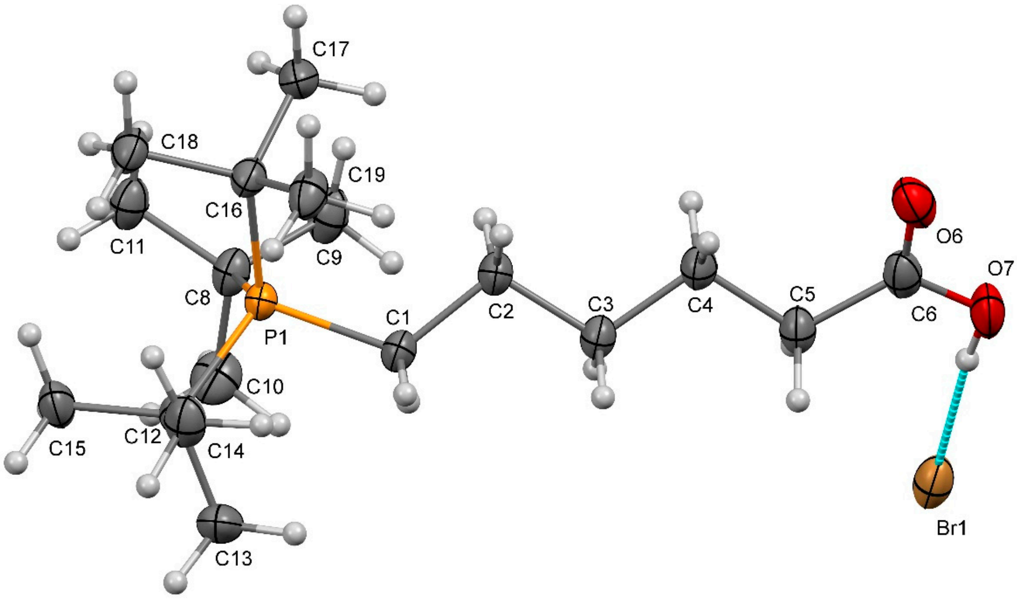

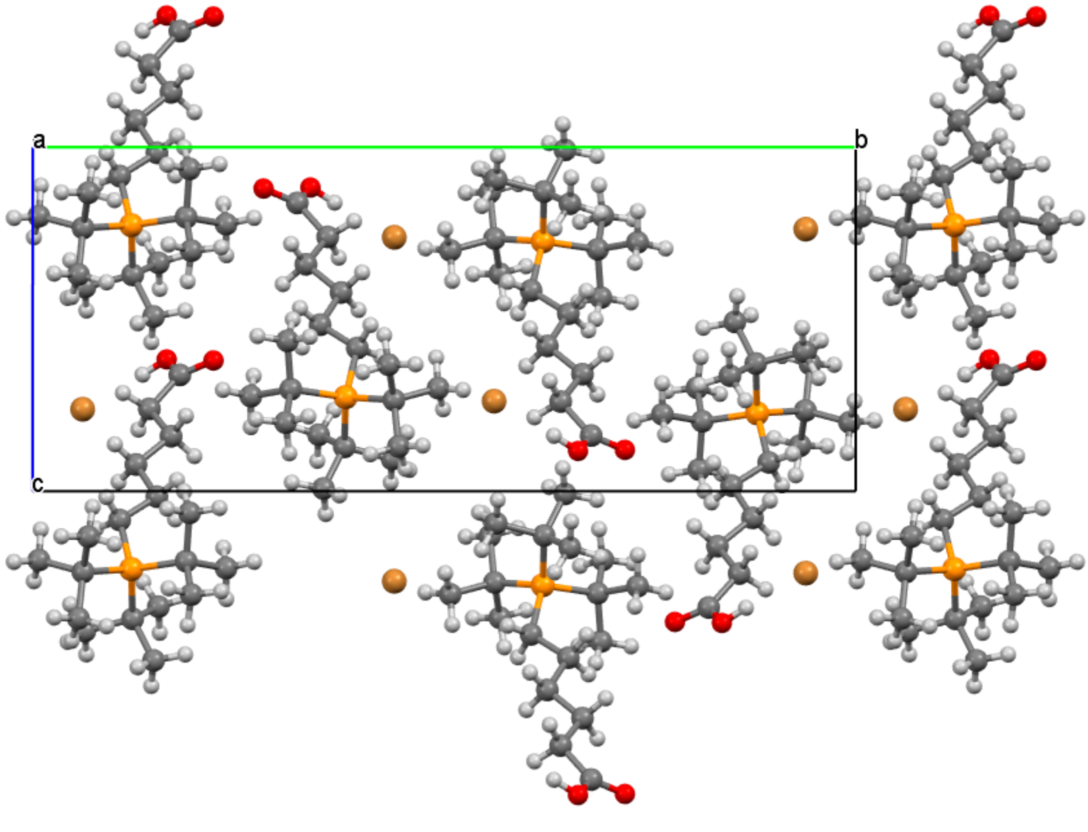

Figure S65. Molecular structure of **8** in crystal and fragment of the crystal packing (view along the *0a* axis). Atoms are represented by spheres indicating their isotropic thermal displacements (ρ = 50%). Hydrogen bond O-H…Br is represented by the cyan dashed line.

Table S5. Selected bond length (Å) and angle (°) in the crystals for compounds **8**.

Compound 8			
Bond (Å)			
Bond	Bond length, Å	Bond	Bond length, Å
P(1)-C(1)	1.8237(19)	C(1)-C(2)	1.539(3)

P(1)-C(8)	1.884(2)	C(2)-C(3)	1.524(3)
P(1)-C(12)	1.883(2)	C(3)-C(4)	1.529(3)
P(1)-C(16)	1.879(2)	C(4)-C(5)	1.519(3)
O(6)-C(6)	1.207(3)	C(5)-C(6)	1.503(3)
O(7)-C(6)	1.336(3)		
Angle (°)			
Angle	Angle value, °	Angle	Angle value, °
C(1)-P(1)-C(8)	106.81(9)	C(2)-C(3)-C(4)	112.23(17)
C(1)-P(1)-C(12)	104.51(9)	C(2)-C(3)-C(4)	112.23(17)
C(1)-P(1)-C(16)	109.36(9)	C(6)-C(5)-C(4)	114.36(19)
C(2)-C(1)-P(1)	121.31(14)	O(6)-C(6)-O(7)	120.0(2)
C(3)-C(2)-C(1)	109.39(16)	O(6)-C(6)-C(5)	124.3(2)
		O(7)-C(6)-C(5)	115.7(2)

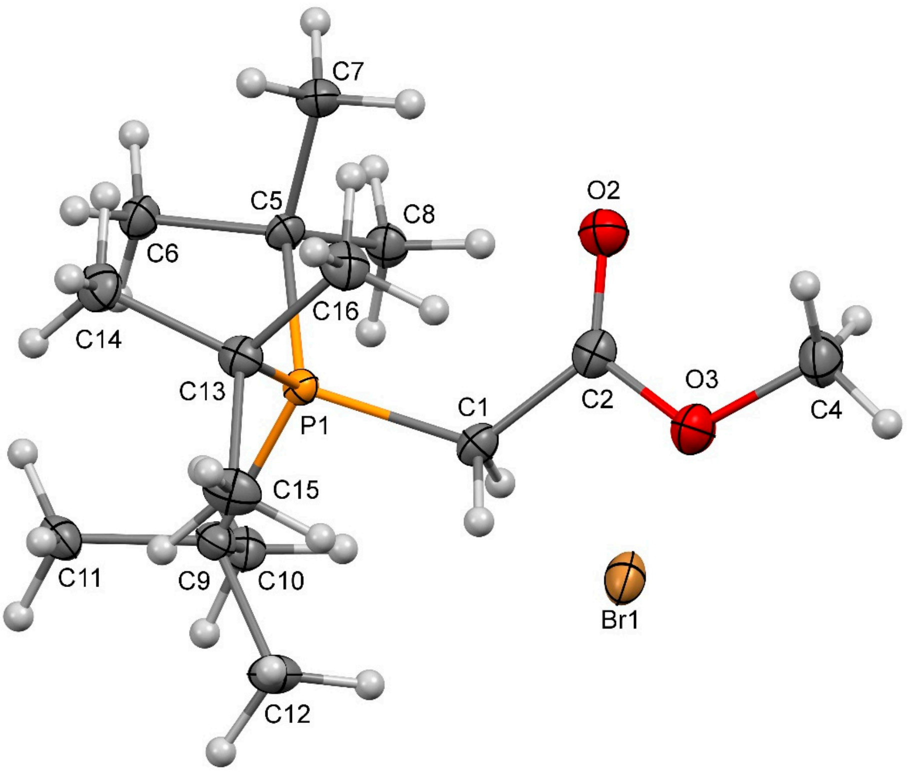

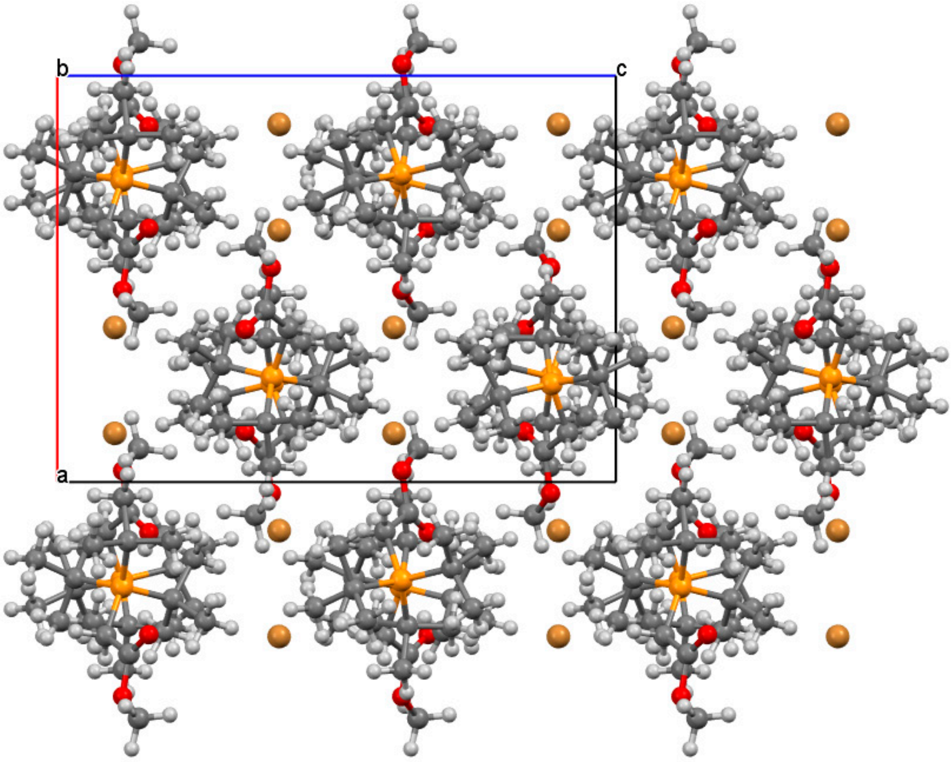

Figure S66. Molecular structure of **9** in crystal and fragment of the crystal packing (view along the *0b* axis). Atoms are represented by spheres indicating their isotropic thermal displacements (ρ = 50%).

Table S6. Selected bond length (Å) and angle (°) in the crystals for compounds **9**.

Compound 9			
Bond (Å)			
Bond	Bond length, Å	Bond	Bond length, Å
P(1)-C(9)	1.8957(16)	O(3)-C(4)	1.455(2)
P(1)-C(5)	1.8808(16)	O(2)-C(2)	1.203(2)
P(1)-C(1)	1.8255(16)	O(3)-C(2)	1.339(2)
P(1)-C(13)	1.8822(16)		
Angle (°)			
Angle	Angle value, °	Angle	Angle value, °
C(1)-P(1)-C(9)	101.33(7)	C(2)-C(1)-P(1)	123.74(12)
C(1)-P(1)-C(5)	110.98(7)	O(2)-C(2)-O(3)	123.70(16)
C(1)-P(1)-C(13)	107.30(8)	O(2)-C(2)-C(1)	127.19(16)
C(2)-O(3)-C(4)	115.99(14)	O(3)-C(2)-C(1)	109.05(14)

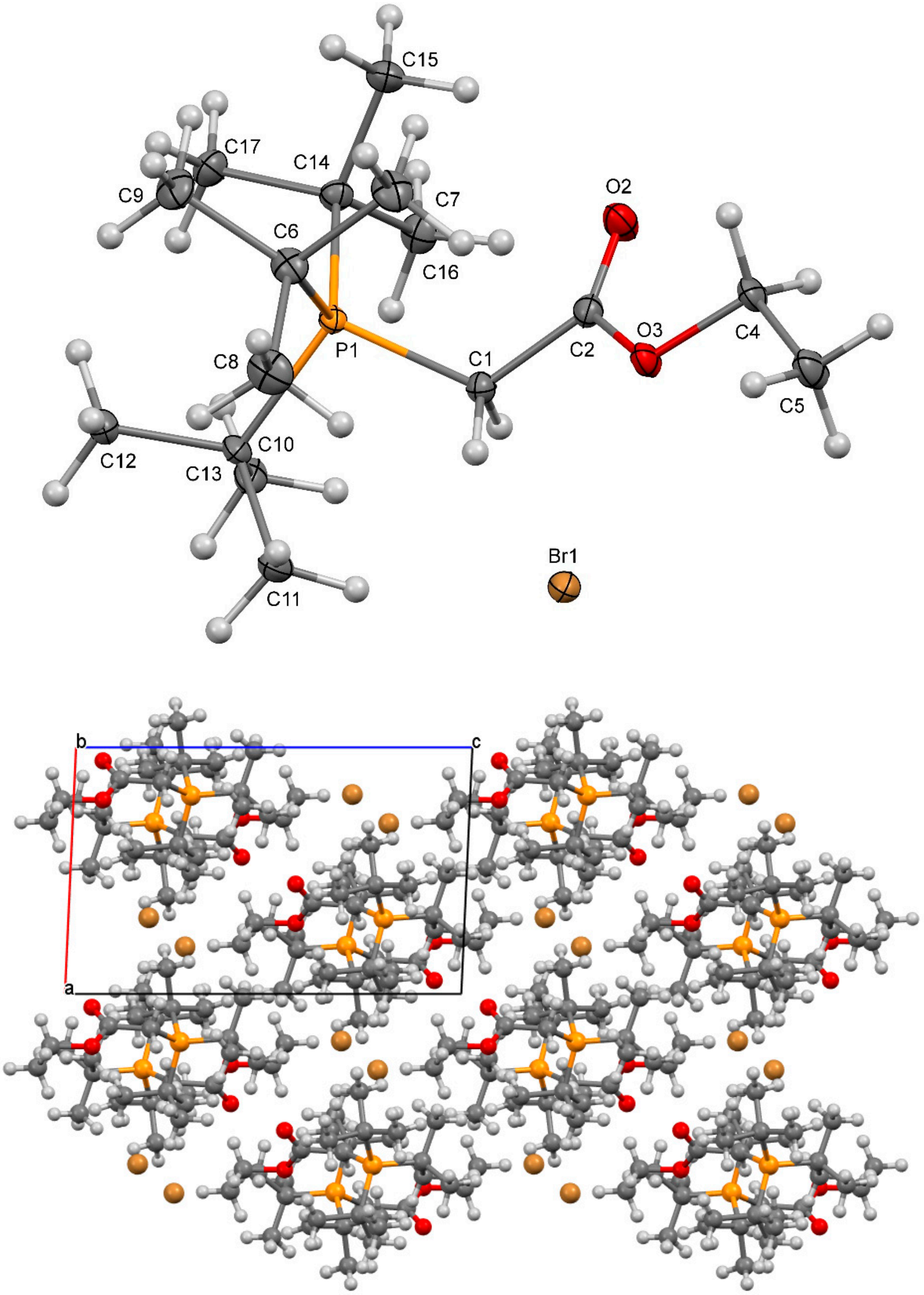

Figure S67. Molecular structure of **10** in crystal and fragment of the crystal packing (view along the *0b* axis). Atoms are represented by spheres indicating their isotropic thermal displacements (ρ = 50%).

Table S7. Selected bond length (Å) and angle (°) in the crystals for compounds **10**.

Compound 10			
Bond (Å)			
Bond	Bond length, Å	Bond	Bond length, Å
P(1)-C(1)	1.8308(13)	O(3)-C(2)	1.3388(16)
P(1)-C(6)	1.8759(13)	O(3)-C(4)	1.4700(16)
P(1)-C(10)	1.8936(13)	C(1)-C(2)	1.5185(17)

P(1)-C(14)	1.8844(13)	C(4)-C(5)	1.5040(19)
O(2)-C(2)	1.2024(17)		
Angle (°)			
Angle	Angle value, °	Angle	Angle value, °
C(1)-P(1)-C(6)	107.03(6)	C(2)-C(1)-P(1)	119.91(9)
C(1)-P(1)-C(10)	102.60(6)	O(2)-C(2)-O(3)	124.16(12)
C(1)-P(1)-C(14)	109.96(6)	O(2)-C(2)-C(1)	126.07(12)
C(2)-O(3)-C(4)	115.26(10)	O(3)-C(2)-C(1)	109.70(11)
		O(3)-C(4)-C(5)	107.07(11)

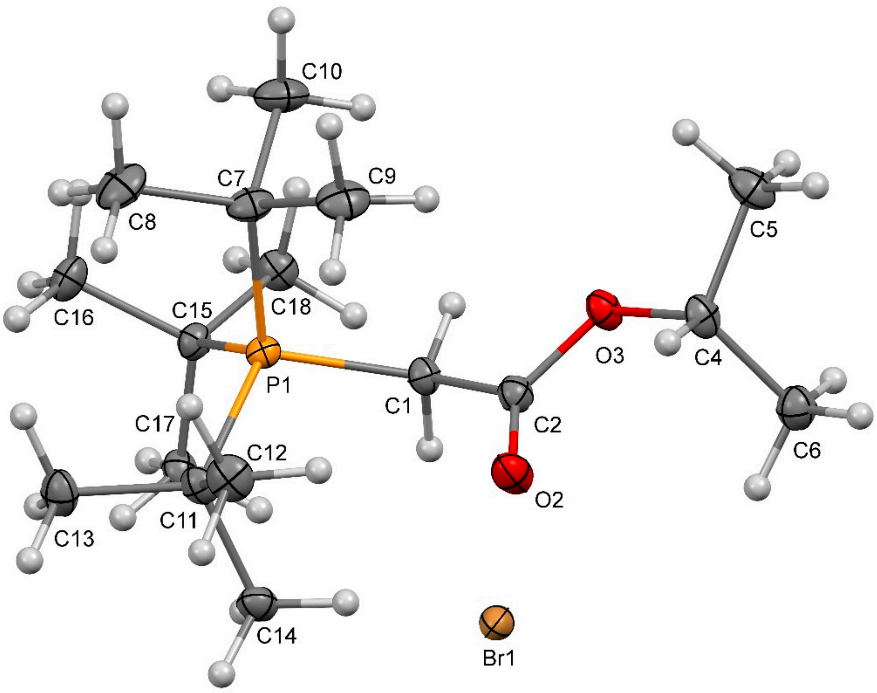

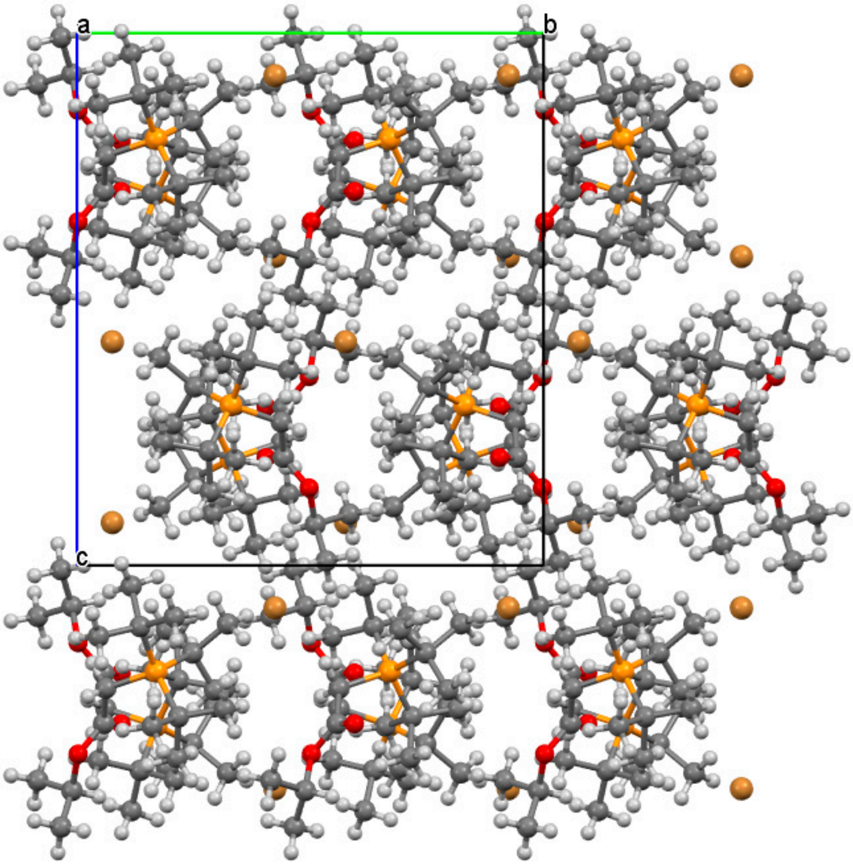

Figure S68. Molecular structure of **11** in crystal and fragment of the crystal packing (view along the *0b* axis). Atoms are represented by spheres indicating their isotropic thermal displacements (ρ = 50%).

Table S8. Selected bond length (Å) and angle (°) in the crystals for compounds **11**.

Compound 11			
Bond (Å)			
Bond	Bond length, Å	Bond	Bond length, Å
P(1)-C(1)	1.8229(13)	C(4)-C(6)	1.509(2)
P(1)-C(15)	1.8909(13)	O(2)-C(2)	1.2037(18)
P(1)-C(11)	1.8849(13)	O(3)-C(2)	1.3412(16)
P(1)-C(7)	1.8768(14)	O(3)-C(4)	1.4770(15)
C(4)-C(5)	1.5124(19)	C(1)-C(2)	1.5169(18)
Angle (°)			
Angle	Angle value, °	Angle	Angle value, °
C(1)-P(1)-C(15)	102.25(6)	C(2)-C(1)-P(1)	120.67(9)
C(1)-P(1)-C(11)	110.52(6)	O(2)-C(2)-O(3)	124.84(12)
C(1)-P(1)-C(7)	107.41(6)	O(2)-C(2)-C(1)	125.49(12)
C(2)-O(3)-C(4)	116.42(10)	O(3)-C(2)-C(1)	109.62(11)
O(3)-C(4)-C(5)	105.64(10)	C(6)-C(4)-C(5)	113.29(13)
O(3)-C(4)-C(6)	109.00(11)		

Table S9. Activity of oxy-QPS against non-pathogenic microbes*.

oxy-QPS	**MIC** – minimal inhibition concentration, µg/mL		
	Bs	*Lp*	*Sc*
**1**	500	>500	>500
**4**	>500	>500	>500
**5**	>500	>500	>500
**7**	500	>500	>500
**8**	>500	>500	>500
**9**	250	>500	>500
**10**	>500	>500	>500
**11**	>500	>500	>500
**12**	>500	>500	>500
Amoxicillin	15.6	-	-
**MBC** and **MFC** – bactericide and fungicide activity, µg/mL			
**1**	500	>500	>500
**4**	>500	>500	>500
**5**	>500	>500	>500
**7**	>500	>500	>500
**8**	>500	>500	>500
**9**	250	>500	>500
**10**	>500	>500	>500
**11**	>500	>500	>500
**12**	>500	>500	>500
Amoxicillin	31.3	-	-

*The experiment was repeated three times

Table S10. Ecotoxicity of oxy-QPS to *D. magna*.

oxy-QPS	The concentration of solution, %	Dead, %	Acute aquatic hazard category
**1**	0.001	0	Non-toxic
	0.01	30	
**4**	0.01	63	Acute 3
**5**	0.001	7	Acute 3
**7**	0.001	3	Non-toxic
	0.01	20	
**11**	0.001	0	Acute 3
	0.01	50	
**12**	0.01	100	Acute 3
	0.001	7	
